# Resilience of countries to COVID-19 correlated with trust

**DOI:** 10.1038/s41598-021-03358-w

**Published:** 2022-01-06

**Authors:** Timothy M. Lenton, Chris A. Boulton, Marten Scheffer

**Affiliations:** 1 Global Systems Institute, University of Exeter, Exeter, UK; 2 Wageningen University, Wageningen, The Netherlands; 3 Santa Fe Institute, Santa Fe, NM USA

**Keywords:** Ecological epidemiology, Public health, Statistics

## Abstract

We characterized > 150 countries’ resilience to COVID-19 as the nationwide decay rate of daily cases or deaths from peak levels. Resilience to COVID-19 varies by a factor of ~ 40 between countries for cases/capita and ~ 25 for deaths/capita. Trust within society is positively correlated with country-level resilience to COVID-19, as is the adaptive increase in stringency of government interventions when epidemic waves occur. By contrast, countries where governments maintain greater background stringency tend to have lower trust within society and tend to be less resilient. All countries where > 40% agree “most people can be trusted” achieve a near complete reduction of new cases and deaths, but so do several less-trusting societies. As the pandemic progressed, resilience tended to decline, as adaptive increases in stringency also declined. These results add to evidence that trust can improve resilience to epidemics and other unexpected disruptions, of which COVID-19 is unlikely to be the last.

## Introduction

One of the big questions in the wake of the COVID-19 pandemic is why some countries seem to have been so much more successful than others in suppressing the waves of infections and deaths. For example, China^1^ and Bhutan^2^ performed well, whilst India^3^ and Myanmar^4^ performed poorly. Here we explore this issue using well-established ecological measures for the response of complex systems to perturbation^5^. In particular, ‘resilience’ describes the rate of recovery of a system from perturbation back towards a presumed, pre-existing stable state—here zero infection and associated deaths—where rapid recovery equals high resilience. The dynamics of infectious disease are such that if the basic reproduction number^6^, *R*_0_ (the number of secondary infections produced by a single infected individual) exceeds 1, then exponential growth of new cases will result—i.e. an epidemic (although the lag between primary and secondary infection events complicates the relationship between *R*_0_ and growth rate of cases^7^). Estimates^8^ that COVID-19 has *R*_0_ ~ 4.5 imply the potential for rapid exponential spread. In natural populations, such explosive spread typically results in an infection spreading throughout a population, until acquired immunity and/or mortality stabilises and ultimately reduces case numbers.

In contemporary human societies confronted by COVID-19, a host of responses have been used to try and limit the effective reproduction number^6^, *R*_e_ (the number of people that can be infected by an individual at any specific time) and hence the spread of infection. Government interventions to limit social contact have been shown to reduce *R*_e_ with a ~ 1–3 week lag, although few have a statistically significant effect^9^. When *R*_e_ = 1 is achieved, new cases peak, and the absolute size of the peak can be taken as an ecological measure^5^ of ‘resistance’ to perturbation, where lower peak equals higher resistance. When *R*_e_ < 1 is achieved, case numbers will decay, and if *R*_e_ is also approximately constant, the decay is exponential (albeit again with a lag^7^) and the decay rate quantifies ‘resilience’. Deaths should track new cases, with a lag, if mortality rate is constant. Usually, the original stable state of zero infection is not recovered when efforts to limit the spread of infection (*R*_e_) are relaxed and therefore cases may start to rise again. To characterize the social capacity to resist this temptation and maintain measures even if infections go down, we also consider the proportional ‘reduction’ from peak to minimum as a metric.

We focus on the role of social and cultural factors in determining country-level resilience to COVID-19. We test for demographic and public-health-related factors some of which significantly influence spread of the infection in within-country analysis^10^. Then inspired by suggestions^11,12^ that variation in resilience to COVID-19 partly reflects variation in the nature and strength of the ‘social contract’ across countries, we look at the effects of government interventions and trust. We consider the stringency of deliberate government interventions to limit social contact and thus *R*_e_ which is expected to result in greater resilience^9^. Then we test for the effect of generalised trust within societies, and of confidence in specific political and government organisations, which have been proposed to support greater resilience^13^. We also examine whether Hofstede’s six cultural dimensions^14^ of power distance, individualism, uncertainty avoidance, masculinity, long-term orientation and indulgence affect resilience.

## Methods

We take a deliberately coarse-grained approach to measuring resilience as whole-country recovery rates of COVID-19 new cases and deaths, inspired by the observation that these often appear close to exponential decays (Supplementary Fig. 1). All methods were carried out in accordance with relevant guidelines and regulations.

### Data sources

We analysed openly available COVID-19 data for daily confirmed cases and deaths^15^, and testing^16^. We normalised cases and deaths by population size, to consider cases/capita and deaths/capita, and smoothed the data to minimise issues such as weekly cycles in reporting. Given large known variations in testing intensity (tests/capita) over time and space, we also considered cases/tests, where testing data is available—but recognise that testing intensity varies over time in different ways in different countries^16^, which can introduce biases. All data were sourced from Our World in Data COVID-19 dataset^17^. Raw data on daily confirmed cases and deaths for all countries is from the COVID-19 Data Repository by the Center for Systems Science and Engineering (CSSE) at Johns Hopkins University^15,18^. Testing data is from Our World in Data^16^. Note that the list of ‘countries’ includes the disputed territory of Kosovo (OWID_KOS). The world aggregate (OWID_WRL) is removed from our comparative analysis of countries. We use a Kernal smoothing function with a bandwidth of 10 to smooth the daily time series of cases/capita, tests/capita, and deaths/capita. To create cases/tests time series, we divide the smoothed cases/capita time series by the test/capita time series. Peaks in time series were detected by eye, looking across all time series (cases/capita, deaths/capita, cases/tests) available for each country. For a given country, peaks were manually lined up across time series. Resistance is taken as the negative of the maxima in the smoothed time series (such that a smaller peak height corresponds to higher resistance).

### Resilience calculation

Resilience is estimated from the interval of data from a maximum to the next minimum in each smoothed time series (or the end of the time series if it happens first). The data were natural logged and linear regression used to determine the goodness of fit of an exponential decay. Most of the results cluster at r^2^ ≥ 0.8, with a clear drop off and scattering of r^2^ values below 0.8. Visual inspection confirmed that r^2^ ≥ 0.8 captures cases of reasonably exponential decay for further analysis. A more stringent cut-off of r^2^ ≥ 0.9 was also considered but limits the sample size for further analysis. Reduction is calculated as minimum divided by preceding maximum in the smoothed timeseries and is not reported if the fit of decay r^2^ < 0.8 or the end of the time series occurs before a minimum. All instances of peaks and decays are grouped in a combined dataset (so the same country exhibiting multiple peaks and decays will appear more than once). A dataset of first peaks only was also considered but limits the sample size.

Having established a resilience dataset we checked for temporal and spatial correlations then focused on social and cultural factors as potential predictors.

### Test for spatial autocorrelation

To test for spatial autocorrelation, we first assign a geographical midpoint to each country by taking the midpoints of the min and max of the latitude and longitude of its border. Then we measure the distance between these midpoints for every pair of countries. We correlate this measure of distance between countries with the difference in resilience between them for the decay from the first peak.

### Demographic, financial, public-health factors

Data were sourced from Our World in Data COVID-19 dataset^17^. Population and life expectancy are from United Nations, Department of Economic and Social Affairs, Population Division, World Population Prospects 2019 Revision^19^. Population density is from the World Bank—World Development Indicators^20^. Country area is from dividing population by population density (to maintain internal consistency). GDP/capita is from the Maddison Project Database, version 2018^21^. Human Development Index (HDI) is from the United Nations Development Programme (UNDP)^22^. Hospital beds (per 1000) for the most recent year available since 2010 is compiled by Our World in Data from multiple sources^23^.

### Government stringency index

Data are from the Oxford COVID-19 Government Response Tracker^24,25^ (OxCGRT) as reported by Our World in Data^17^. It is a composite measure based on nine response indicators, rescaled to a value from 0 to 100 (100 = strictest). If policies vary at the subnational level, the index is shown as the response level of the strictest sub-region. The response indicators are: school closing (C1), workplace closing (C2), cancel public events (C3), restrictions on gatherings (C4), close public transport (C5), stay at home requirements (C6), restrictions on internal movement (C7), international travel controls (C8), and public info campaigns (H1). For each country, ‘mean stringency’ was calculated as the average across the whole time series since the start of the pandemic. ‘Background stringency’ was calculated as the average over the intervals when fitted decay intervals are not occurring. ‘Decay stringency’ was calculated as the average over each fitted decay interval. ‘Adaptive stringency’ was calculated for each decay interval as the difference from a ‘pre stringency’ to ‘decay stringency’—where ‘pre stringency’ was averaged over the preceding interval, starting either at the start of the timeseries or at the end of a previous decay interval. Stringency metrics were calculated separately for cases/capita and deaths/capita as they have separate decay intervals (except mean stringency which is the same in both cases). However, even when considering resilience of deaths/capita the correlation results are comparable or better using stringency measures calculated for cases/capita—presumably because stringent policy interventions typically respond to cases data and the response of deaths lags weeks behind. Hence, we focus on stringency measures calculated for cases, even when considering resilience of deaths.

### Trust and confidence in organisations

Trust and confidence in organisations data is from the World Values Survey^26,27^ Wave 7 (2017–2020). Trust data is from Q57 and is the percentage of respondents who agree with the statement “most people can be trusted” (for 79 countries). This is often referred to in the literature as generalised trust, and sometimes as unspecified trust. For confidence in organisations data we selected questions pertinent to the social contract; Q71 the government, Q72 political parties, Q73 parliament, and Q76 elections. Questions are of the common form; “could you tell me how much confidence you have in them: is it a great deal of confidence, quite a lot of confidence, not very much confidence or none at all”. The answer codes were assigned scores of 1 (a great deal), 2/3, 1/3, 0 (none at all) respectively. Data are in the form of percentage of the population of each country assigned to each answer, alongside missing and “don’t know” answers. These percentages were reweighted to sum to 1 across the four answers, then multiplied by the answer scores and summed up, to give a single aggregate confidence score (0–1) for each country.

### Hofstede dimensions

Data for the Hofstede Dimensions is from the dimension data matrix (version 2015 12 08)^14,28^. The six dimensions are defined as follows: (i) *Power distance* is the extent to which the less powerful members of organizations and institutions (like the family) accept and expect that power is distributed unequally. (ii) *Individualism* is the inverse of the degree to which people in a society are integrated into groups. (iii) *Uncertainty avoidance* describes a society's tolerance for ambiguity where societies that score highly opt for stiff codes of behaviour, guidelines, laws, and generally rely on absolute truth, or the belief that one lone truth dictates everything and people know what it is. (iv) *Masculinity* describes preference in society for achievement, heroism, assertiveness and material rewards for success, whilst its counterpart represents a preference for cooperation, modesty, caring for the weak and quality of life. (v) *Long-term orientation* describes connection of the past with the current and future actions/challenges—societies with a high degree in this index view adaptation and circumstantial, pragmatic problem-solving as a necessity. (vi) *Indulgence* describes the degree of freedom that societal norms give to citizens in fulfilling their human desires.

### Pairwise regressions

We use Spearman’s rank correlation coefficient ($\rho$) because not all variables considered are normally distributed and we wanted to detect any non-linear relationships.

### Multiple regression

Variables that are not normally distributed were first log transformed to achieve a normal distribution. The reduction distribution is strongly skewed and there was no improvement in transforming it. We experimented with different sets of independent variables, informed by the pairwise regression analysis in seeking to limit the number of independent variables. We considered both multiple linear regression and logistic regression models but found the logistic model fits were either slightly worse (resilience) or comparable (reduction). Hence for simplicity we present multiple linear regression models throughout. Once models were fitted, we used the step() function in R to optimise the model by Akaike information criterion (AIC), by adding or removing variables until the optimum fit is found.

## Results

Up to 1 December 2020, 156 countries had exhibited at least one peak and then decay of cases/capita (of which 36 had experienced a second peak and decay), 151 countries had exhibited at least one peak and then decay of deaths/capita (of which 32 had experienced a second peak and decay), and 93 countries had sufficient testing data to determine at least one peak and then decay of cases/tests (of which 23 had experienced a second peak and decay). Time-series for all countries and the three metrics are shown in Supplementary Fig. 1. For resilience, having filtered cases of reasonably exponential decay for further analysis (r^2^ ≥ 0.8) and included multiple instances of well-fitted recovery occurring in one country in the dataset, we obtain n = 177 decays for cases/capita, n = 159 for deaths/capita, n = 105 for cases/tests. In a few countries a minimum had not yet been reached by 1 December 2020, so the reduction dataset is smaller (cases/capita n = 165, deaths/capita n = 150, cases/tests n = 101).

### Comparable resilience and reduction of cases and deaths

**Figure 1:**
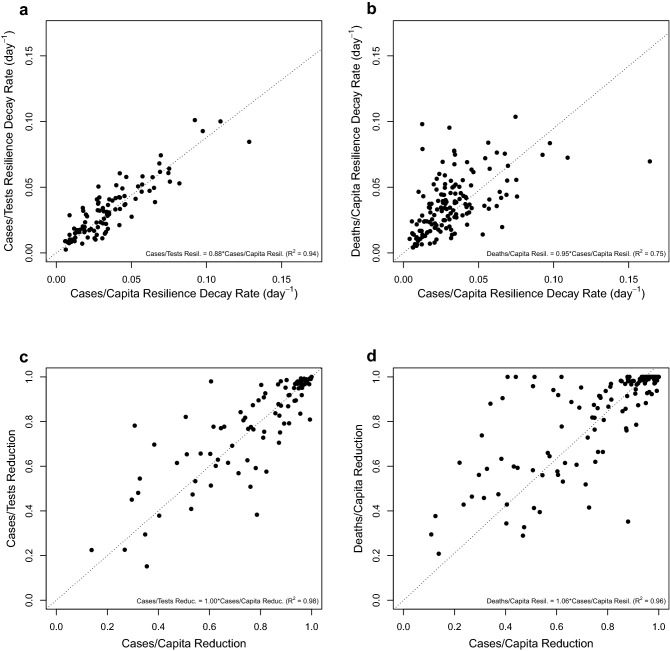
Comparing country-level COVID-19 resilience and reduction results for cases/capita, cases/tests, and deaths/capita. (**a**) resilience of cases/capita vs cases/tests (n = 100, $\rho$ =0.86, p < 0.0001). (**b**) resilience of cases/capita vs deaths/capita (n = 150, $\rho$ =0.61, p < 0.0001). (**c**) reduction of cases/capita vs cases/tests (n = 94, $\rho$ =0.83, p < 0.0001). (**d**) reduction of cases/capita vs deaths/capita (n = 136, $\rho$ =0.76, p < 0.0001). Linear correlation results tied to the origin are given within each figure panel and correspond to the dotted lines.

The relative measures of resilience (rate of decay) and (proportional) reduction of cases should be more reliably estimated than absolute case numbers but could still be biased by variations in testing intensity across time and space. Encouragingly, we find across countries and waves, resilience of cases/capita and cases/tests are strongly positively rank correlated (n = 100, $\rho$ =0.86, p < 0.0001) with linear correlation gradient 0.88 (r^2^ = 0.94) indicating that cases/capita tend to decay slightly faster than cases/tests (Fig. 1a). Resilience of cases/capita and deaths/capita are positively rank correlated (n = 150, $\rho$ =0.61, p < 0.0001) with linear correlation (gradient 0.95, r^2^ = 0.75) indicating cases tend to decay slightly faster than deaths (Fig. 1b). Reduction of cases/capita and cases/tests are also strongly positively rank correlated (n = 94, $\rho$ =0.83, p < 0.0001) with proportional reductions (linear correlation gradient 1.0, r^2^ = 0.98; Fig. 1c). Reduction of cases/capita and deaths/capita are positively rank correlated (n = 136, $\rho$ =0.76, p < 0.0001) with linear correlation (gradient 1.06, r^2^ = 0.96) indicating deaths tend to be reduced slightly more effectively than cases (Fig. 1d). We find that variations between countries in the pattern of testing intensity over time can bias resilience results for cases/tests (see Supplementary Discussion). Therefore, as considering cases/tests also restricts the sample size and does not qualitatively alter later correlation results (see Supplementary Table 1 and Supplementary Discussion), we focus on cases/capita and deaths/capita when considering resilience and reduction.

### Resilience is only weakly correlated with resistance

One might expect that lower resistance (a higher peak) would lead to lower resilience (slower recovery), e.g. because having a greater peak fraction of a country’s population infected provides more sources of further infection. Conversely, greater peak per capita levels of infection and/or deaths might inspire more effective measures and greater social compliance with those measures to bring down infections and deaths. The problem here is reliably estimating the *absolute* measure of resistance. Peak deaths/capita data should be more reliable than peak cases/capita (despite some issues with attributing deaths) because many cases have gone undetected particularly during the first wave, yet somewhat surprisingly peak deaths/capita and peak cases/capita are strongly correlated (n = 150, $\rho$ =0.86, p < 0.0001). There are weak positive correlations between resistance and resilience for deaths/capita, cases/capita, and cases/tests (Supplementary Fig. 2), but high resistance corresponds to a very wide range of resilience, and some countries with low resistance have relatively high resilience, particularly for deaths/capita. Given the weak relationship between resistance and resilience and the problems estimating resistance, we proceed with an independent treatment of resilience from hereon.

### A threshold level of resilience is necessary for successful reduction

**Figure 2:**
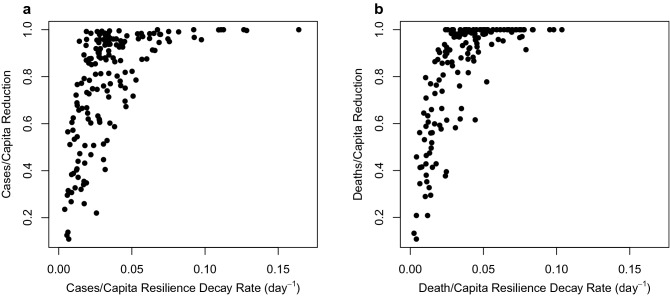
Comparing resilience to, and reduction of, COVID-19 across countries. (**a**) cases/capita (n = 165, $\rho$ =0.70, p < 0.0001), (**b**) deaths/capita (n = 150, $\rho$ =0.78, p < 0.0001).

As would be expected mathematically, reduction (from peak to next minimum) is strongly positively correlated with resilience, following a non-linear relationship (Fig. 2). Really high resilience >  ~ 0.1 d^−1^ (half-life <  ~ 1 week) tends to end in near complete reduction, but few countries have achieved this level of resilience. Instead several countries still achieve a near complete reduction of cases or deaths if they have a resilience of >  ~ 0.02 d^−1^ (half-life <  ~ 1 month). Below that threshold level of resilience, reduction inevitably drops. Thus, poor resilience leads to failure to eliminate cases and deaths.

### Resilience varies hugely between countries

**Figure 3:**
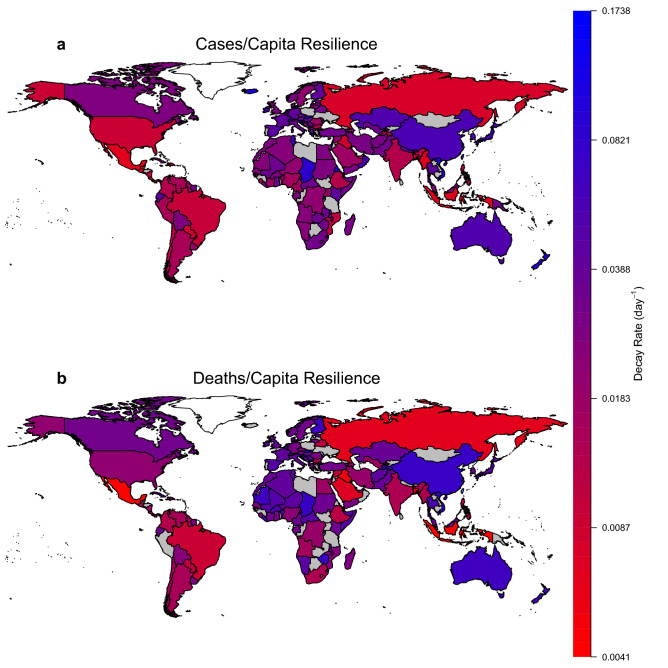
World maps of country-level resilience to COVID-19. Decay rate (d^−1^) from the first peak of: (**a**) cases/capita. (**b**) deaths/capita. In both cases countries are coloured where the fit of an exponential decay has r^2^ ≥ 0.8. Countries in grey either have insufficient data or a poorer fit of exponential decay. Country/region boundaries plotted in R using the ‘maps’ package (ver. 3.3.0; https://CRAN.R-project.org/package=maps).

Resilience of cases/capita, measured as magnitude of decay rate, ranges by a factor of ~ 40, from 0.16 d^−1^ (Mauritius; most resilient) to 0.0041 d^−1^ (Costa Rica; least resilient), corresponding to a half-life of ~ 4 to ~ 170 days (Fig. 3a). Resilience of cases/tests also ranges by a factor of ~ 40 (see Supplementary Discussion). Resilience of deaths/capita, ranges by a factor of ~ 25 from 0.10 d^−1^ (Slovakia; most resilient) to 0.0042 d^−1^ (Indonesia, Mexico, Romania; least resilient) (half-life ~ 7 to 165 days) (Fig. 3b).

Pairwise correlation results are summarised in Table 1 and are robust to analysing just the first peaks in each country (Supplementary Table 2) or using a more stringent fitting of exponential decay (r^2^ ≥ 0.9; Supplementary Table 3)—both of which reduce the sample size.

**Table 1:** Factors correlating with resilience (decay rate) and reduction of COVID-19 cases and deaths across countries. Pairwise Spearman’s rank correlations.

Explanatory variable	Resilience						Reduction					
	Cases/capita			Deaths/capita			Cases/capita			Deaths/capita		
	$\rho$	p	n	$\rho$	p	n	$\rho$	p	n	$\rho$	p	**n**
Day of year of peak	−0.51	< 0.0001	176	−0.43	< 0.0001	159	−0.54	< 0.0001	164	−0.39	< 0.0001	150
Population	−0.23	< 0.01	177	−0.32	< 0.0001	159	−0.22	< 0.01	165	−0.27	< 0.001	150
Country size	−0.24	< 0.01	175	−0.23	< 0.01	157	(−0.14)	−	163	−0.18	< 0.05	148
Population density	(0.08)	−	175	(0.02)	−	157	(−0.05)	−	163	(0.04)	−	148
GDP/capita	0.21	< 0.01	170	(0.03)	−	153	(0.08)	−	158	(0.0)	−	144
Median age	0.26	< 0.001	172	(0.09)	−	154	(0.08)	−	160	(−0.05)	−	145
Life expectancy	0.26	< 0.001	175	(0.15)	−	157	(0.05)	−	163	(0.0)	−	148
Human Develop. Index	0.24	< 0.01	172	(0.09)	−	155	(0.08)	−	160	(0.01)	−	146
Hospital beds	0.29	< 0.001	156	0.19	< 0.05	142	(0.14)	−	145	(0.06)	−	134
Mean stringency	−0.24	< 0.01	167	−0.42	< 0.0001	155	−0.41	< 0.0001	156	−0.48	< 0.0001	146
Decay stringency	0.15	< 0.05	167	(0.01)	−	153	−0.17	< 0.05	156	−0.22	< 0.01	144
Background stringency	−0.30	< 0.0001	167	−0.51	< 0.0001	155	−0.47	< 0.0001	156	−0.56	< 0.0001	146
Adaptive stringency	0.47	< 0.0001	167	0.39	< 0.0001	153	0.29	< 0.001	156	0.21	< 0.05	144
Trust	0.43	< 0.0001	77	0.40	< 0.001	75	0.51	< 0.0001	72	0.48	< 0.0001	72
Power distance	−0.34	< 0.001	109	−0.28	< 0.01	102	−0.22	< 0.05	105	−0.27	< 0.01	97
Individualism	0.21	< 0.05	109	0.27	< 0.01	102	(0.10)	−	105	0.23	< 0.05	97
Masculinity	(0.05)	−	109	(0.0)	−	102	(−0.10)	−	105	(−0.02)	−	97
Uncertainty avoidance	(−0.05)	−	109	−0.23	< 0.05	102	−0.23	< 0.05	105	−0.31	< 0.01	97
Long-term orientation	0.25	< 0.01	129	(0.11)	−	116	(0.06)	−	122	(−0.01)	−	110
Indulgence	(0.01)	−	129	(−0.07)	−	117	(0.15)	−	122	0.28	< 0.01	111

### Temporal but not spatial correlations

**Figure 4:**
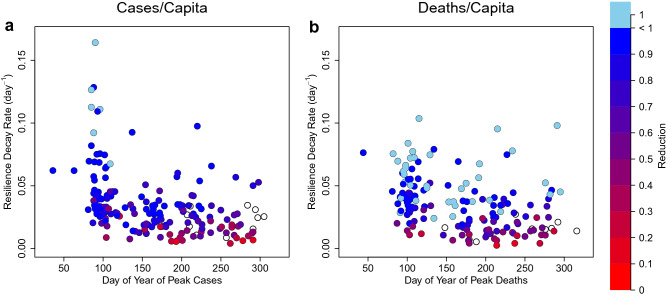
Country-level relationships between timing (day of year) of peak, resilience to COVID-19, and resulting reduction of cases and deaths. (**a**) cases/capita: relationships between day of year of peak cases and resilience (n = 176, $\rho$ =−0.51, p < 0.0001) and between day of year of peak cases and reduction (n = 164, $\rho$ =−0.54, p < 0.0001). (**b**) deaths/capita: relationships between day of year of peak deaths and resilience (n = 157, $\rho$ =−0.43, p < 0.0001) and between day of year of peak deaths and reduction (n = 150, $\rho$ =−0.39, p < 0.0001). Cases of complete reduction—i.e. elimination of cases or deaths—are denoted with pale blue. Cases where reduction is incomplete at the end of the time series are denoted with open circles.

One might expect countries experiencing waves of infections and deaths earlier in the COVID-19 pandemic to have shown less resilience, due to being caught off-guard and having less collective knowledge about how to combat the spread of infections and reduce deaths. However, several of the countries hit earliest were ones with prior experience of the SARS-CoV-1 outbreak. We find negative correlations between timing (day of year) of peak cases/capita and resilience or reduction of cases/capita, and between timing of peak deaths/capita and resilience or reduction of deaths/capita (Table 1, Fig. 4)—i.e. those hit later tended to recover slower and less completely. Potential reasons for this are examined further below. One might also expect countries in closer spatial proximity could negatively influence one another’s resilience, e.g. through cross-border movement of infected individuals. However, long-distance international travel also clearly spread the virus early on, and subsequent restrictions on travel between countries should have reduced causal interactions. Variograms of distance between countries and difference in cases/capita resilience or deaths/capita resilience, show no evidence for spatial autocorrelation of resilience (Supplementary Fig. 3).

### Wealth and public health are only weakly correlated with resilience

Demographic and public-health-related factors may be expected to influence country-level resilience, given that some significantly influence spread of the infection in within-country analysis^10^. However, no particularly strong controls emerge (Table 1). There are weak negative correlations between population and resilience of cases/capita or deaths/capita, and between country area and resilience of cases/capita or deaths/capita, but these counteract, leaving no significant effect of population density (Table 1). Richer populations (GDP/capita) tend to have greater median age (n = 175, $\rho$ =0.84, p < 0.0001), life expectancy (n = 179, $\rho$ =0.85, p < 0.0001), human development index (HDI) (n = 178, $\rho$ =0.96, p < 0.0001), and hospital beds (per 1000) (n = 161, $\rho$ =0.61, p < 0.0001). This leads to shared significant weak positive correlations of GDP/capita, median age, life expectancy, HDI, and hospital beds (per 1000) with resilience of cases/capita (Table 1). However, only hospital beds (per 1000) show a significant weak positive correlation with resilience of deaths/capita, and none of these factors significantly correlate with reduction of cases/capita or deaths/capita (Table 1).

### Adaptive changes in stringency are positively correlated with resilience

**Figure 5:**
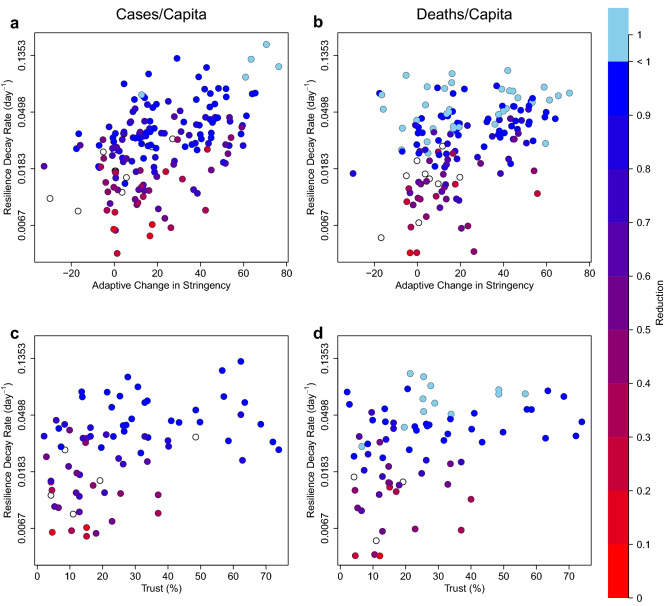
Country-level relationships between adaptive stringency or trust, resilience to COVID-19 and resulting reduction of cases and deaths. (**a**) cases/capita: relationships between adaptive stringency and resilience (n = 167, $\rho$ =0.47, p < 0.0001) and between adaptive stringency and reduction (n = 156, $\rho$ =0.29, p < 0.001). (**b**) deaths/capita: relationships between adaptive stringency and resilience (n = 153, $\rho$ =0.39, p < 0.0001) and between adaptive stringency and reduction (n = 144, $\rho$ =0.21, p < 0.05). (**c**) cases/capita: relationship between trust and resilience (n = 77, $\rho$ =0.43, p < 0.0001) and between trust and reduction (n = 72, $\rho$ =0.51, p < 0.0001). (**d**) deaths/capita: relationship between trust and resilience (n = 75, $\rho$ =0.40, p < 0.001) and between trust and reduction (n = 72, $\rho$ =0.48, p < 0.0001). Note the threshold effect whereby trust > 40% (of population agreeing with the statement “most people can be trusted”) ensures resilience of cases/capita > 0.02 d^−1^ and deaths/capita > 0.03d^−1^, which in turn support successful reduction of cases and deaths. Cases of complete reduction—i.e. elimination of cases or deaths—are denoted with pale blue. Cases where reduction is incomplete at the end of the timeseries are denoted with open circles. The trust-reduction relationships are further analysed in Supplementary Fig. 4.

Deliberate government interventions to limit social contact and thus *R*_e_ are expected^9^ to result in greater resilience (faster decay of cases and deaths). However, when looking for relationships with the OxCGRT ‘stringency index’^24,25^, we only found a weak positive relationship between decay stringency (averaged over the fitted cases/capita decay intervals) and resilience (rate of decay) of cases/capita, no relationship for deaths/capita, and weak negative relationships with reduction of cases/capita and deaths/capita (Table 1). These effects are weak because most countries maintained a similar, near maximum stringency whilst cases and deaths were being brought down, yet they exhibited very differing resilience (recovery rates). Mean stringency (averaged across the whole time series) is significantly negatively correlated with resilience of cases/capita and especially deaths/capita (Table 1). Background stringency (averaged over the intervals when decay is not occurring) is significantly and more strongly negatively correlated with resilience of cases/capita and deaths/capita, and especially with reduction of cases/capita and deaths/capita (Table 1). Only adaptive stringency (the change in stringency from before to during decay intervals) has a significant positive correlation with resilience of cases/capita and deaths/capita, with significant but weaker positive correlations to reduction of cases/capita and deaths/capita (Table 1; Fig. 5a,b). Thus, deploying stringent measures decisively when an epidemic wave erupts is beneficial. However, governments that maintain greater background and overall (mean) stringency tend to have slower recovery and tend to be less effective at reducing cases and deaths.

### Trust is positively correlated with resilience

Trust is significantly positively correlated with resilience of cases/capita and deaths/capita and especially with reduction of cases/capita and deaths/capita (Table 1; Fig. 5c,d). There is a clear threshold effect whereby all countries with trust > 40% have sufficient resilience to end in a large or complete reduction of cases and deaths (Fig. 5c,d; Supplementary Fig. 4a, b). Reduction distributions for trust ≤ 40% and trust > 40% are significantly different (cases/capita Mann–Whitney p < 0.001; deaths/capita Mann–Whitney p < 0.0001; Supplementary Fig. 4c,d). Trust and adaptive stringency are not significantly correlated, and in each case when controlling for one of the variables the resilience residuals remain strongly positively correlated with the other variable. (For cases/capita resilience, the adaptive stringency correlation $\rho$ goes from 0.428 to 0.449 and the trust correlation $\rho$ goes from 0.432 to 0.472. For deaths/capita resilience, the adaptive stringency correlation $\rho$ goes from 0.519 to 0.549 and the trust correlation $\rho$ goes from 0.398 to 0.388.) Trust is negatively correlated with mean stringency (n = 71, $\rho$ =−0.44, p < 0.001) and background stringency (n = 67, $\rho$ =−0.47, p < 0.0001), which may help explain why governments with greater background stringency are less effective at reducing COVID-19 cases and deaths—because they tend to reflect less trusting societies. Trust supports economic growth^29^ and hence has a well-known^30^ positive correlation with GDP/capita (n = 72, $\rho$ =0.70, p < 0.0001), which leads to positive correlations of trust with median age (n = 73, $\rho$ =0.55, p < 0.0001), life expectancy (n = 74, $\rho$ =0.57, p < 0.0001), HDI (n = 73, $\rho$ =0.69, p < 0.0001), and hospital beds (per 1000) (n = 71, $\rho$ =0.45, p < 0.0001). Pairwise correlation results suggest that trust exerts a stronger control than any of these factors on resilience or reduction (Table 1), but this could be influenced by the smaller sample of countries with trust data.

### Linear models confirm trust and adaptive stringency both contribute to resilience

**Figure 6:**
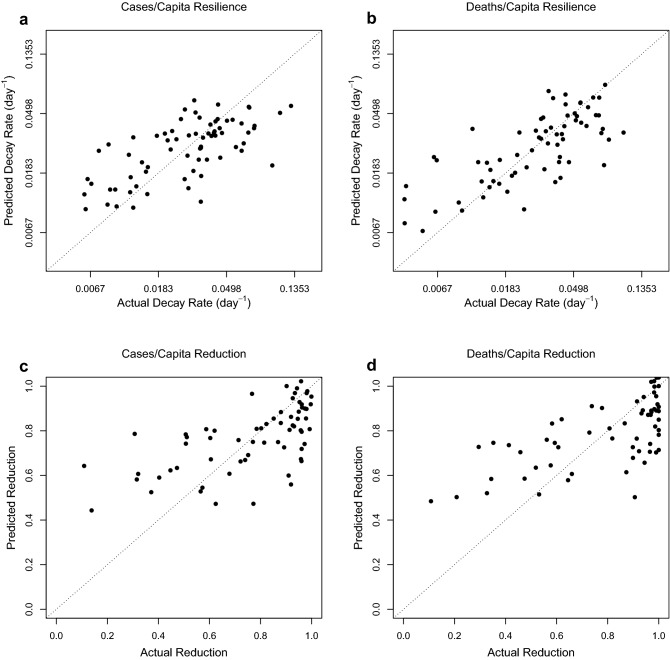
Optimised multiple linear regression models. (**a**) ln(resilience cases/capita) (n = 71, r^2^ = 0.409; Supplementary Table 4). (**b**) ln(resilience deaths/capita) (n = 69, r^2^ = 0.508; Supplementary Table 5). (**c**) cases/capita reduction (n = 66, r^2^ = 0.352; Supplementary Table 6). (**d**) deaths/capita reduction (n = 66, r^2^ = 0.414; Supplementary Table 7).

To examine this further we built various multiple linear regression models for resilience and reduction, with different mixes of social and demographic factors. Trust and adaptive stringency are consistently retained as the most significant beneficial factors. A model for resilience of cases/capita considering trust, adaptive stringency, GDP/capita, population, and hospital beds, retains adaptive stringency and trust as the most significant beneficial factors, followed by hospital beds, and rejects GDP/capita (Fig. 6a, Supplementary Table 4). A model for resilience of deaths/capita considering the same factors retains adaptive stringency and trust as the most significant beneficial factors, and GDP/capita as detrimental (Fig. 6b, Supplementary Table 5). A model for reduction of cases/capita retains trust and adaptive stringency and trust as the most significant beneficial factors, followed by hospital beds, with GDP/capita as detrimental (Fig. 6c, Supplementary Table 6). A model for reduction of deaths/capita retains adaptive stringency and trust as the most significant beneficial factors (Fig. 6d, Supplementary Table 7). If decay stringency and background stringency are used in place of adaptive stringency, they tend to be retained with significant but opposing effects, but less variance is explained, despite the extra factor (compare Supplementary Tables 8–9 with Supplementary Tables 4–5). These results confirm that trust and adaptive stringency are beneficial to resilience and reduction of both cases/capita and deaths/capita. They also suggest that trust gives rise to the significant pairwise positive correlation of GDP/capita and cases/capita resilience (Table 1) rather than vice versa.

### Confidence in politics and government are not correlated with resilience

We examined whether resilience correlates with confidence in organisations pertinent to the social contract. Trust is positively correlated with confidence in politicians (n = 74, $\rho$ =0.45, p < 0.0001), parliament (n = 74, $\rho$ =0.49, p < 0.0001), government (n = 72, $\rho$ =0.34, p < 0.01), and elections (n = 44, $\rho$ =0.39, p < 0.01). However, there are no significant (p < 0.05) correlations between confidence in any of these organisations and resilience of cases/capita or deaths/capita. Only confidence in parliament has a weak positive correlation with reduction of cases/capita (n = 72, $\rho$ =0.24, p < 0.05). Hence, we did not consider these factors further in linear models or include them in Table 1.

### Trust has more significant effects than any of Hofstede’s cultural dimensions

We also considered whether resilience correlates with any of Hofstede’s six cultural dimensions^14^ of power distance, individualism, uncertainty avoidance, masculinity, long-term orientation, and indulgence (defined above). Power distance (expectation from the less powerful that power is distributed unequally) is anti-correlated with trust (n = 49, $\rho$ =−0.70, p < 0.0001) and, consistent with that, anti-correlated with resilience of cases/capita and deaths/capita (Table 1). Individualism is positively correlated with trust (n = 49, $\rho$ =0.59, p < 0.0001) and less strongly with resilience of cases/capita, and deaths/capita (Table 1). Long-term orientation (pragmatism and preparation for the future) is positively correlated with trust (n = 63, $\rho$ =0.34, p < 0.0001) and resilience of cases/capita but not deaths/capita (Table 1). Uncertainty avoidance is negatively correlated with trust (n = 49, $\rho$ =−0.43, p < 0.01) and resilience of deaths/capita but not cases/capita (Table 1). Masculinity and indulgence do not show significant pairwise correlations with trust or resilience. Including Hofstede’s six cultural dimensions in place of trust in multiple linear regression models allows us to analyse a larger set of countries but explains less of their variance (compare Supplementary Tables 10–13 with Supplementary Tables 4–7). Mixing trust and the Hofstede dimensions in the models always retains trust as more significant than any of the retained Hofstede dimensions (Supplementary Tables 14–17).

### Temporal pattern of the pandemic

**Figure 7:**
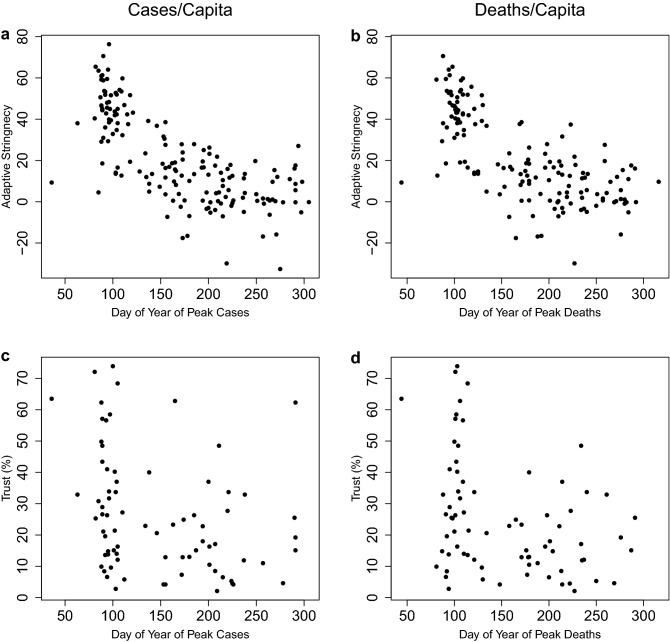
Relationships between peak timing, adaptive stringency, and trust: (**a**) day of year of peak cases/capita versus adaptive stringency (n = 166, $\rho$ =−0.74, p < 0.0001). (**b**) day of year of peak deaths/capita versus adaptive stringency (n = 154, $\rho$ =−0.72, p < 0.0001). (**c**) day of year of peak cases/capita versus trust (n = 77, $\rho$ =−0.37, p < 0.001). (**d**) day of year of peak deaths/capita versus trust (n = 75, $\rho$ =−0.30, p < 0.01).

We now return to interpreting the marked decline in resilience over time as the pandemic spread to new countries (Fig. 4). Adaptive stringency tended to decline with time; it is strongly negatively correlated with day of year of peak cases/capita (Fig. 7a) and day of year of peak deaths/capita (Fig. 7b). This is largely because background stringency increased as the pandemic progressed; it is positively correlated with day of year of peak cases/capita (n = 167, $\rho$ =0.44, p < 0.0001) and of peak deaths/capita (n = 156, $\rho$ =0.39, p < 0.0001), and secondarily because decay stringency has a weak anticorrelation with day of year of peak cases/capita (n = 166, $\rho$ =−0.16, p < 0.05), and day of year of peak deaths/capita (n = 154, $\rho$ =−0.17, p < 0.05). As the pandemic progressed it also tended to move to less trusting populations, in poorer countries, with worse healthcare. Trust is negatively correlated with day of year of peak cases/capita (Fig. 7c) and peak deaths/capita (Fig. 7d). GDP/capita is anti-correlated with day of year of peak cases/capita (n = 170, $\rho$ =−0.30, p < 0.0001) and peak deaths/capita (n = 154, $\rho$ =−0.34, p < 0.0001). Hospital beds (per 1000) are anti-correlated with day of year of peak cases/capita (n = 156, $\rho$ =−0.35, p < 0.0001) and peak deaths/capita (n = 143, $\rho$ =−0.30, p < 0.001).

These trends of declining adaptive stringency with time, and moving to less trusting populations, in poorer countries, with worse healthcare over time, could explain the decline in resilience over time (Fig. 4). To examine this further, we added day of year of peak as an extra factor considered in the linear models. Adding day of year of peak cases/capita to our model for resilience of cases/capita (Fig. 6a, Supplementary Table 4), it is retained whereas adaptive stringency is rejected (n = 71, r^2^ = 0.436; Supplementary Table 18, Supplementary Fig. 5a). In contrast, adding day of year of peak deaths/capita to our model for resilience of deaths/capita (Fig. 6b, Supplementary Table 5), retains both with adaptive stringency more significant (n = 67, r^2^ = 0.562; Supplementary Table 19, Supplementary Fig. 5b). Adding day of year of peak cases/capita to our model for reduction of cases/capita (Fig. 6c, Supplementary Table 6), it is retained whereas adaptive stringency is rejected (n = 66, r^2^ = 0.445; Supplementary Table 20, Supplementary Fig. 5c). Adding day of year of peak deaths/capita to our model for reduction of deaths/capita (Fig. 6d, Supplementary Table 7), it is rejected leaving the model unaltered. If we compare the residuals of the original models (Supplementary Tables 4–7) to the relevant day of year of peak we find no significant (p < 0.05) correlations, suggesting that we are not missing a significant additional factor correlated with day of year. Rather, the anti-correlation of day of year with adaptive stringency, trust, and other factors in the models can sometimes replace them or reduce their significance. Hence the marked declines in resilience and reduction over time (Fig. 4) are variously linked to trends of declining adaptive stringency over time (Fig. 7a,b) and the pandemic tending to progress to countries with lower trust (Fig. 7c,d), GDP/capita, and hospital beds.

## Discussion

Our results support suggestions^11,12^ that variation in resilience to COVID-19 reflects, among other things, variation in the nature and strength of the ‘social contract’ across countries. Different theories of the social contract^31^ emphasise reciprocal trust among citizens^32^, and/or between citizens and their government (political elite)^33,34^. In the latter relationship, the individual surrenders some of their freedoms and submits to an authority in return for protection of their remaining rights, usually including the right to protection of life, and of a minimum standard of health^35^. Resilience to the COVID-19 pandemic depends on the reciprocal action of governments and citizens in that governments instigate e.g. social distancing measures and citizens comply (or not) with those measures. Resilience also depends on reciprocal trust between citizens in e.g. the social contract of wearing masks^36^. Correspondingly, we find that resilience depends on both the adaptive increase in stringency of government interventions when waves of infection occur, and generalised trust within society.

The effect of stringent government interventions on resilience is not as straightforward as might be expected. Given that governments often deployed a similar maximum level of stringency when bringing down new cases and deaths, the effect of adaptive increases in stringency depends crucially on ‘background’ stringency when waves are not being brought down. Maintaining high background stringency tends to decrease resilience, tends to be associated with less trusting societies, and tends to increase over time. We speculate that maintaining high background stringency may lead to a general lack of compliance—people get tired of complying with stringent measures especially when the threat appears less acute. There are possible issues with the intervals we average stringency over. In particular, ‘background’ stringency includes sub-intervals when cases are rising and stringency is typically increased to bring about a peak. However, we also explored correlations with different measures of stringency based on e.g. minima before and maxima after peaks, and the key results (not shown) proved qualitatively robust.

More trusting societies tend to bring down cases and deaths faster (resilience) and carry on with containment efforts more effectively until the full benefits are realised (reduction). Within our dataset, a threshold level of > 40% interpersonal trust in society, seems to ensure sufficient resilience to COVID-19 to result in a near complete reduction of new cases and deaths. That said, many less-trusting societies also achieve high resilience and reduction, so trust is not the only factor that can support high resilience. We find no evidence that resilience to COVID-19 depends on confidence in specific political or governmental organisations. This is consistent with results^37^ (including our own) showing that generalised trust and confidence in political and governmental organisations are only modestly positively correlated. Several authors argue trust in state institutions influences interpersonal trust (and not vice versa)^38,39^. Still, we only find evidence for the hypothesis that resilience to COVID-19 depends on the strength of a social contract founded on reciprocal trust among citizens.

Our results add to existing evidence that trust has generally been beneficial in tackling the pandemic. Trust in government, science and medical professionals can support increased COVID-19 risk perception^40^. Trust in science^41,42^, government^41,43^ and fellow citizens^41^ positively correlate with behavioural intentions and/or reported actions to comply with COVID-19 prevention guidelines. European regions with greater trust in government reduced mobility more in response to lockdown announcements slowing the growth rate of deaths^44^, and across 25 European countries, institutional trust was associated with decreased mortality early in the pandemic^45^. However, across 84 countries worldwide, trust correlated positively with initial growth rate of deaths, possibly because it supports more cohesive relationships and interactions^46^. Subsequently, as the risks from interpersonal contact became clear, the effect switched sign and more trusting societies tended to achieve an earlier peak of new infections^47^, followed by the faster decline in infections and deaths that we show.

There of course remains lots of unexplained variance, recognising that environmental factors such as temperature^48^, humidity^48^, and UV exposure^49^ may affect the spread of COVID-19, and that different strains of COVID-19 differ in their basic reproduction number^10^. Our aim has been to focus on social factors that citizens and/or governments may have some agency to influence. Other important social factors include income inequality which is positively correlated with initial growth rate of deaths^46^, and cultural ‘tightness’ of social norms which tends to lower cumulative cases and deaths^50^. Cooperative social norms tend to be stronger in societies that have experienced more social and ecological threats in the past^50^. However, where COVID-19 has been accompanied by simultaneous perturbations such as Myanmar’s coup d’état^4^, typhoons in the Philippines^51^, or another contagious infection such as mucromycosis in India^52^, this has further challenged resilience.

Building trust is clearly desirable—and has many other benefits—but is a long-term project. Generalised trust is a relatively stable, persistent trait throughout the life course of individuals^53^, but it varies markedly between social democratic, liberal, and conservative social welfare regimes^54^, and highly trusting societies can be autocratic (e.g. China) as well as democratic. Generalised trust has declined periodically, particularly in some liberal welfare regimes^54^, most notably the US^55^. Furthermore, the rise of neoliberalism since the 1980s and austerity policies since the 2008/9 financial crash are argued to have eroded the social contract between citizens and government in many nations prior to the pandemic^12^. Most governments have responded to the pandemic with social protection policies to strengthen the social contract^12^, and trust in institutions has increased in the short-term^56–60^. However, pandemics can erode trust in the long-term^61^.

## Conclusion

Our results add to evidence that trust within society benefits resilience to epidemics^62–64^. We show that the country-level decay rate of daily COVID-19 cases or deaths from peak levels is positively correlated with trust within society, and we find evidence of a threshold effect whereby countries where > 40% agree “most people can be trusted” achieve a near complete reduction of cases and deaths. Trust is clearly not a substitute for a technical approach to epidemic control, including adaptive increases in stringency of government interventions, but it is a valuable complement—noting that vaccination reflects a social contract^65^. As the pandemic progressed, resilience declined, partly because adaptive stringency declined over time and because the pandemic tended to progress to countries with lower trust. Trust should also make societies more resilient to other types of unexpected disruption^66^. COVID-19 will surely not be the last.

## Supplementary Information

Supplementary Information.

## Data Availability

Results are available as a .csv file. Data and R code are available at https://github.com/caboulton/covid19resilience/.
