# Supplementary material for: Resilience of countries to COVID-19 correlated with trust
Source: Sci Rep. 2022 Jan 6;12:75. doi: 10.1038/s41598-021-03358-w (PMC8738739; doi:10.1038/s41598-021-03358-w)
Supplement: Supplementary file 1 — Supplementary Information. [file 41598_2021_3358_MOESM1_ESM.pdf]

Supplementary Information for 'Resilience of countries to COVID-19 correlated with trust'

Timothy M. Lenton[1]*, Chris A. Boulton[1], Marten Scheffer[2,3]

[1]Global Systems Institute, University of Exeter, Exeter, UK.

[2]Wageningen University, Wageningen, The Netherlands.

[3]Santa Fe Institute, Santa Fe, NM, USA.

*Corresponding author (t.m.lenton@exeter.ac.uk)

Supplementary Discussion

Results for cases/tests

Resilience of cases/tests, measured as the exponential decay rate (magnitude), ranges by a factor of ~40, from 0.10 d^{-1} (Fiji, New Zealand; most resilient) to 0.0026 d^{-1} (Indonesia; least resilient). Differences in resilience results between cases/capita and cases/tests were examined on a case-by-case basis using Our World in Data to visualise the data for testing intensity by country over time. For example: New Zealand shows a comparable decay rate for cases/capita, despite testing intensity increasing through the first wave. Iceland, which has a fast decay of cases/capita (0.13 d^{-1}) has a slower decay for cases/tests (0.085 d^{-1}), because testing intensity tended to track cases albeit with a lag. Indonesia's interval of declining cases in September-October interrupts an overall increase. Cases/tests show an earlier peak and longer, slower decline than cases/capita (0.0063 d^{-1}) thanks to fluctuating testing levels. Costa Rica which had the slowest decay rate of cases/capita (0.0041 d^{-1}) shows a poor fit of cases/tests not least because testing data ceases whilst cases/capita were declining through September-October. This discussion serves to illustrate why cases/tests does not produce an improved resilience dataset compared to cases/capita.

That said, correlation results for hypothesised factors affecting resilience are generally consistent between cases/capita (Table 1) and cases/tests (Supplementary Table 1). To summarise the results for resilience of cases/tests (Supplementary Table 1): Resilience of cases/tests is negatively correlated with day of year of peak cases/tests. Resilience of cases/tests is not significantly correlated with population size, country size, or population density. Resilience of cases/tests is positively correlated with median age, life expectancy, HDI, and hospital beds (per 1000), and weakly with GDP/capita. There is no significant relationship between decay stringency and resilience of cases/tests. Resilience of cases/tests is significantly negatively correlated with mean stringency and background stringency. Adaptive stringency is strongly positively correlated with resilience of cases/tests. Trust is positively correlated with resilience of cases/tests. There is no significant relationship between confidence in government, political parties, parliament or elections with resilience of cases/tests. Resilience of cases/tests declines with power distance and increases with long term orientation and individualism. The broad consistency of these results for resilience of cases/tests (Supplementary Table 1) with cases/capita (Table 1) reinforces the overall messages.

Correlation results for factors affecting reduction generally mirror those for resilience (Supplementary Table 1). The anti-correlation between day of year of peak and reduction is stronger for cases/tests than cases/capita. In contrast to cases/capita, significant effects of median age, life expectancy, HDI, hospital beds (per 1000), and GDP/capita carry over to reduction of cases/tests.

Supplementary Tables

Supplementary Table 1. Factors correlating with resilience (decay rate) and reduction of COVID-19 cases/tests across countries. Pairwise Spearman's rank correlations.

	**Resilience**			**Reduction**		
**Explanatory variable**	ρ	**p**	**n**	ρ	**p**	**n**
Day of year of peak	-0.59	<0.0001	105	-0.75	<0.0001	101
Population	(-0.13)	-	105	(-0.14)	-	101
Country size	(-0.19)	-	104	(-0.07)	-	100
Population density	(0.05)	-	104	(-0.11)	-	100
GDP/capita	0.24	<0.05	103	0.29	<0.01	99
Median age	0.42	<0.0001	105	0.37	<0.001	101
Life expectancy	0.47	<0.0001	105	0.37	<0.001	101
Human Develop. Index	0.38	<0.0001	104	0.35	<0.001	100
Hospital beds	0.36	<0.001	94	0.28	<0.01	90
Mean stringency	-0.39	<0.0001	100	-0.45	<0.0001	96
Decay stringency	(0.19)	-	99	(0.06)	-	95
Background stringency	-0.48	<0.0001	100	-0.53	<0.0001	96
Adaptive stringency	0.67	<0.0001	99	0.63	<0.0001	95
Trust	0.41	<0.01	55	0.44	<0.001	54
Power distance	-0.41	<0.001	77	-0.37	<0.01	74
Individualism	0.28	<0.05	77	0.33	<0.01	74
Masculinity	(0.05)	-	77	(-0.02)	-	74
Uncertainty avoidance	(-0.02)	-	77	(-0.17)	-	74
Long-term orientation	0.33	<0.01	88	(0.19)	-	84
Indulgence	(0.13)	-	87	(0.17)	-	83

Supplementary Table 2. Pairwise Spearman's rank correlations for resilience and reduction for the dataset of first peak only in each country.

	Resilience						Reduction					
	Cases/capita			Deaths/capita			Cases/capita			Deaths/capita		
**Explanatory variable**	ρ	**p**	**n**	ρ	**p**	**n**	ρ	**p**	**n**	ρ	**p**	**n**
Day of year of peak	-0.59	<0.0001	143	-0.54	<0.0001	130	-0.49	<0.0001	137	-0.44	<0.0001	123
Population	-0.29	<0.001	143	-0.32	<0.001	130	-0.31	<0.001	137	-0.28	<0.01	123
Country size	-0.30	<0.001	141	-0.24	<0.01	128	-0.23	<0.01	135	-0.21	<0.05	121
Population density	(0.10)	-	141	(0.03)	-	128	(-0.01)	-	135	(0.01)	-	121
GDP/capita	0.24	<0.01	138	(0.09)	-	126	(0.12)	-	132	(0.08)	-	119
Median age	0.27	<0.01	140	(0.16)	-	127	(0.08)	-	134	(0.02)	-	120
Life expectancy	0.30	<0.001	142	0.19	<0.05	129	(0.10)	-	136	(0.06)	-	122
Human Develop. Index	0.27	<0.01	139	(0.14)	-	127	(0.10)	-	133	(0.08)	-	120
Hospital beds	0.33	<0.001	127	0.28	<0.01	116	(0.17)	-	122	(0.13)	-	110
Mean stringency	-0.22	<0.01	136	-0.42	<0.0001	128	-0.45	<0.0001	130	-0.52	<0.0001	121
Decay stringency	0.19	<0.05	136	(0.04)	-	127	-0.21	<0.05	130	-0.25	<0.01	120
Background stringency	-0.29	<0.001	136	-0.54	<0.0001	128	-0.52	<0.0001	130	-0.59	<0.0001	121
Adaptive stringency	0.48	<0.0001	136	0.46	<0.0001	127	0.19	<0.05	130	0.22	<0.05	120
Trust	0.45	<0.001	67	0.42	<0.001	64	0.59	<0.0001	63	0.55	<0.0001	61
Power distance	-0.36	<0.001	89	-0.31	<0.01	84	-0.24	<0.05	88	-0.32	<0.01	81
Individualism	0.23	<0.05	89	0.32	<0.01	84	(0.09)	-	88	0.27	<0.05	81
Masculinity	(0.11)	-	89	(0.02)	-	84	(-0.04)	-	88	(0.0)	-	81
Uncertainty avoidance	(-0.04)	-	89	(-0.18)	-	84	-0.23	<0.05	88	-0.31	<0.01	81
Long-term orientation	0.23	<0.05	104	(0.18)	-	95	(0.06)	-	101	(0.03)	-	91
Indulgence	(0.01)	-	104	(0.02)	-	96	(0.18)	-	101	0.27	<0.01	92

Supplementary Table 3. Pairwise Spearman's rank correlations for resilience and reduction for dataset of more stringent fits of exponential decay $r^2 \geq 0.9$.

	**Resilience**						**Reduction**					
	**Cases/capita**			**Deaths/capita**			**Cases/capita**			**Deaths/capita**		
**Explanatory variable**	ρ	**p**	**n**	ρ	**p**	**n**	ρ	**p**	**n**	ρ	**p**	**n**
Day of year of peak	-0.49	<0.0001	138	-0.46	<0.0001	119	-0.53	<0.0001	128	-0.40	<0.0001	111
Population	-0.28	<0.001	139	-0.29	<0.01	120	-0.22	<0.05	129	-0.21	<0.05	112
Country size	-0.33	<0.0001	137	-0.20	<0.05	120	-0.19	<0.05	127	(-0.12)	-	112
Population density	(0.14)	-	137	(0.02)	-	120	(0.02)	-	127	(-0.06)	-	112
GDP/capita	0.20	<0.05	133	(0.06)	-	117	(0.08)	-	123	(0.10)	-	109
Median age	0.30	<0.001	134	(0.15)	-	117	(0.09)	-	124	(0.04)	-	109
Life expectancy	0.28	<0.001	137	(0.14)	-	119	(0.08)	-	127	(0.03)	-	111
Human Develop. Index	0.26	<0.01	134	(0.11)	-	117	(0.10)	-	124	(0.09)	-	109
Hospital beds	0.32	<0.001	122	0.30	<0.01	110	(0.14)	-	113	(0.19)	-	103
Mean stringency	-0.22	<0.01	132	-0.48	<0.0001	116	-0.42	<0.0001	123	-0.56	<0.0001	108
Decay stringency	0.18	<0.05	132	(0.01)	-	114	(-0.17)	-	123	-0.29	<0.01	106
Background stringency	-0.31	<0.001	132	-0.55	<0.0001	116	-0.49	<0.0001	123	-0.61	<0.0001	108
Adaptive stringency	0.49	<0.0001	132	0.45	<0.0001	114	0.28	<0.01	123	0.24	<0.05	106
Trust	0.47	<0.001	55	0.40	<0.01	58	0.48	<0.001	51	0.55	<0.0001	55
Power distance	-0.37	<0.001	87	(-0.22)	-	77	-0.25	<0.05	84	-0.24	<0.05	73
Individualism	(0.17)	-	87	0.26	<0.05	77	(0.06)	-	84	0.25	<0.05	73
Masculinity	(-0.06)	-	87	(-0.02)	-	77	(-0.17)	-	84	(0.01)	-	73
Uncertainty avoidance	(0.03)	-	87	(-0.18)	-	77	(-0.15)	-	84	-0.26	<0.05	73
Long-term orientation	0.28	<0.01	104	0.22	<0.05	87	(0.07)	-	98	(0.12)	-	81
Indulgence	(0.0)	-	103	(-0.01)	-	86	(0.16)	-	97	0.25	<0.05	80

Supplementary Table 4. Optimised multiple linear regression model for ln(resilience cases/capita) considering trust, adaptive stringency, GDP/capita, population, and hospital beds (n=71, r^2=0.409).

Factor	Coefficient	SE	t	p
(Intercept)	-3.239260	0.846889	-3.825	0.000293
∆Stringency	0.012153	0.003862	3.147	0.002477
ln(Population)	-0.073385	0.045737	-1.604	0.113382
ln(Hospital beds)	0.212200	0.094458	2.246	0.028024
Trust	0.011607	0.003735	3.107	0.002783

Supplementary Table 5. Optimised multiple linear regression model for ln(resilience deaths/capita) considering trust, adaptive stringency, GDP/capita, population, and hospital beds (n=69, r^2=0.508).

Factor	Coefficient	SE	t	p
(Intercept)	0.654789	1.432163	0.457	0.64910
ln(GDP/capita)	-0.433278	0.131188	-3.303	0.00158
∆Stringency	0.021039	0.003989	5.275	1.74e-06
ln(Population)	-0.080640	0.052499	-1.536	0.12953
ln(Hospital beds)	0.211839	0.110687	1.914	0.06018
Trust	0.022932	0.004746	4.832	9.02e-06

Supplementary Table 6. Optimised multiple linear regression model for reduction of cases/capita considering trust, adaptive stringency, GDP/capita, population, and hospital beds (n=66, r^2=0.352).

Factor	Coefficient	SE	t	p
(Intercept)	1.262139	0.382539	3.299	0.00162
ln(GDP/capita)	-0.091431	0.043852	-2.085	0.04126
∆Stringency	0.004322	0.001307	3.306	0.00159
ln(Hospital beds)	0.085271	0.036418	2.341	0.02249
Trust	0.006781	0.001548	4.381	4.72e-05

Supplementary Table 7. Optimised multiple linear regression model for reduction of deaths/capita considering trust, adaptive stringency, GDP/capita, population, and hospital beds (n=66, r^2=0.414).

Factor	Coefficient	SE	t	p
(Intercept)	0.970530	0.330757	2.934	0.004682
ln(GDP/capita)	-0.052773	0.036684	-1.439	0.155305
∆Stringency	0.007198	0.001241	5.799	2.43e-07
Trust	0.005689	0.001560	3.647	0.000545

Supplementary Table 8. Optimised multiple linear regression model for ln(resilience cases/capita) considering trust, decay stringency, background stringency, GDP/capita, population, and hospital beds (n=71, r^2=0.387).

Factor	Coefficient	SE	t	p
(Intercept)	-3.358484	0.898354	-3.738	0.000393
ln(Population)	-0.073334	0.050997	-1.438	0.155234
ln(Hospital beds)	0.223738	0.100987	2.216	0.030234
Decay stringency	0.017311	0.006064	2.855	0.005774
Background stringency	-0.015737	0.008453	-1.862	0.067161
Trust	0.012210	0.003970	3.076	0.003071

Supplementary Table 9. Optimised multiple linear regression model for ln(resilience deaths/capita) considering trust, decay stringency, background stringency, GDP/capita, population, and hospital beds (n=69, r^2=0.502).

Factor	Coefficient	SE	t	p
(Intercept)	-0.225081	1.487648	-0.151	0.88023
ln(Population)	-0.092774	0.057580	-1.611	0.11221
ln(GDP/capita)	-0.367504	0.130544	-2.815	0.00653
ln(Hospital beds)	0.218632	0.117086	1.867	0.06659
Decay stringency	0.031497	0.006015	5.236	2.07×10^{-6}
Background stringency	-0.023897	0.008471	-2.821	0.00642
Trust	0.023407	0.004978	4.702	1.48×10^{-5}

Supplementary Table 10. Optimised multiple linear regression model for ln(resilience cases/capita) considering Hofstede's 6 cultural dimensions, adaptive stringency, GDP/capita, population, and hospital beds (n=88, r^2=0.382).

Factor	Coefficient	SE	t	p
(Intercept)	0.065035	0.878650	0.074	0.941182
ln(Population)	-0.151172	0.036012	-4.198	6.94×10^{-5}
ln(GDP/capita)	-0.160024	0.056346	-2.840	0.005718
∆Stringency	0.013202	0.002829	4.667	1.21×10^{-5}
Power distance	-0.006009	0.003291	-1.826	0.071628
Masculinity	0.008514	0.003378	2.520	0.013710
Uncertainty avoidance	-0.004315	0.003067	-1.407	0.163351
Long term orientation	0.009039	0.002629	3.438	0.000933

Supplementary Table 11. Optimised multiple linear regression model for ln(resilience deaths/capita) considering same factors as Supplementary Table 10 (n=83, r^2=0.376).

Factor	Coefficient	SE	t	p
(Intercept)	1.578207	1.033408	1.527	0.13092
ln(Population)	-0.183831	0.040633	-4.524	2.23×10^{-5}
ln(GDP/capita)	-0.195482	0.066158	-2.955	0.00418
∆Stringency	0.014473	0.003477	4.162	8.33×10^{-5}
Power distance	-0.007450	0.003593	-2.073	0.04158
Masculinity	0.007022	0.003768	1.864	0.06627
Uncertainty avoidance	-0.009800	0.003316	-2.955	0.00417
Long term orientation	0.006826	0.002932	2.328	0.02261

Supplementary Table 12. Optimised multiple linear regression model for reduction of cases/capita considering same factors as Supplementary Table 10 (n=84, r^2=0.292).

Factor	Coefficient	SE	t	p
(Intercept)	1.9240358	0.2863038	6.720	2.67×10^{-9}
ln(Population)	-0.0426342	0.0120621	-3.535	0.000690
ln(GDP/capita)	-0.0497983	0.0186822	-2.666	0.009339
∆Stringency	0.0036423	0.0009931	3.667	0.000446
Uncertainty avoidance	-0.0027465	0.0010402	-2.640	0.010003
Long term orientation	0.0032869	0.0009391	3.500	0.000772

Supplementary Table 13. Optimised multiple linear regression model for reduction of deaths/capita considering same factors as Supplementary Table 10 (n=79, r^2=0.374).

Factor	Coefficient	SE	t	p
(Intercept)	2.138040	0.306327	6.980	1.21×10^{-9}
ln(Population)	-0.045579	0.012150	-3.751	0.000353
ln(GDP/capita)	-0.043789	0.019633	-2.230	0.028838
∆Stringency	0.004972	0.001104	4.505	2.51×10^{-5}
Power distance	-0.002686	0.001123	-2.392	0.019391
Masculinity	0.001808	0.001164	1.553	0.124711
Uncertainty avoidance	-0.002374	0.001023	-2.319	0.023209

Supplementary Table 14. Optimised multiple linear regression model for ln(resilience cases/capita) considering trust, Hofstede's 6 cultural dimensions, adaptive stringency, GDP/capita, population, and hospital beds (n=52, r^2=0.532).

Factor	Coefficient	SE	t	p
(Intercept)	-2.257826	1.003801	-2.249	0.029435
ln(Population)	-0.132911	0.055857	-2.379	0.021637
∆Stringency	0.015180	0.004181	3.631	0.000720
Individualism	-0.009176	0.004104	-2.236	0.030358
Masculinity	0.013417	0.003759	3.569	0.000866
Indulgence	-0.008775	0.003724	-2.356	0.022887
Trust	0.024357	0.004906	4.965	1.03×10^{-5}

Supplementary Table 15. Optimised multiple linear regression model for ln(resilience deaths/capita) considering same factors as Supplementary Table 14 (n=52, r^2=0.530).

Factor	Coefficient	SE	t	p
(Intercept)	-1.990951	1.159329	-1.717	0.09295
ln(Population)	-0.134119	0.063093	-2.126	0.03918
∆Stringency	0.017690	0.004596	3.849	0.00038
Individualism	-0.007397	0.004792	-1.543	0.12988
Masculinity	0.013249	0.004533	2.923	0.00546
Long term orientation	-0.008082	0.004749	-1.702	0.09583
Indulgence	-0.012791	0.005371	-2.382	0.02163
Trust	0.030971	0.006366	4.865	1.51×10^{-5}

Supplementary Table 16. Optimised multiple linear regression model for reduction of cases/capita considering same factors as Supplementary Table 14 (n=50, r^2=0.518).

Factor	Coefficient	SE	t	p
(Intercept)	2.192309	0.582217	3.765	0.00049
ln(Population)	-0.036858	0.017365	-2.122	0.03946
ln(GDP/capita)	-0.129780	0.049491	-2.622	0.01195
∆Stringency	0.004789	0.001483	3.230	0.00234
Long term orientation	0.003280	0.001060	3.094	0.00343
Trust	0.007225	0.001590	4.543	4.29×10^{-5}

Supplementary Table 17. Optimised multiple linear regression model for reduction of deaths/capita considering same factors as Supplementary Table 14 (n=51, r^2=0.533).

Factor	Coefficient	SE	t	p
(Intercept)	2.060966	0.621679	3.315	0.00182
ln(Population)	-0.031950	0.018684	-1.710	0.09415
ln(GDP/capita)	-0.121991	0.050866	-2.398	0.02068
∆Stringency	0.006631	0.001391	4.769	1.98×10^{-5}
Masculinity	0.002239	0.001217	1.840	0.07241
Trust	0.007950	0.001669	4.764	2.01×10^{-5}

Supplementary Table 18. Optimised multiple linear regression model for ln(resilience cases/capita) considering day of year of peak cases/capita, trust, adaptive stringency, GDP/capita, population, and hospital beds (n=71, r^2=0.436).

Factor	Coefficient	SE	t	p
(Intercept)	-1.627345	0.754757	-2.156	0.034724
ln(Population)	-0.104462	0.042084	-2.482	0.015607
ln(Hospital beds)	0.181066	0.093640	1.934	0.057447
Trust	0.007958	0.003723	2.138	0.036244
Cases Peak DoY	-0.004234	0.001147	-3.692	0.000453

Supplementary Table 19. Optimised multiple linear regression model for ln(resilience deaths/capita) considering day of year of peak deaths/capita, trust, adaptive stringency, GDP/capita, population, and hospital beds (n=67, r^2=0.562).

Factor	Coefficient	SE	t	p
(Intercept)	1.573762	1.492169	1.055	0.295800
ln(GDP/capita)	-0.422638	0.129228	-3.270	0.001781
∆Stringency	0.013972	0.005434	2.571	0.012638
ln(Population)	-0.093750	0.050180	-1.868	0.066607
ln(Hospital beds)	0.212894	0.105982	2.009	0.049067
Trust	0.019697	0.004812	4.094	0.000129
Deaths Peak DoY	-0.003680	0.001774	-2.074	0.042356

Supplementary Table 20. Optimised multiple linear regression model for reduction of cases/capita considering day of year of peak cases/capita, trust, adaptive stringency, GDP/capita, population, and hospital beds (n=66, r^2=0.445).

Factor	Coefficient	SE	t	p
(Intercept)	2.254609	0.447139	5.042	4.53×10^{-6}
ln(GDP/capita)	-0.096718	0.040343	-2.397	0.019642
ln(Population)	-0.030241	0.013229	-2.286	0.025802
ln(Hospital beds)	0.058658	0.034372	1.707	0.093078
Trust	0.005223	0.001415	3.692	0.000483
Cases Peak DoY	-0.001627	0.000398	-4.088	0.000131

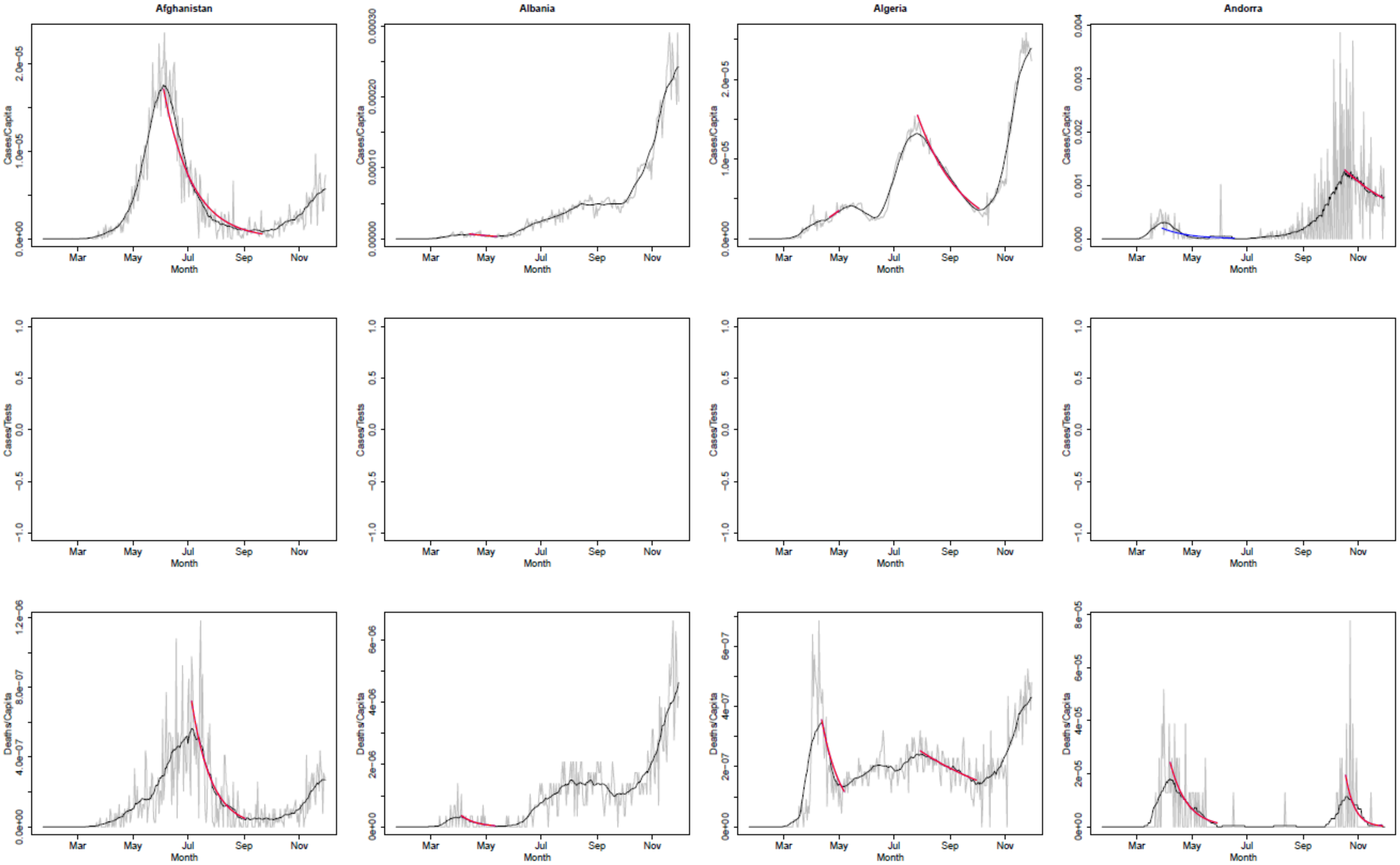

Supplementary Figure 1. Timeseries for all countries that have sufficient data for analysis (alphabetical order) showing raw data (grey), smoothed data (black), and decay interval exponential fits (red $r^2 \geq 0.8$, blue $r^2 < 0.8$). Top row cases/capita, middle row cases/tests, bottom row deaths/capita.

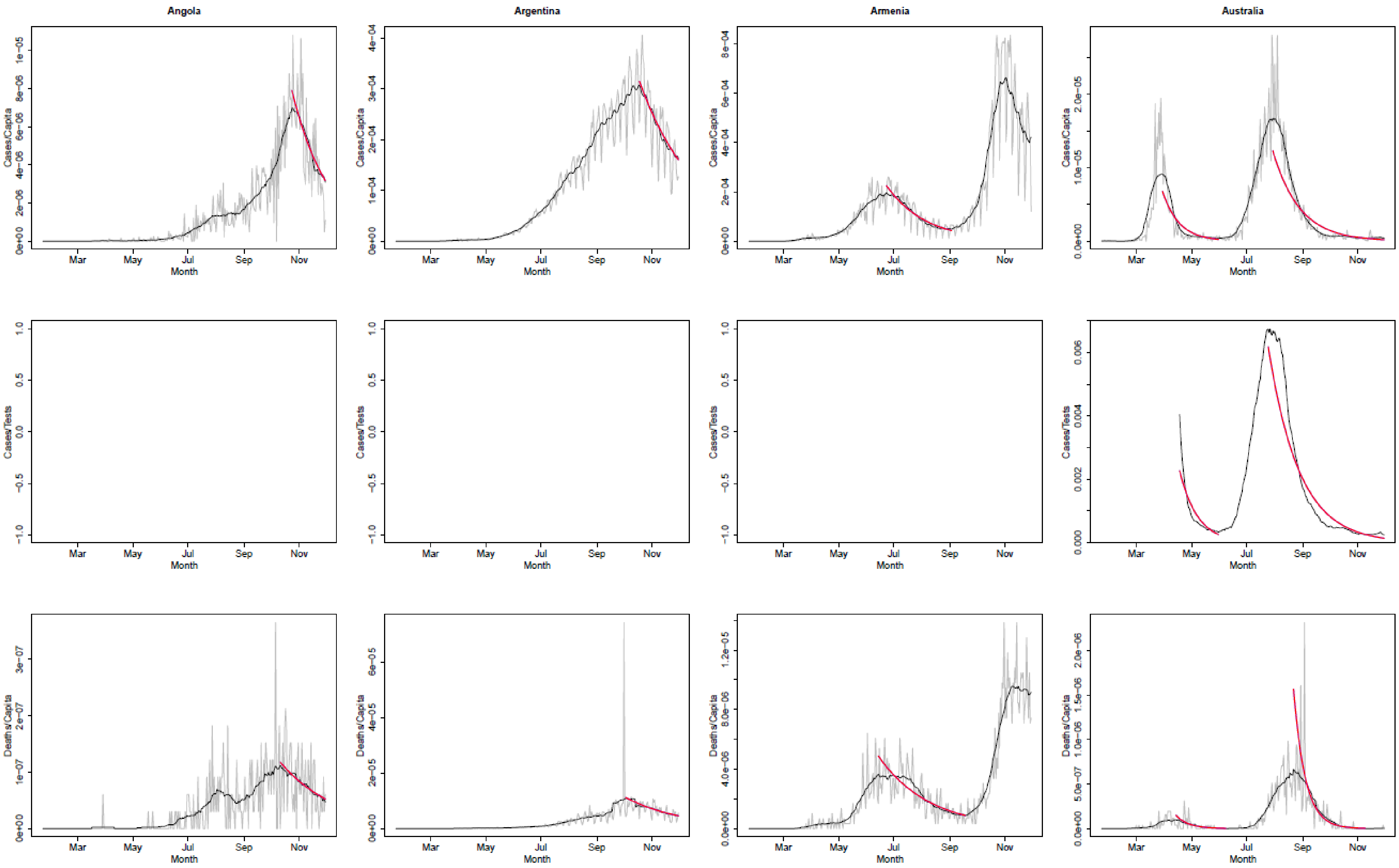
Angola
Argentina
Armenia
Australia
Cases/Capita
Cases/Tests
Deaths/Capita
Mar
May
Jul
Sep
Nov
Month

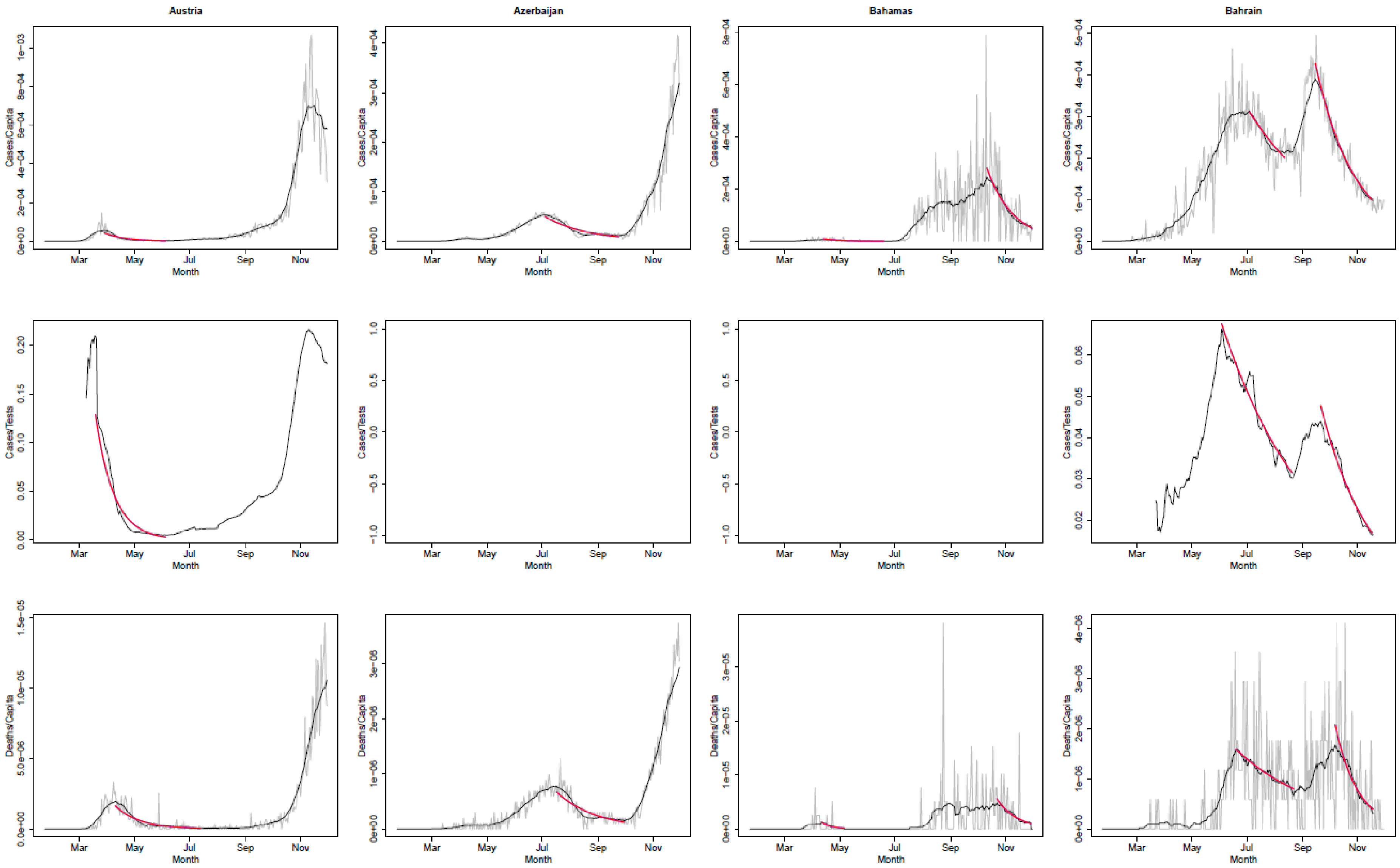
Austria
Azerbaijan
Bahamas
Bahrain
Cases/Capita
Cases/Tests
Deaths/Capita
Month
Mar
May
Jul
Sep
Nov

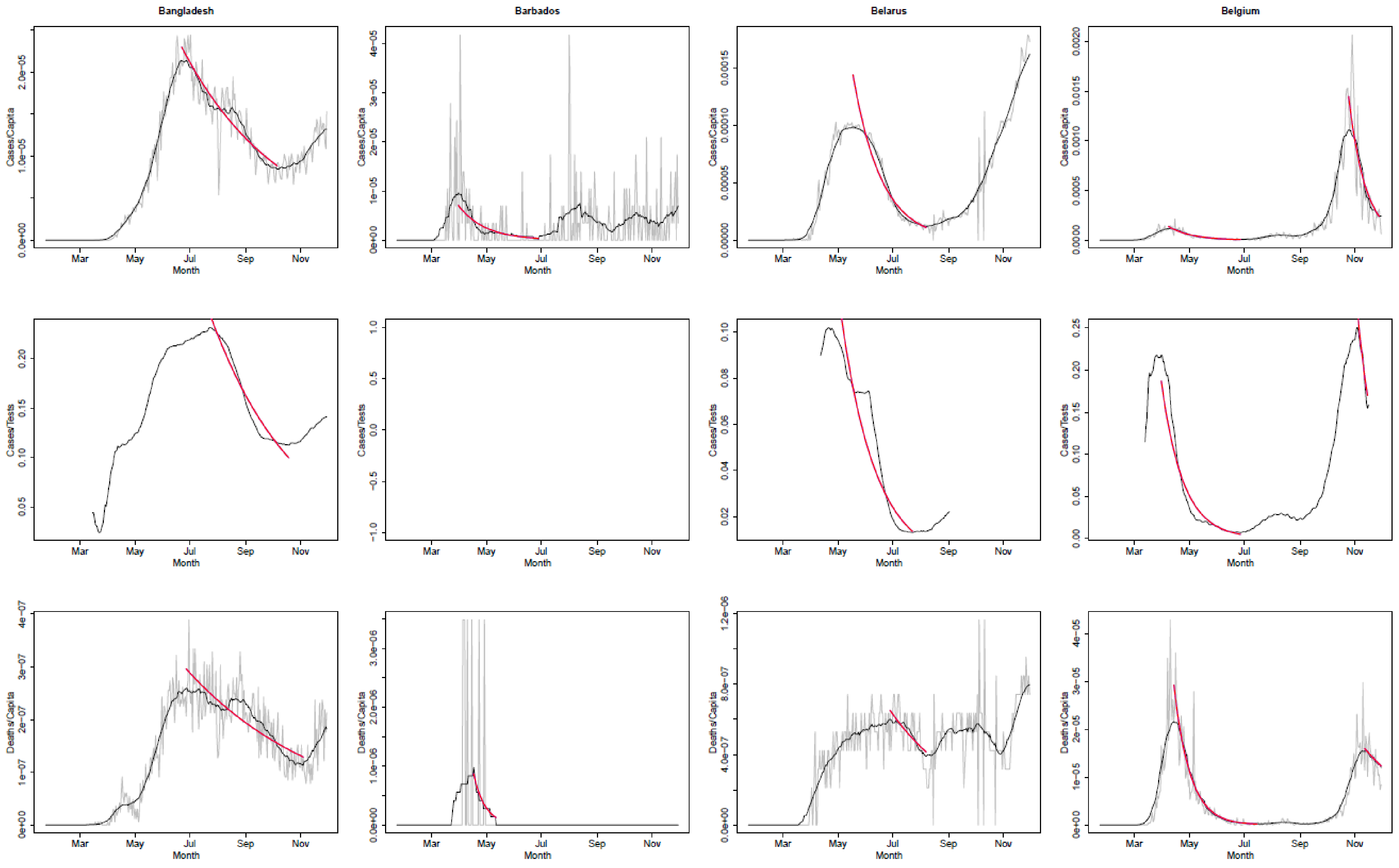
Bangladesh
Barbados
Belarus
Belgium
Cases/Capita
Cases/Tests
Deaths/Capita
Month
Mar
May
Jul
Sep
Nov

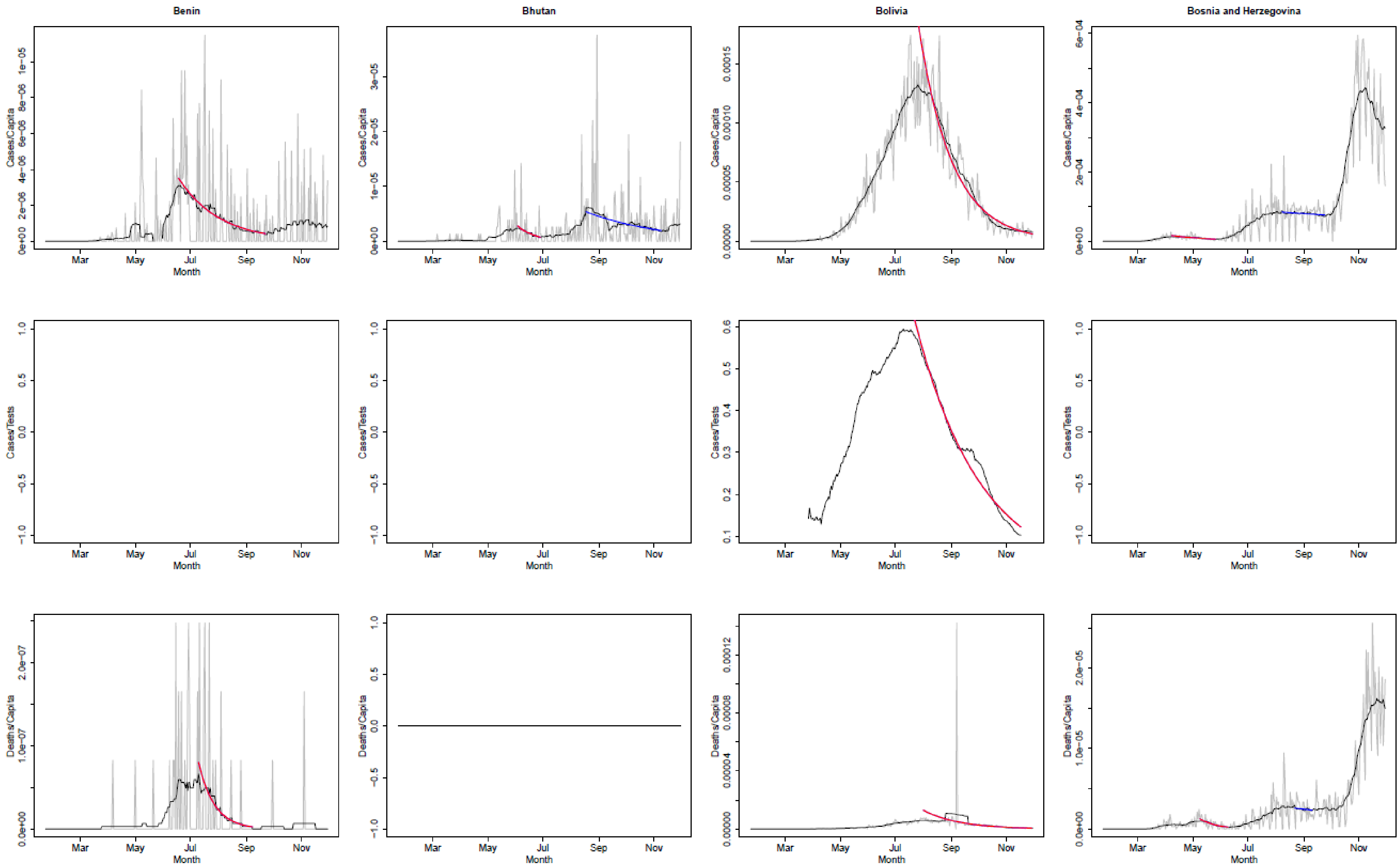

Benin
Bhutan
Bolivia
Bosnia and Herzegovina
Cases/Capita
Cases/Tests
Deaths/Capita
Mar
May
Jul
Sep
Nov
Month

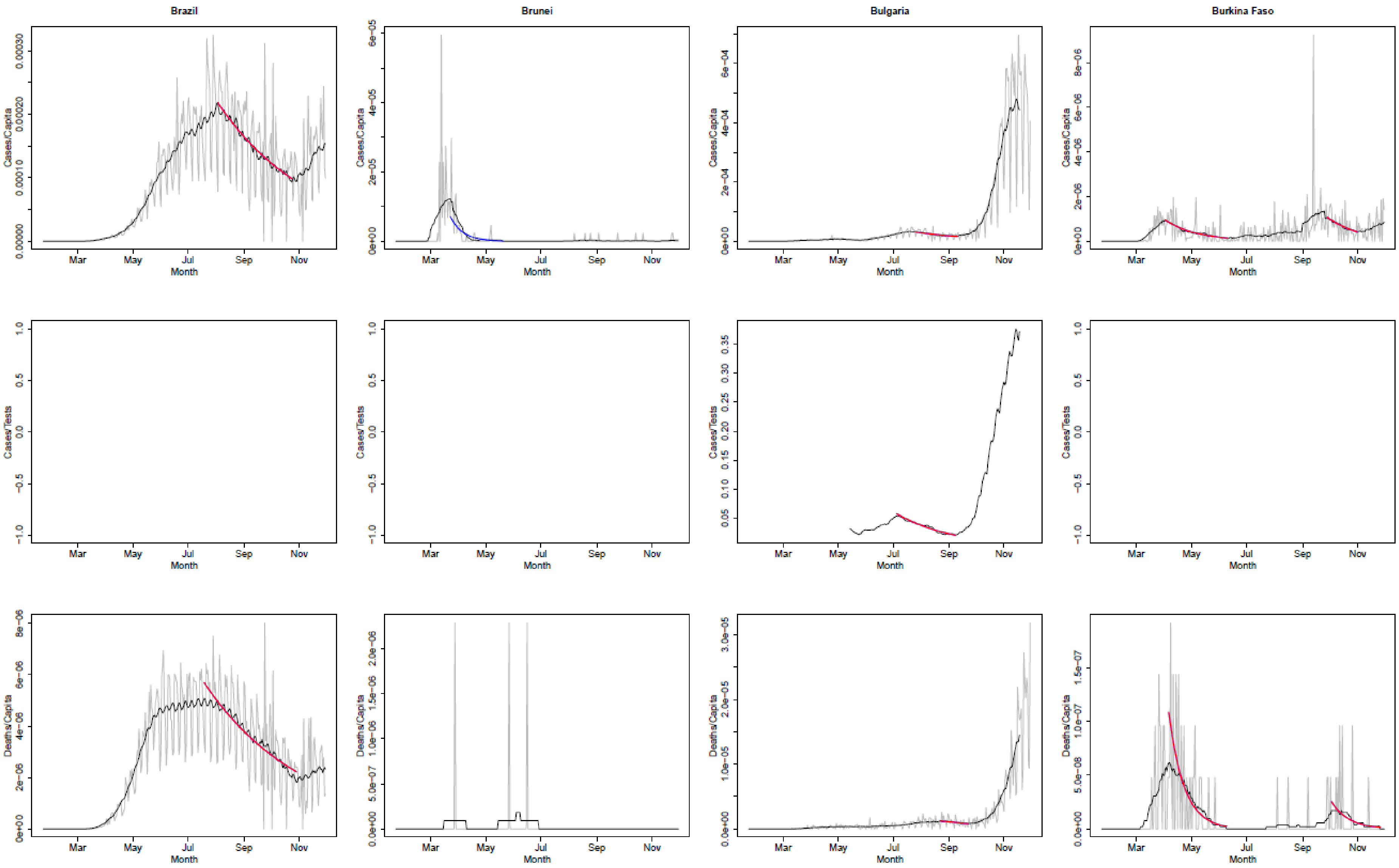
Brazil
Brunei
Bulgaria
Burkina Faso
Cases/Capita
Cases/Tests
Deaths/Capita
Mar
May
Jul
Sep
Nov
Month

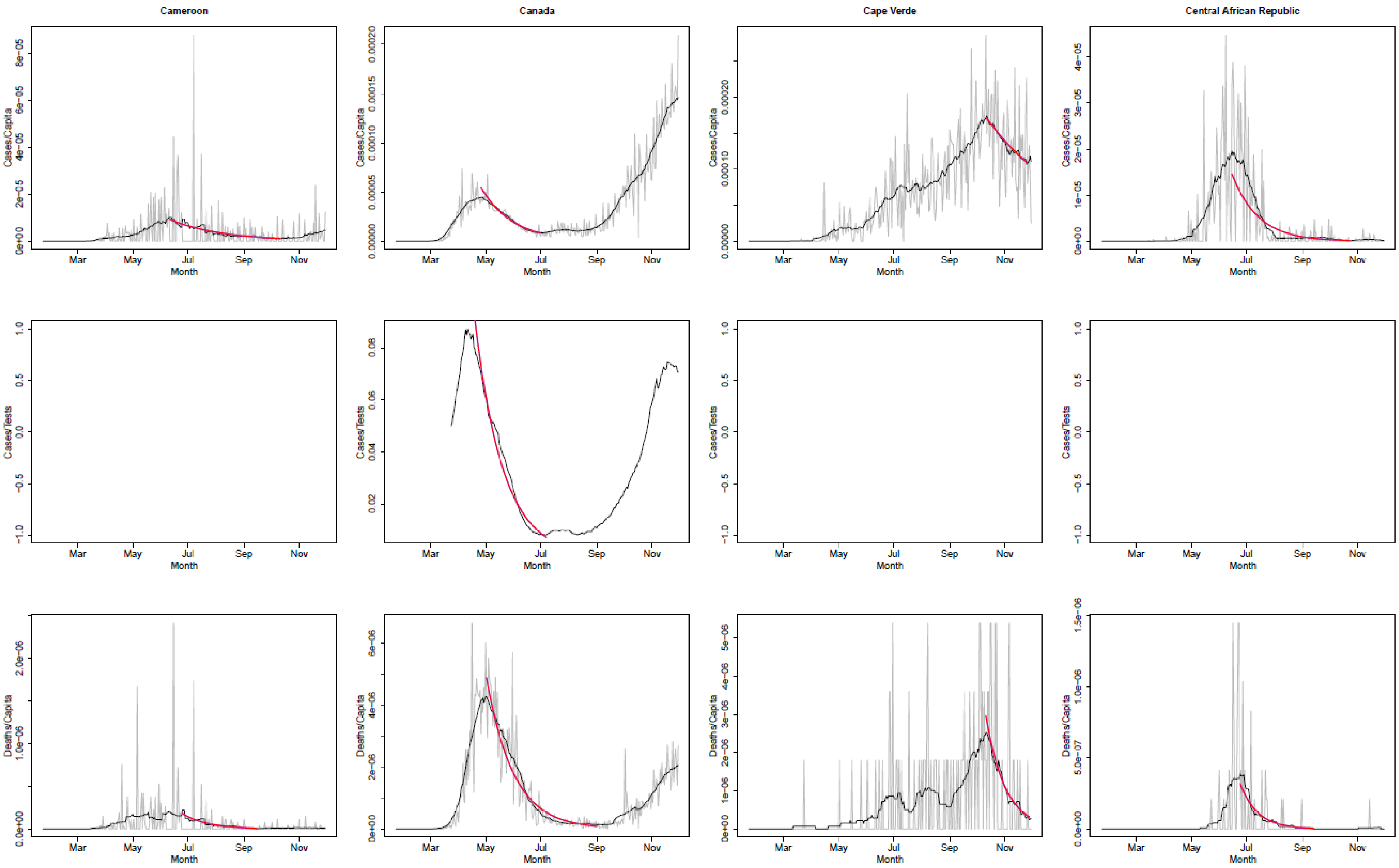
Cameroon
Canada
Cape Verde
Central African Republic
Cases/Capita
Cases/Tests
Deaths/Capita
Month
Mar
May
Jul
Sep
Nov
0e+00
2e−05
4e−05
6e−05
8e−05
0.00000
0.00005
0.00010
0.00015
0.00020
1e−05
3e−05
−1.0
−0.5
0.0
0.5
1.0
0.02
0.04
0.06
0.08
0.0e+00
1.0e−06
2.0e−06
2e−06
4e−06
6e−06
1e−06
3e−06
5e−06
5.0e−07
1.5e−06

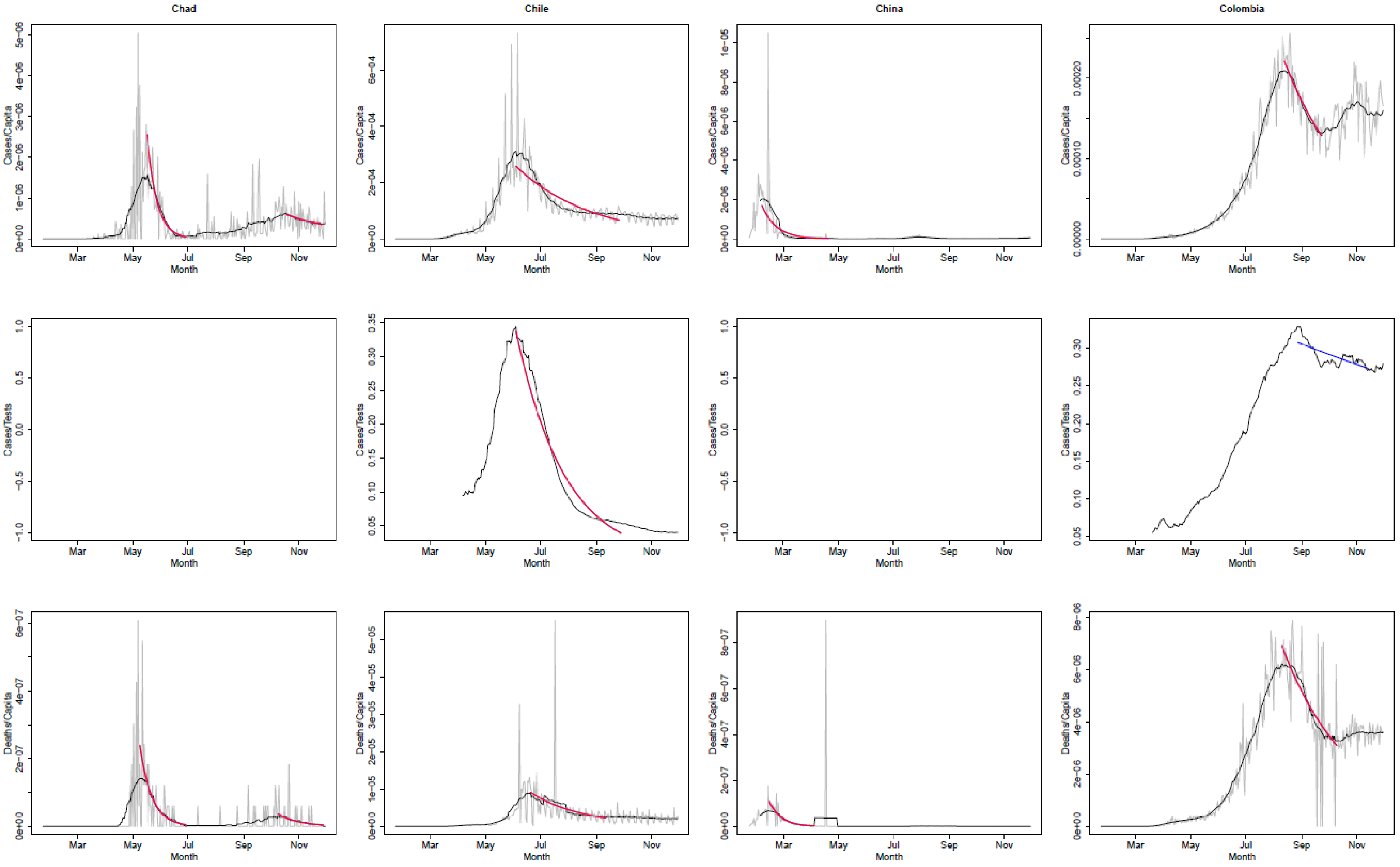
Chad
Chile
China
Colombia
Cases/Capita
Cases/Tests
Deaths/Capita
Mar
May
Jul
Sep
Nov
Month

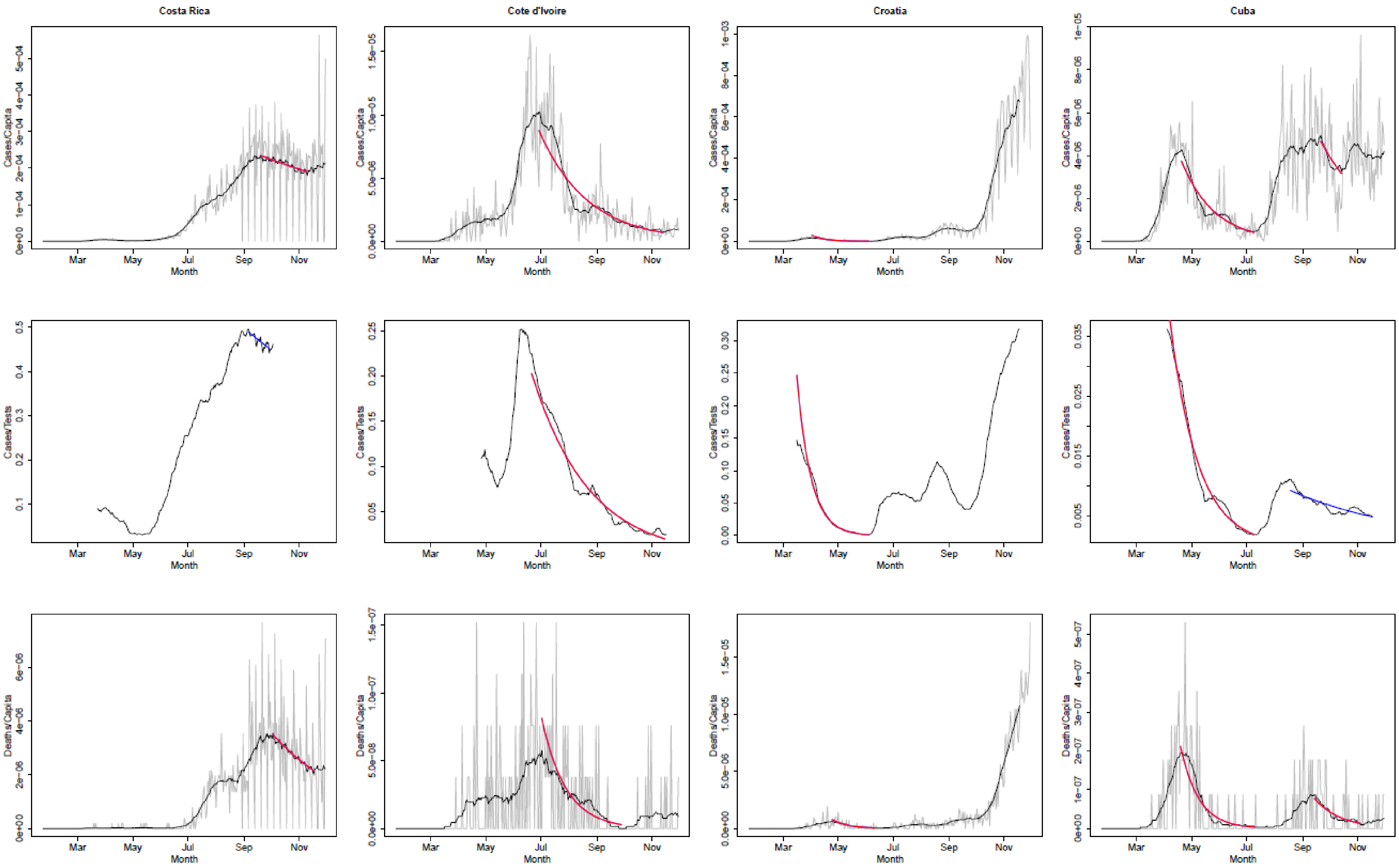
Costa Rica
Cote d'Ivoire
Croatia
Cuba
Cases/Capita
Cases/Tests
Deaths/Capita
Month
Mar
May
Jul
Sep
Nov

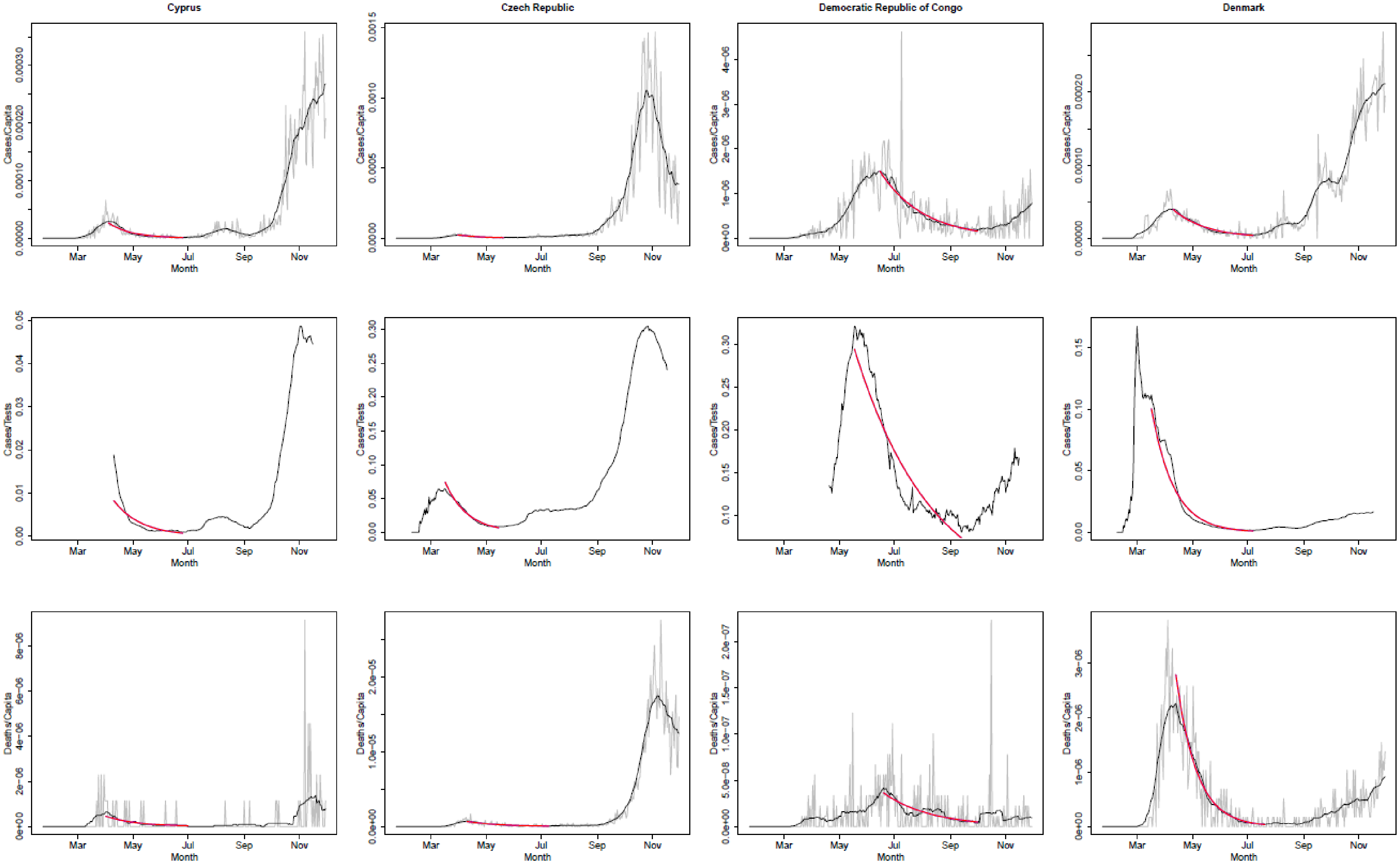
Cyprus
Czech Republic
Democratic Republic of Congo
Denmark
Cases/Capita
Cases/Tests
Deaths/Capita
Mar
May
Jul
Sep
Nov
Month

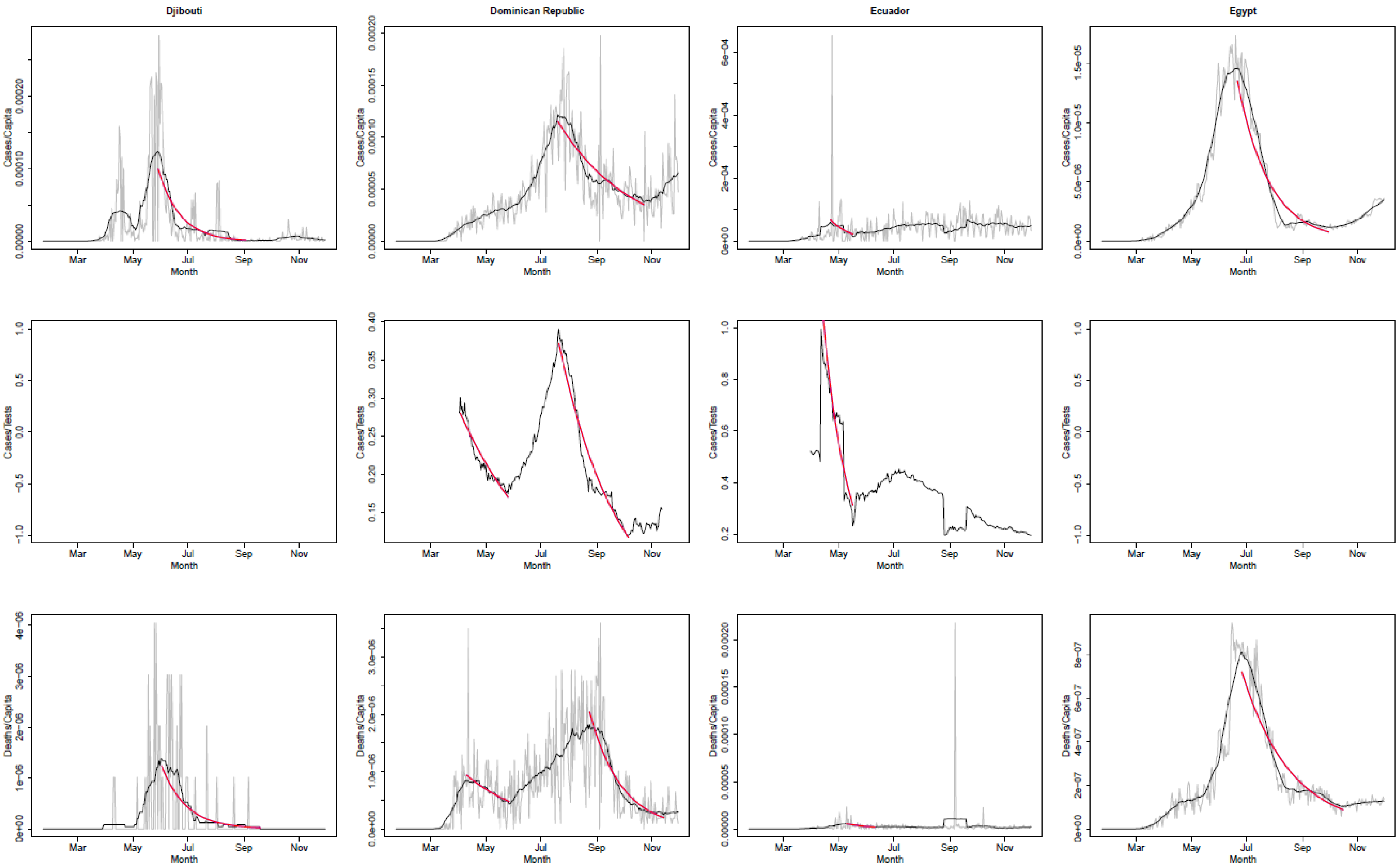
Djibouti
Dominican Republic
Ecuador
Egypt
Cases/Capita
Cases/Tests
Deaths/Capita
Mar
May
Jul
Sep
Nov
Month

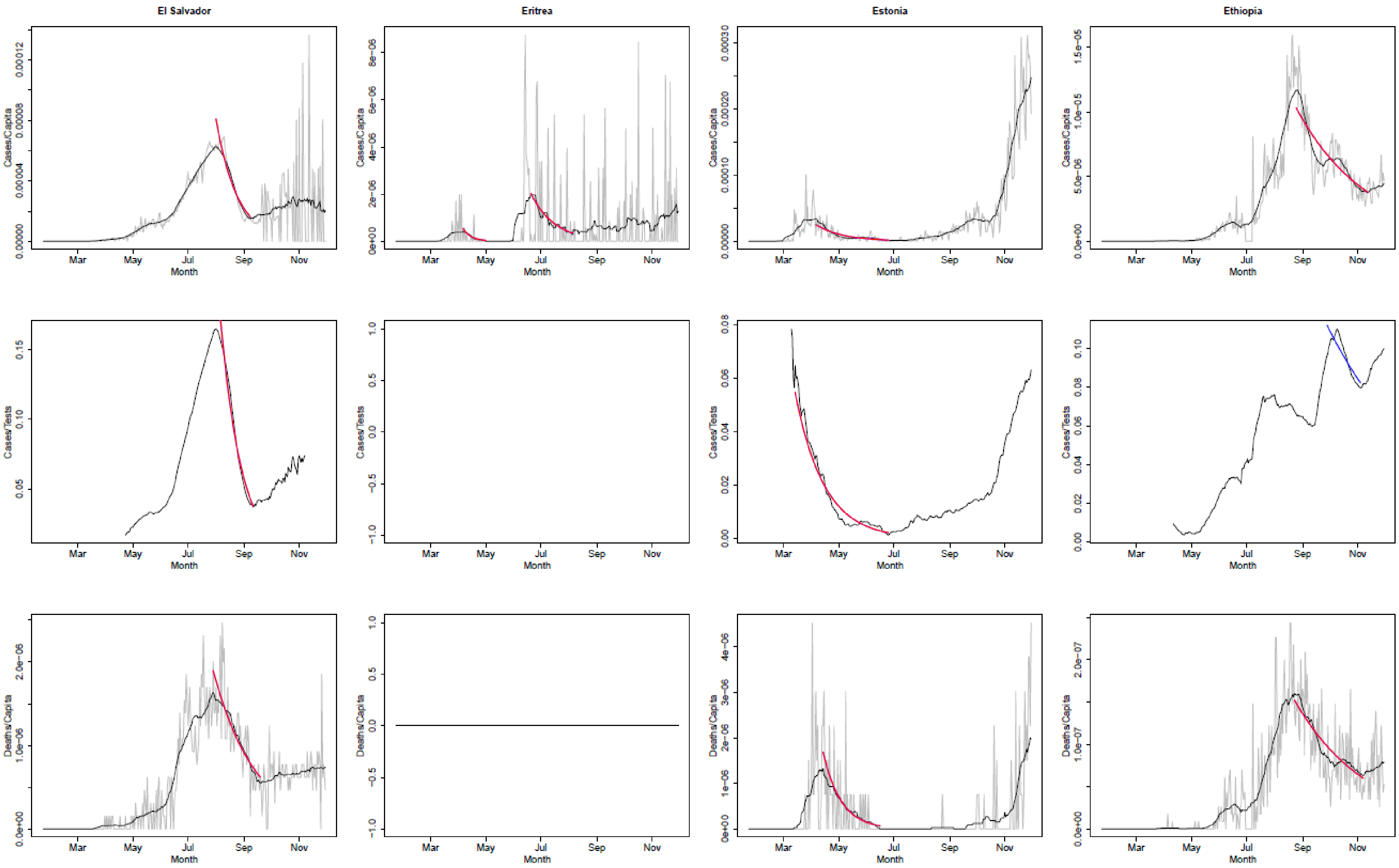
El Salvador
Eritrea
Estonia
Ethiopia
Cases/Capita
Cases/Tests
Deaths/Capita
Month
Mar
May
Jul
Sep
Nov

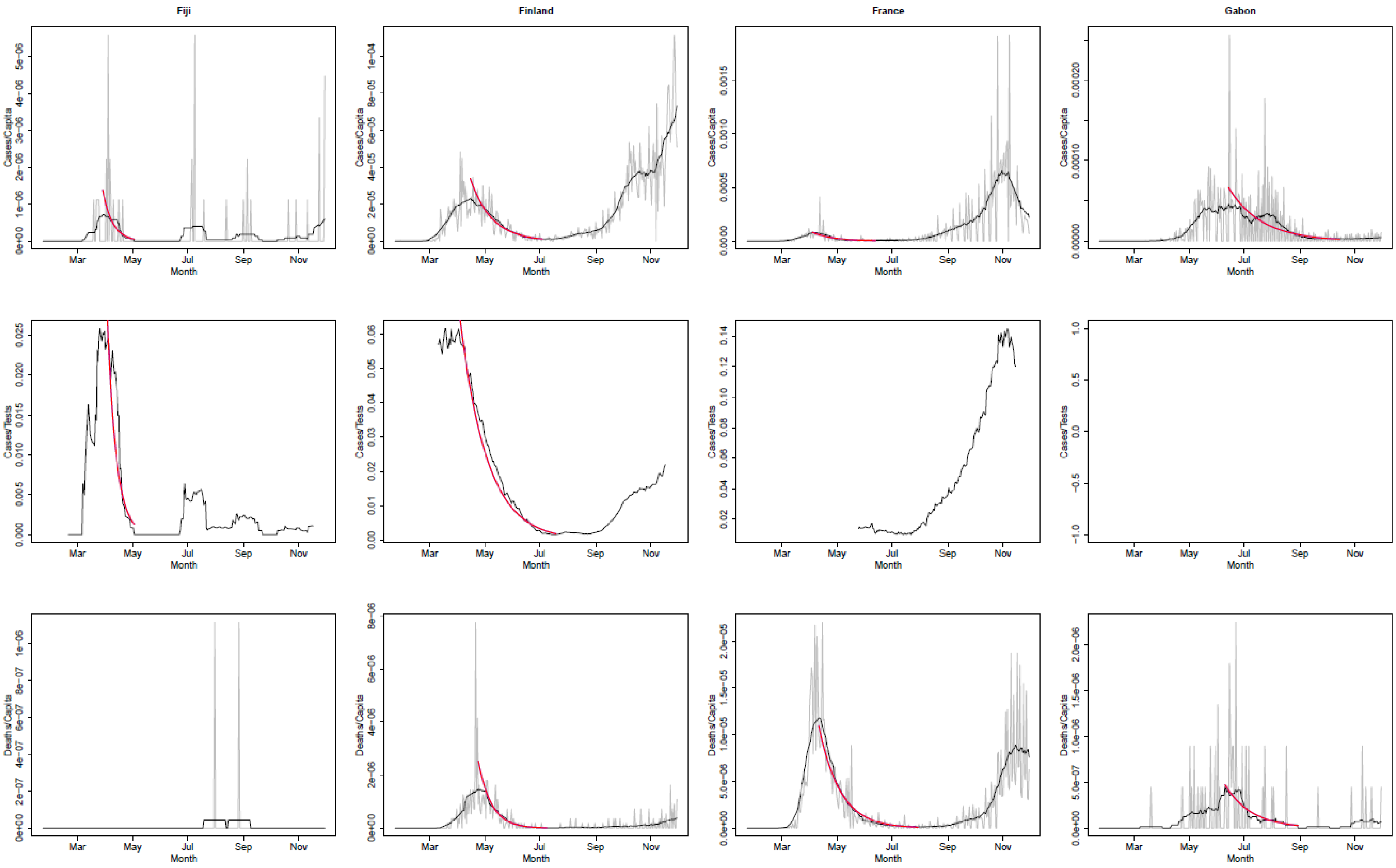
Fiji
Finland
France
Gabon
Cases/Capita
Cases/Tests
Deaths/Capita
Month
Mar
May
Jul
Sep
Nov

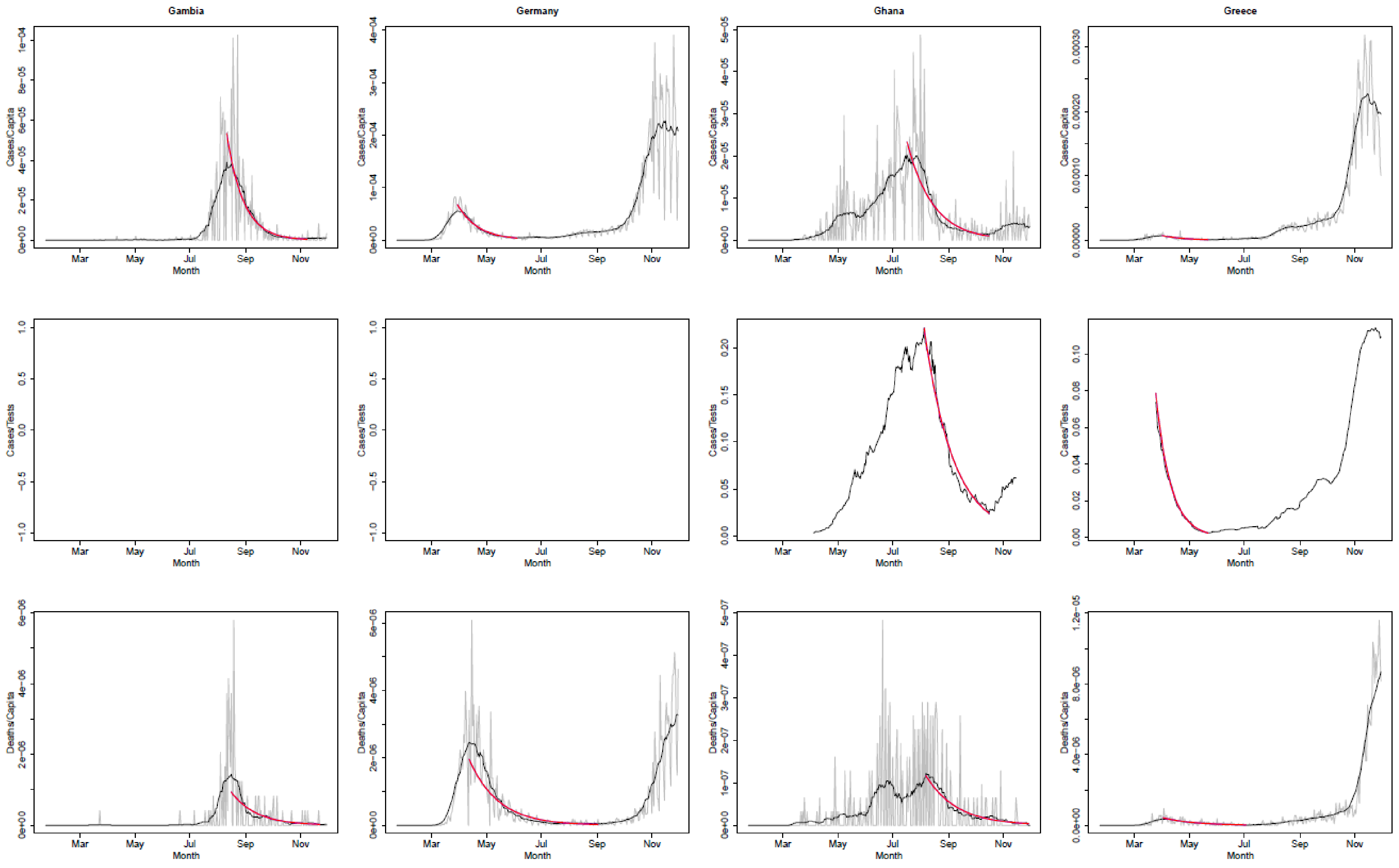
Gambia
Germany
Ghana
Greece
Cases/Capita
Cases/Tests
Deaths/Capita
Month
Mar
May
Jul
Sep
Nov

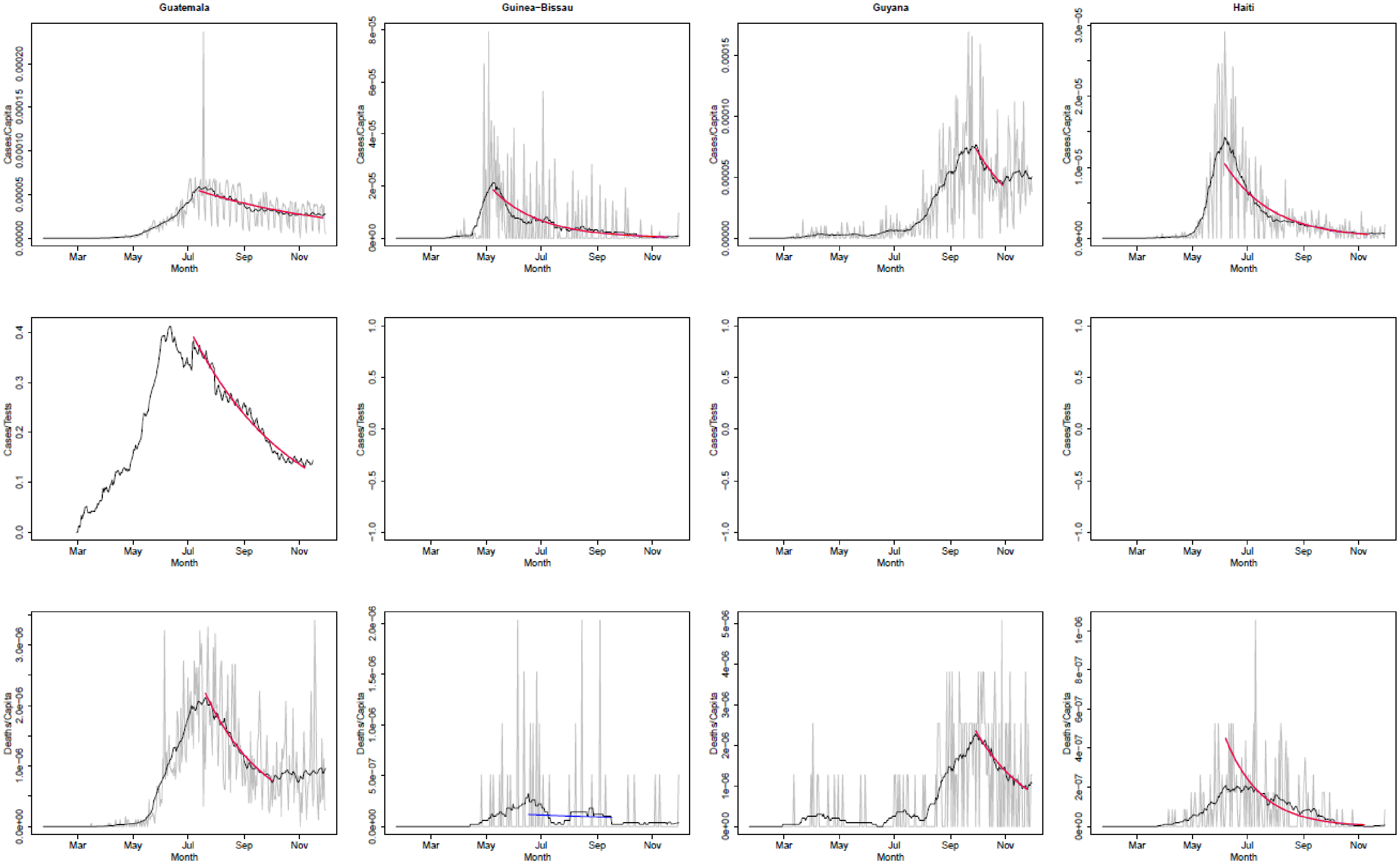
Guatemala
Guinea-Bissau
Guyana
Haiti
Cases/Capita
Cases/Tests
Deaths/Capita
Month
Mar
May
Jul
Sep
Nov

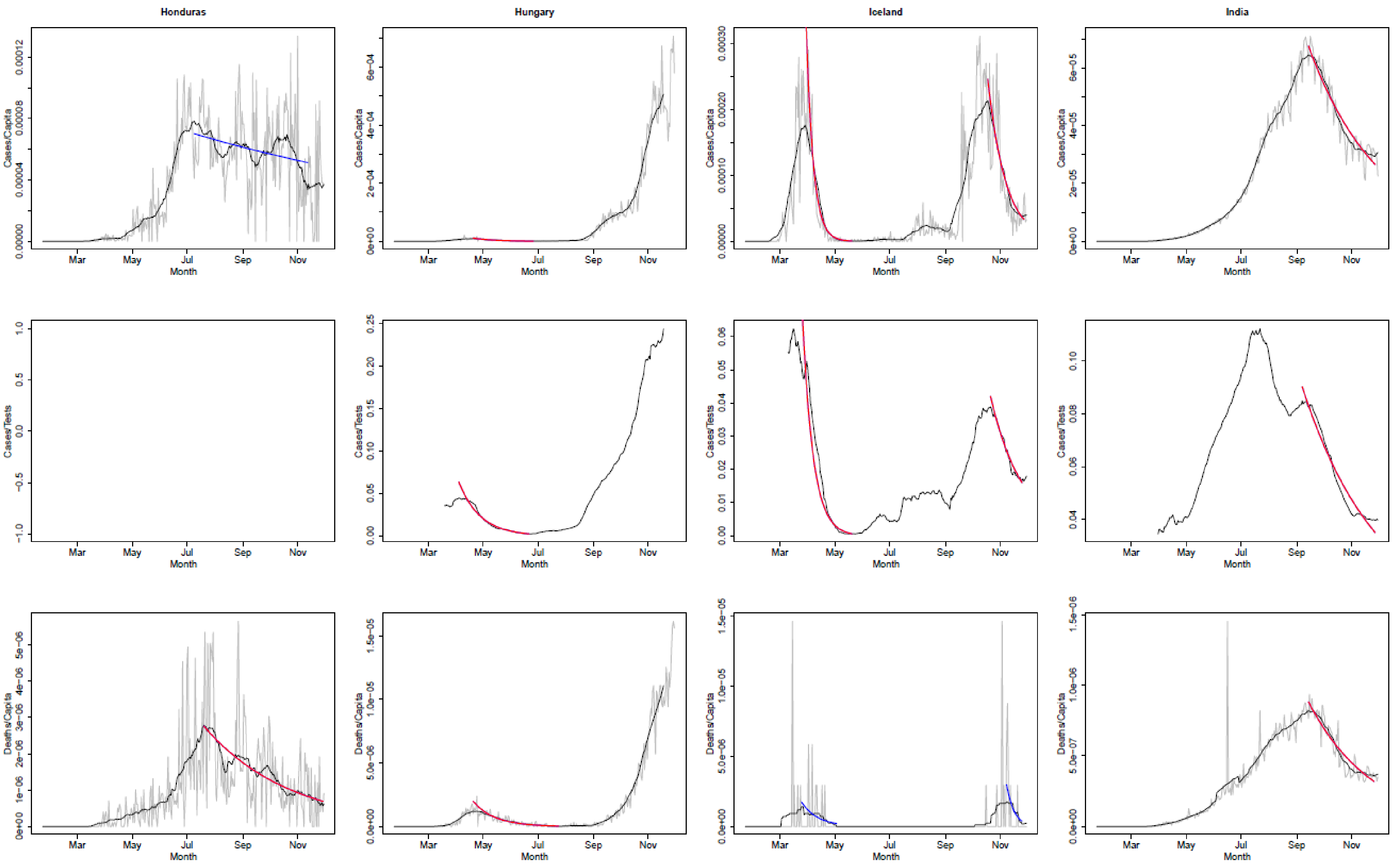
Honduras
Hungary
Iceland
India
Cases/Capita
Cases/Tests
Deaths/Capita
Month
Mar
May
Jul
Sep
Nov
0.00000
0.00004
0.00008
0.00012
0e+00
2e-04
4e-04
6e-04
0.00000
0.00010
0.00020
0.00030
0e+00
2e-05
4e-05
6e-05
-1.0
-0.5
0.0
0.5
1.0
0.00
0.05
0.10
0.15
0.20
0.25
0.00
0.01
0.02
0.03
0.04
0.05
0.06
0.04
0.06
0.08
0.10
0e+00
1e-06
2e-06
3e-06
4e-06
5e-06
0.0e+00
5.0e-06
1.0e-05
1.5e-05
0.0e+00
5.0e-06
1.0e-05
1.5e-05
0.0e+00
5.0e-07
1.0e-06
1.5e-06

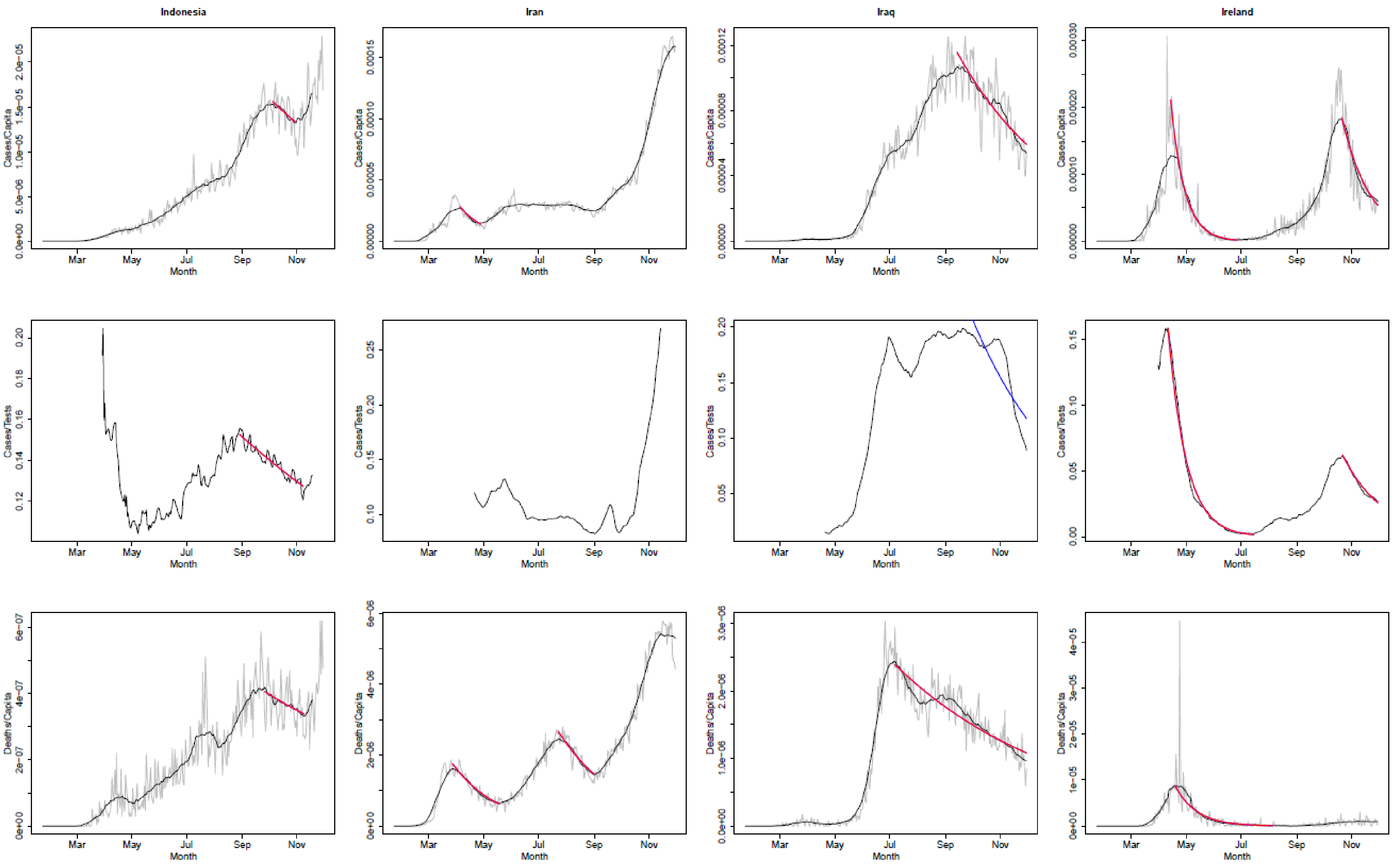
Indonesia
Iran
Iraq
Ireland
Cases/Capita
Cases/Tests
Deaths/Capita
Month
Mar
May
Jul
Sep
Nov

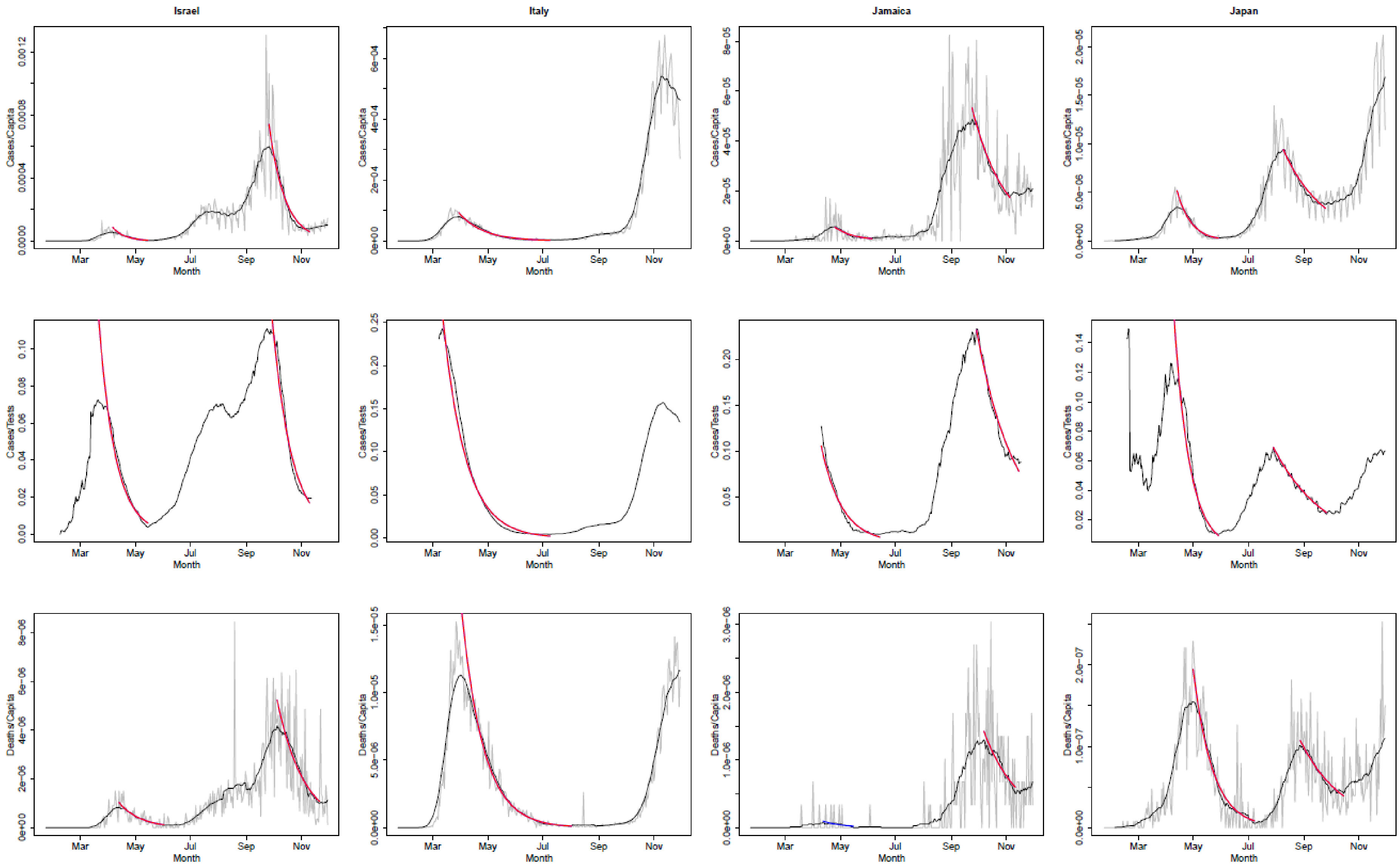
Israel
Italy
Jamaica
Japan
Cases/Capita
Cases/Tests
Deaths/Capita
Mar
May
Jul
Sep
Nov
Month

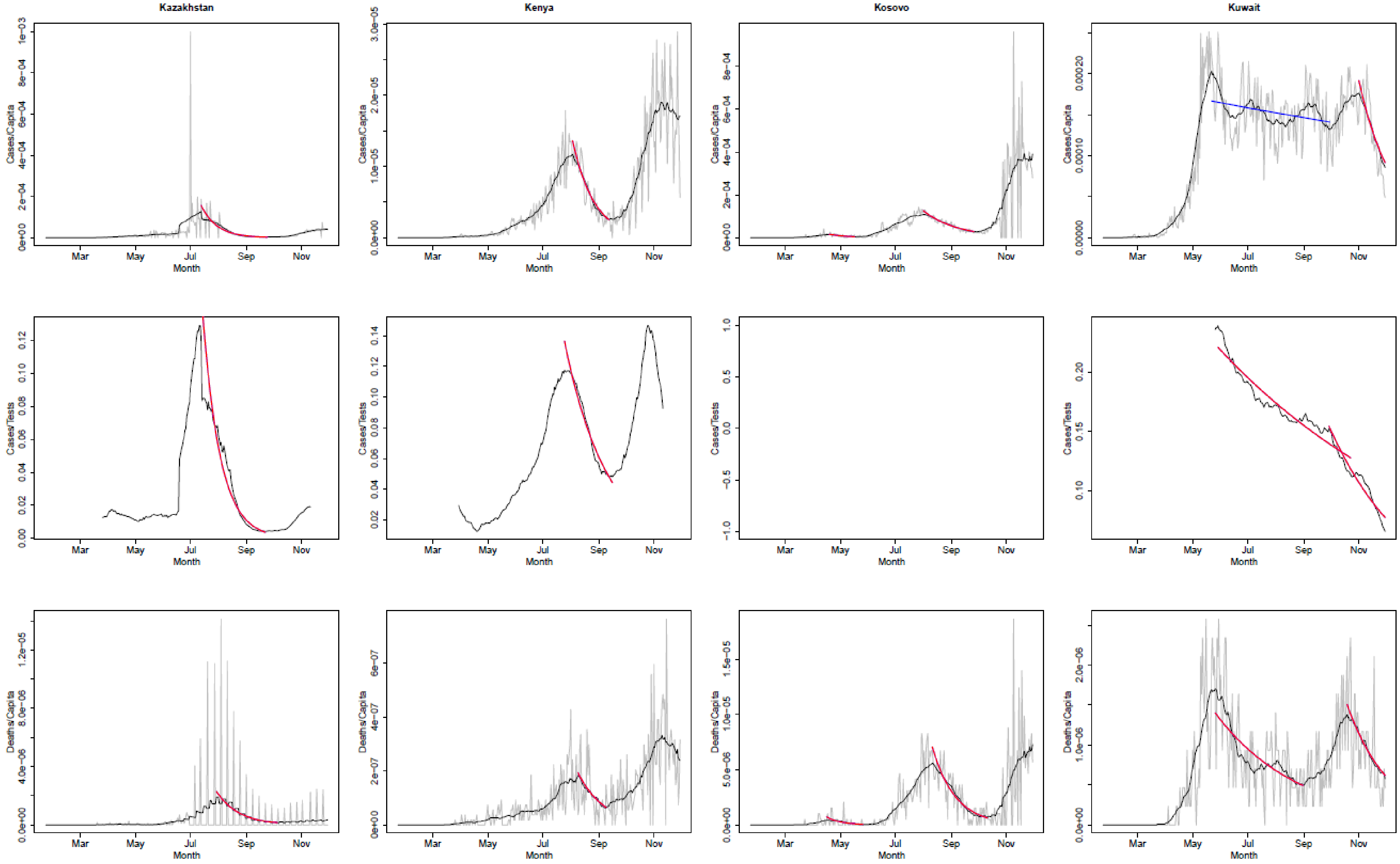

Kazakhstan
Kenya
Kosovo
Kuwait
Cases/Capita
Cases/Tests
Deaths/Capita
Mar
May
Jul
Sep
Nov
Month

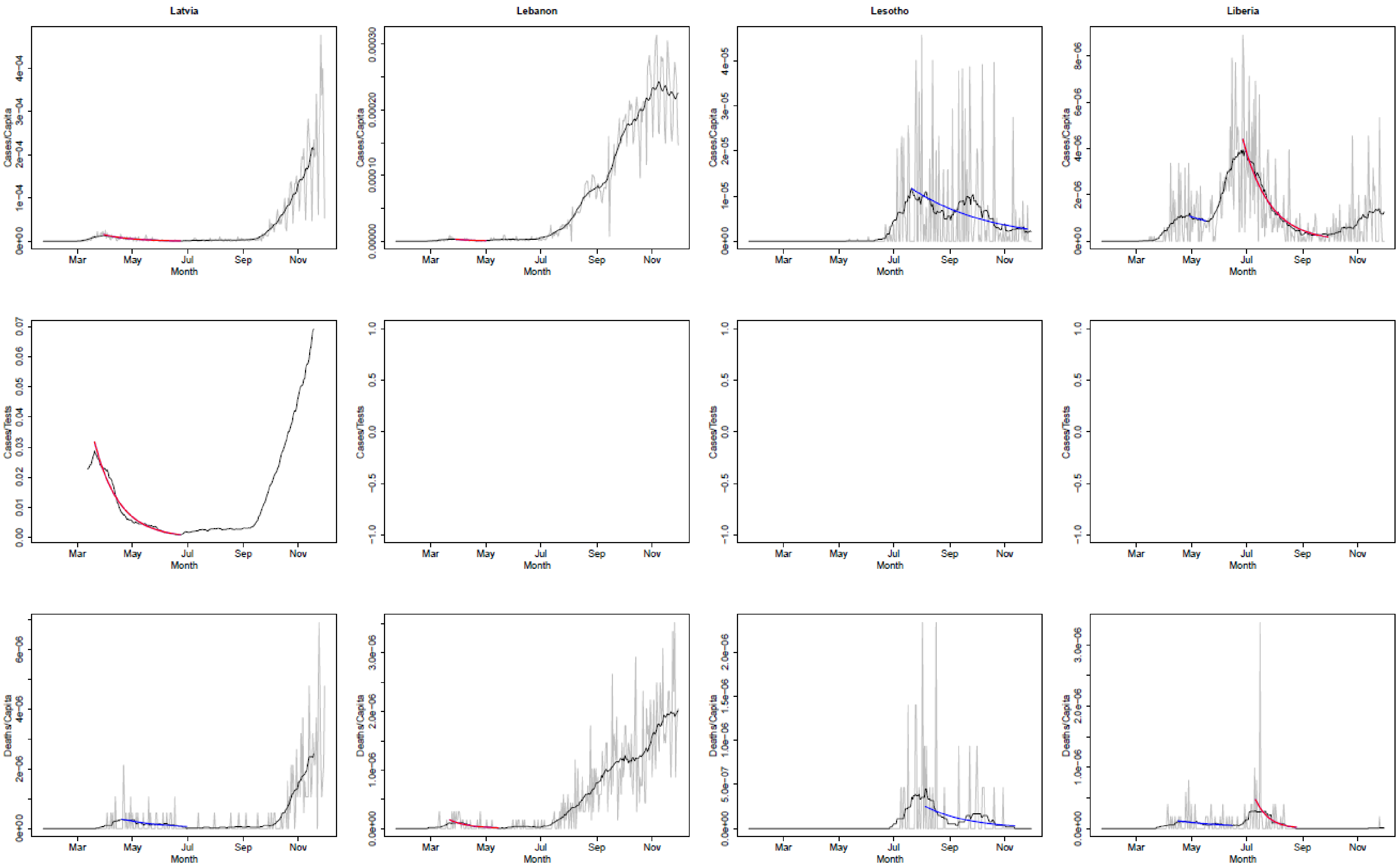

Latvia
Lebanon
Lesotho
Liberia
Cases/Capita
Cases/Tests
Deaths/Capita
Month
Mar
May
Jul
Sep
Nov

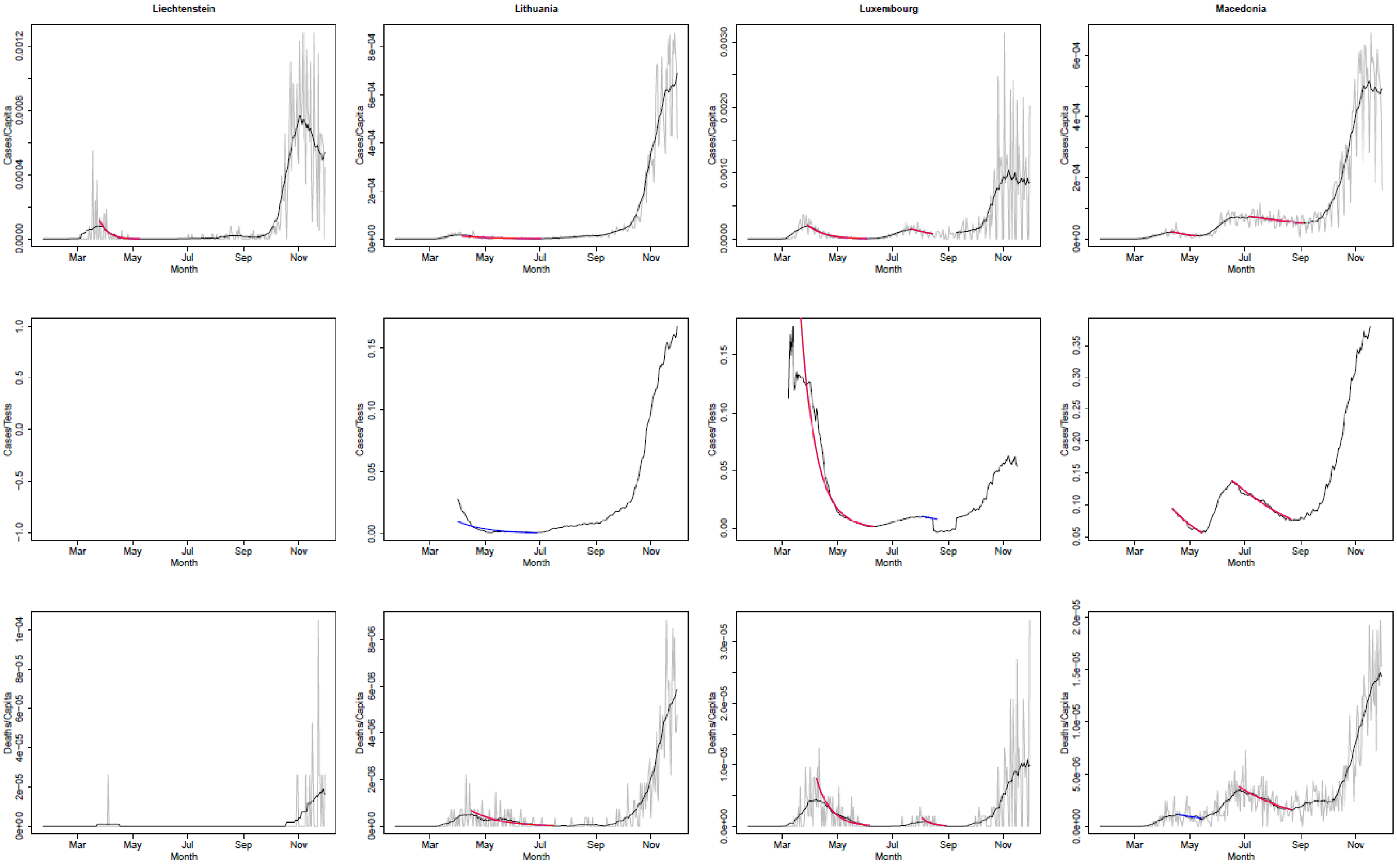
Liechtenstein
Lithuania
Luxembourg
Macedonia
Cases/Capita
Cases/Tests
Deaths/Capita
Mar
May
Jul
Sep
Nov
Month

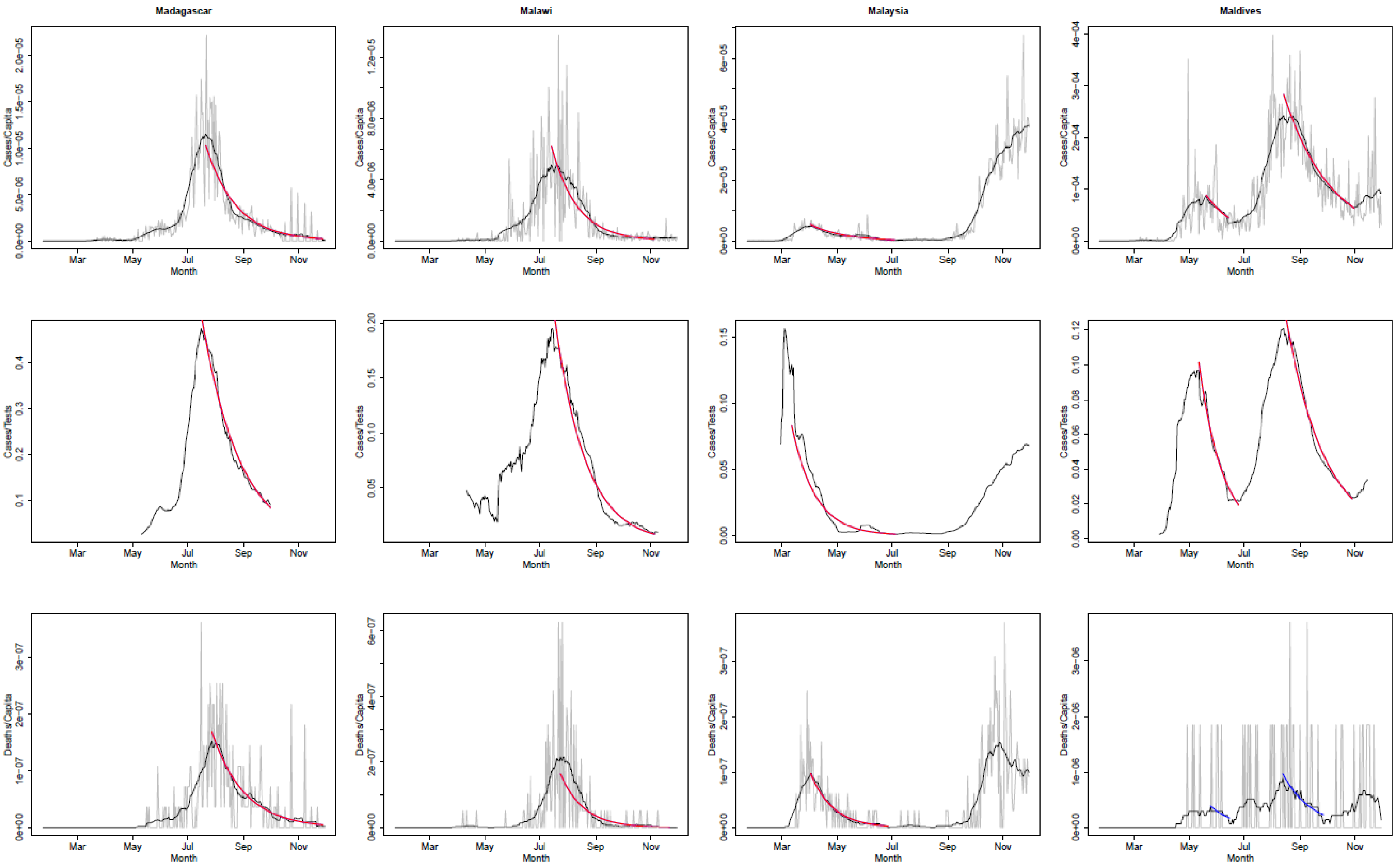
Madagascar
Malawi
Malaysia
Maldives
Cases/Capita
Cases/Tests
Deaths/Capita
Month
Mar
May
Jul
Sep
Nov

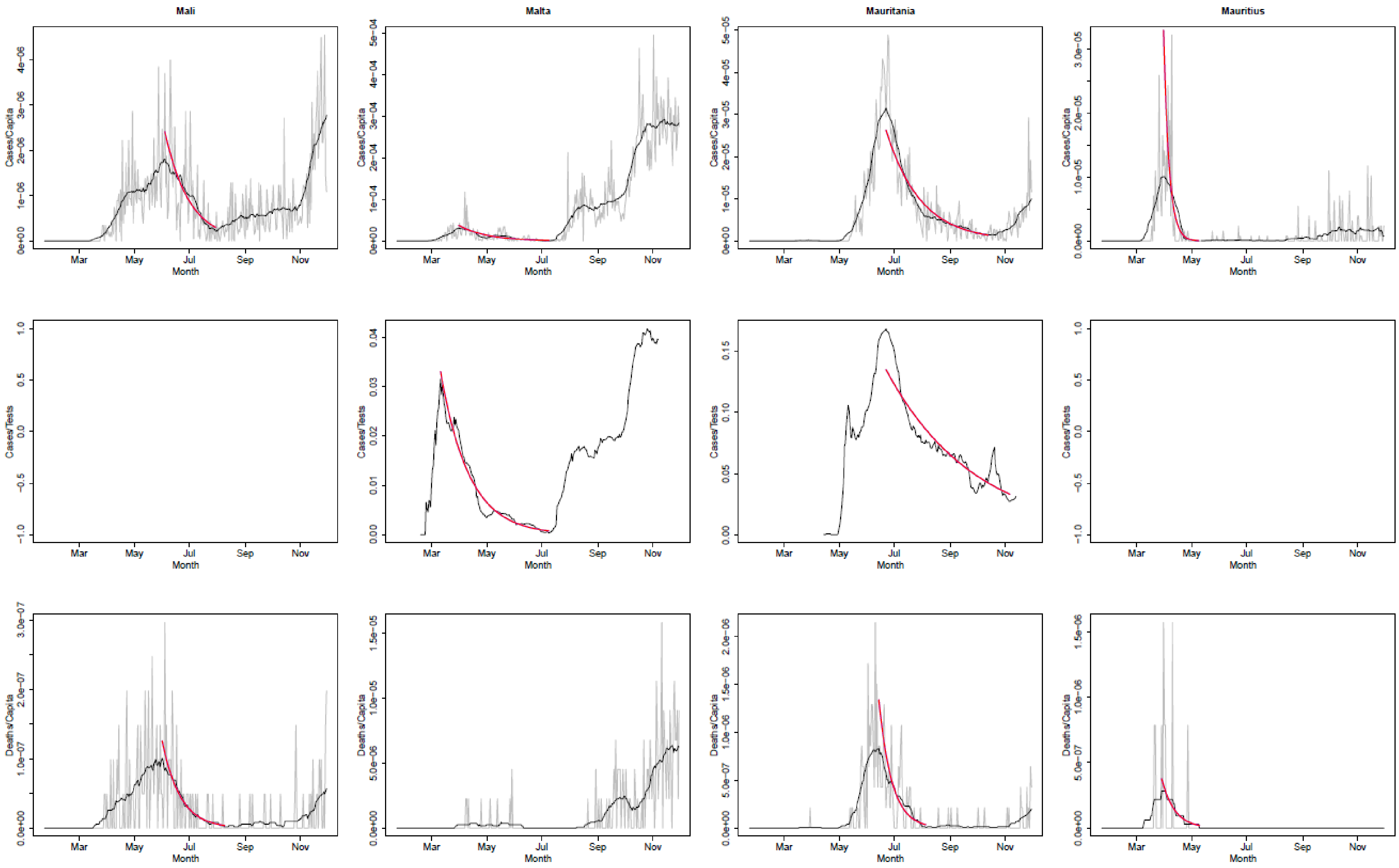

Mali
Malta
Mauritania
Mauritius
Cases/Capita
Cases/Tests
Deaths/Capita
Mar
May
Jul
Sep
Nov
Month

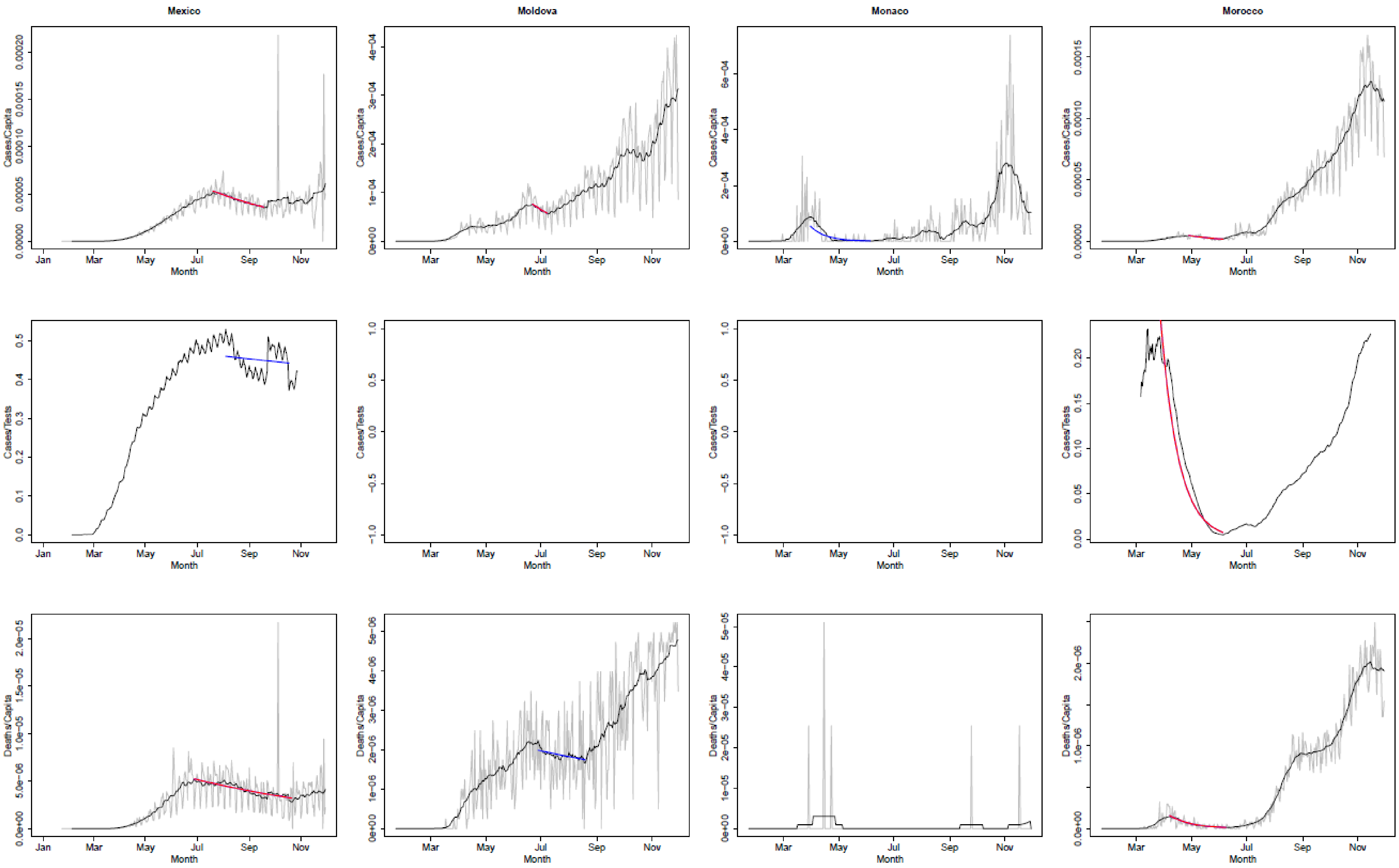
Mexico
Moldova
Monaco
Morocco
Cases/Capita
Cases/Tests
Deaths/Capita
Month
Jan
Mar
May
Jul
Sep
Nov

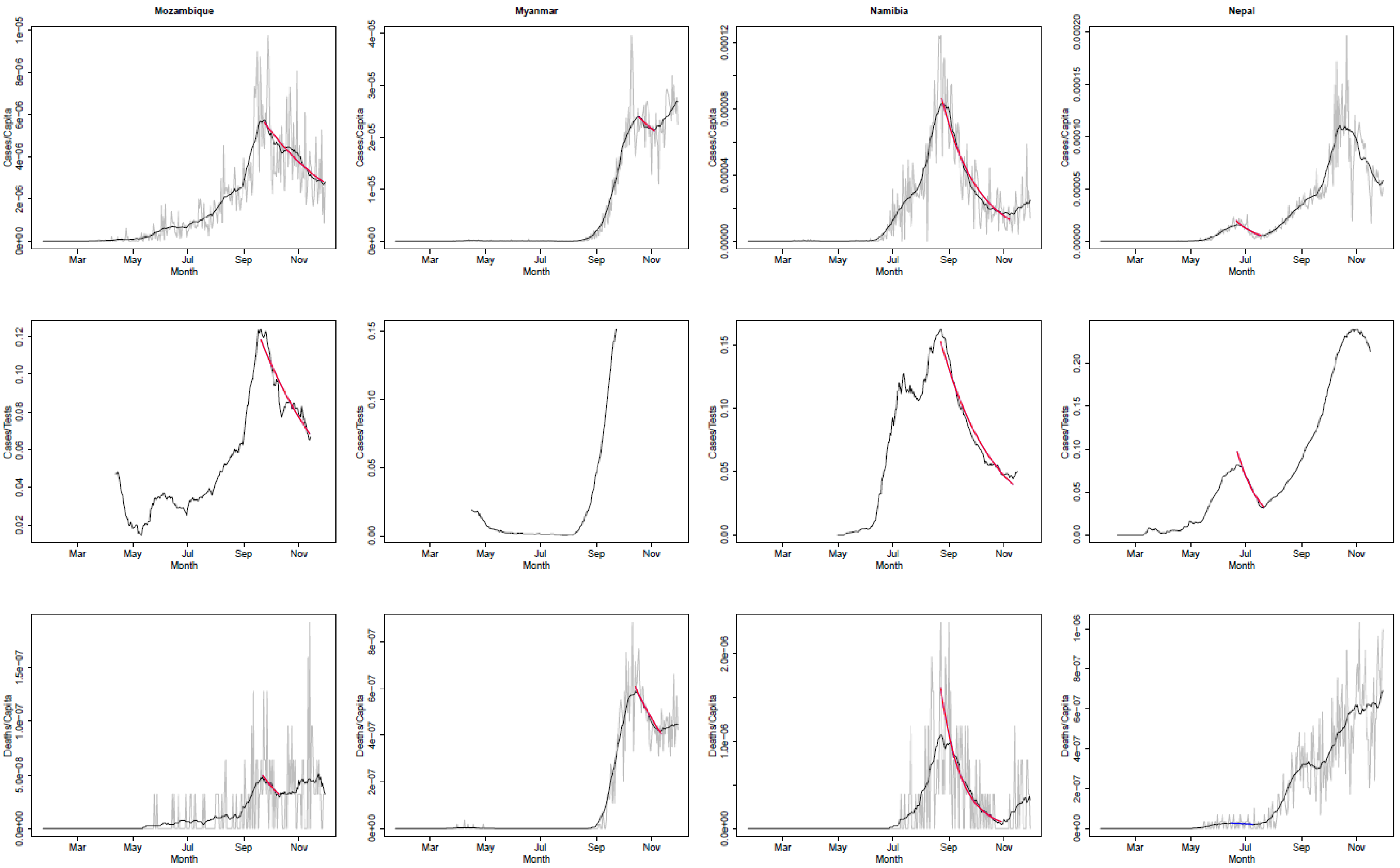

Mozambique
Myanmar
Namibia
Nepal
Cases/Capita
Cases/Tests
Deaths/Capita
Mar
May
Jul
Sep
Nov
Month

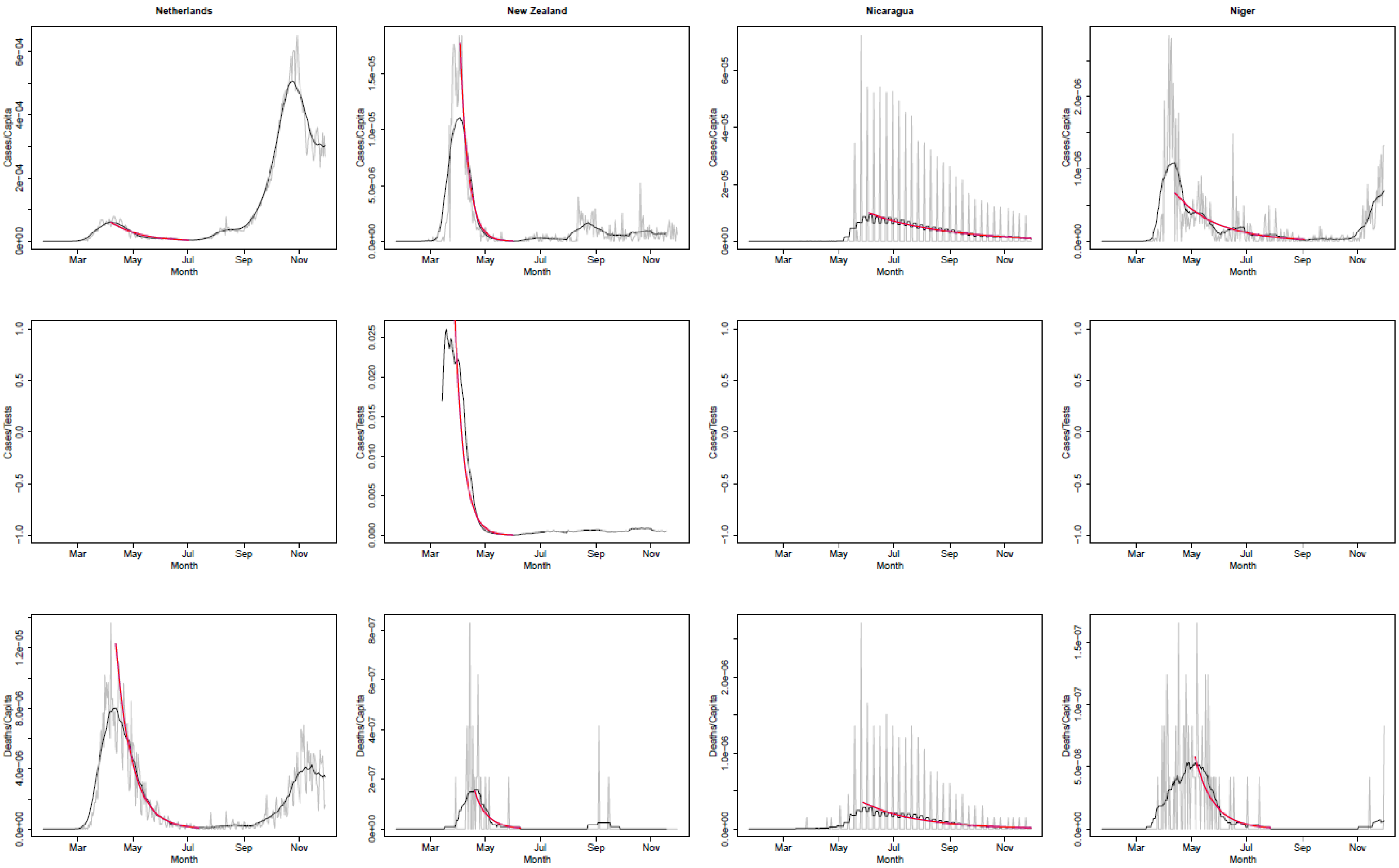
Netherlands
New Zealand
Nicaragua
Niger
Cases/Capita
Cases/Tests
Deaths/Capita
Mar
May
Jul
Sep
Nov
Month

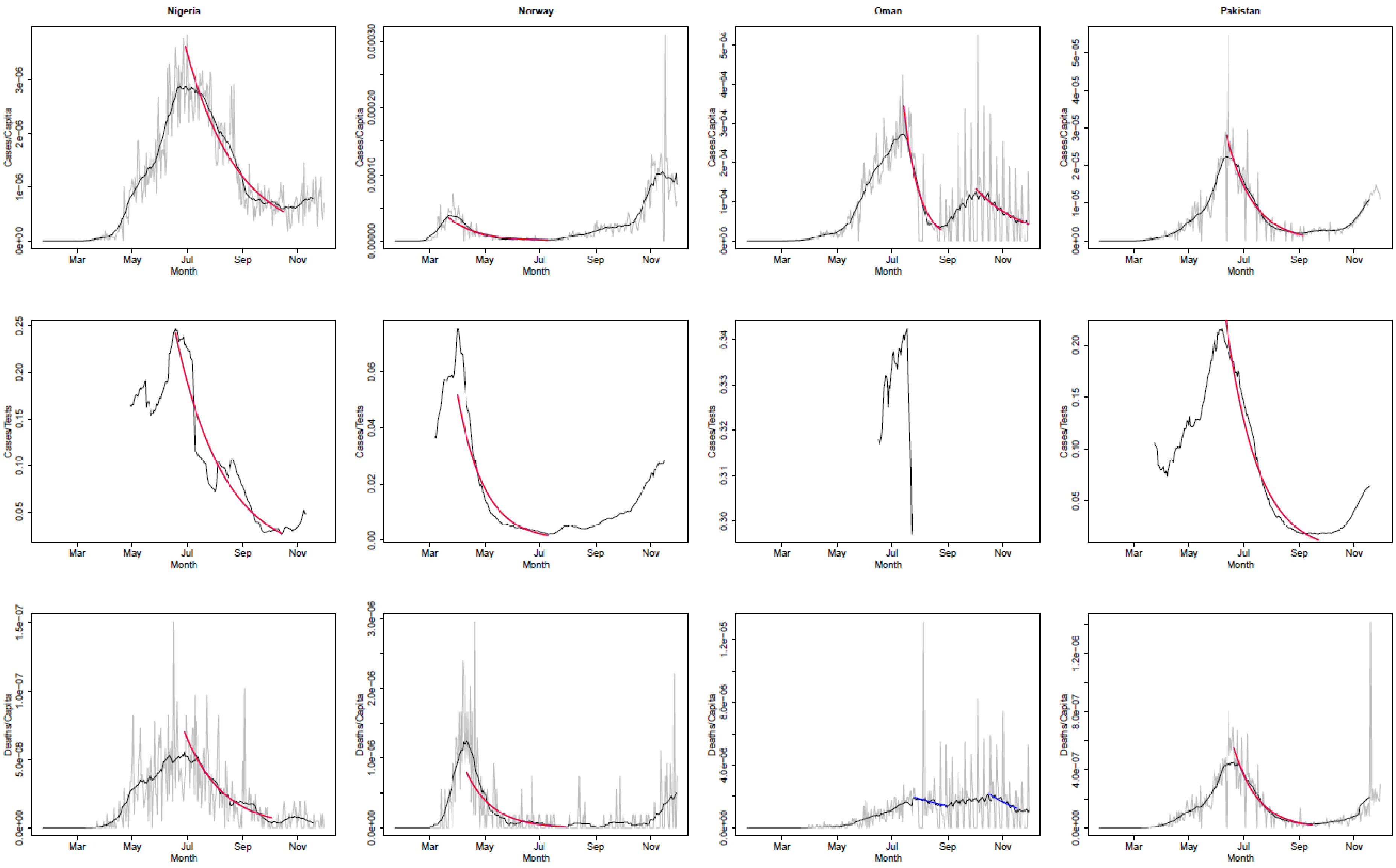

Nigeria
Norway
Oman
Pakistan
Cases/Capita
Cases/Tests
Deaths/Capita
Mar
May
Jul
Sep
Nov
Month

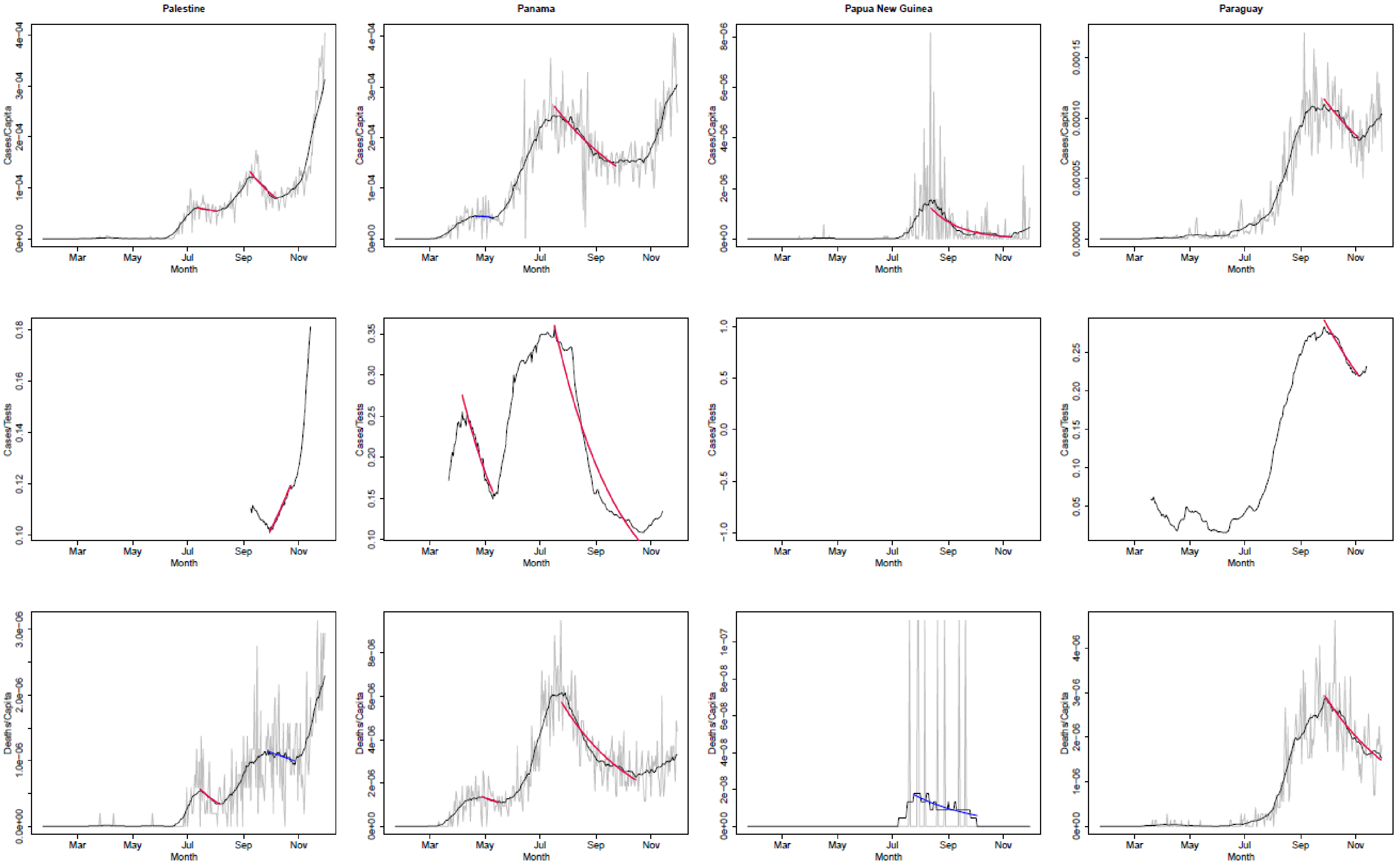
Palestine
Panama
Papua New Guinea
Paraguay
Cases/Capita
Cases/Tests
Deaths/Capita
Mar
May
Jul
Sep
Nov
Month

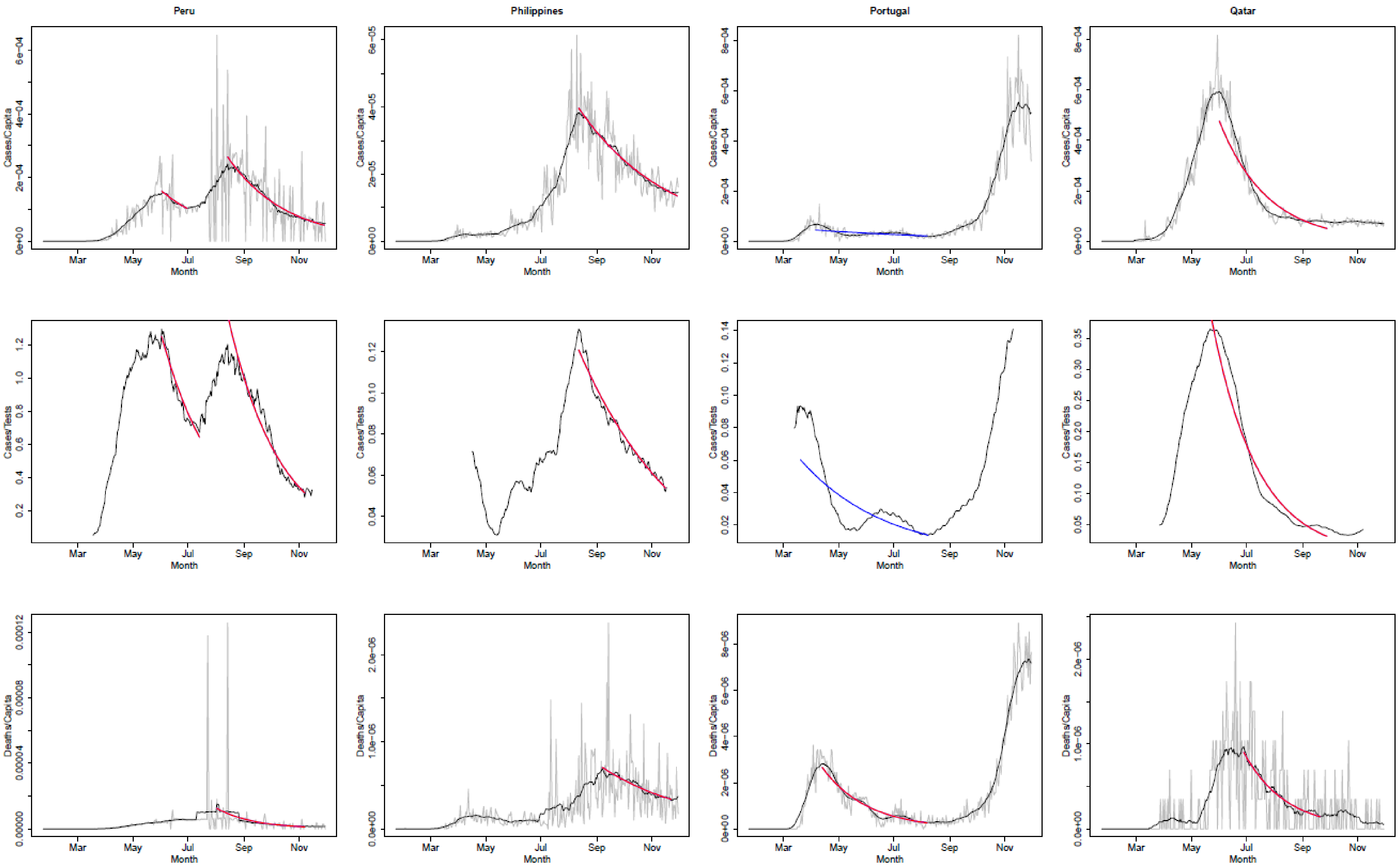

Peru
Philippines
Portugal
Qatar
Cases/Capita
Cases/Tests
Deaths/Capita
Month
Mar
May
Jul
Sep
Nov

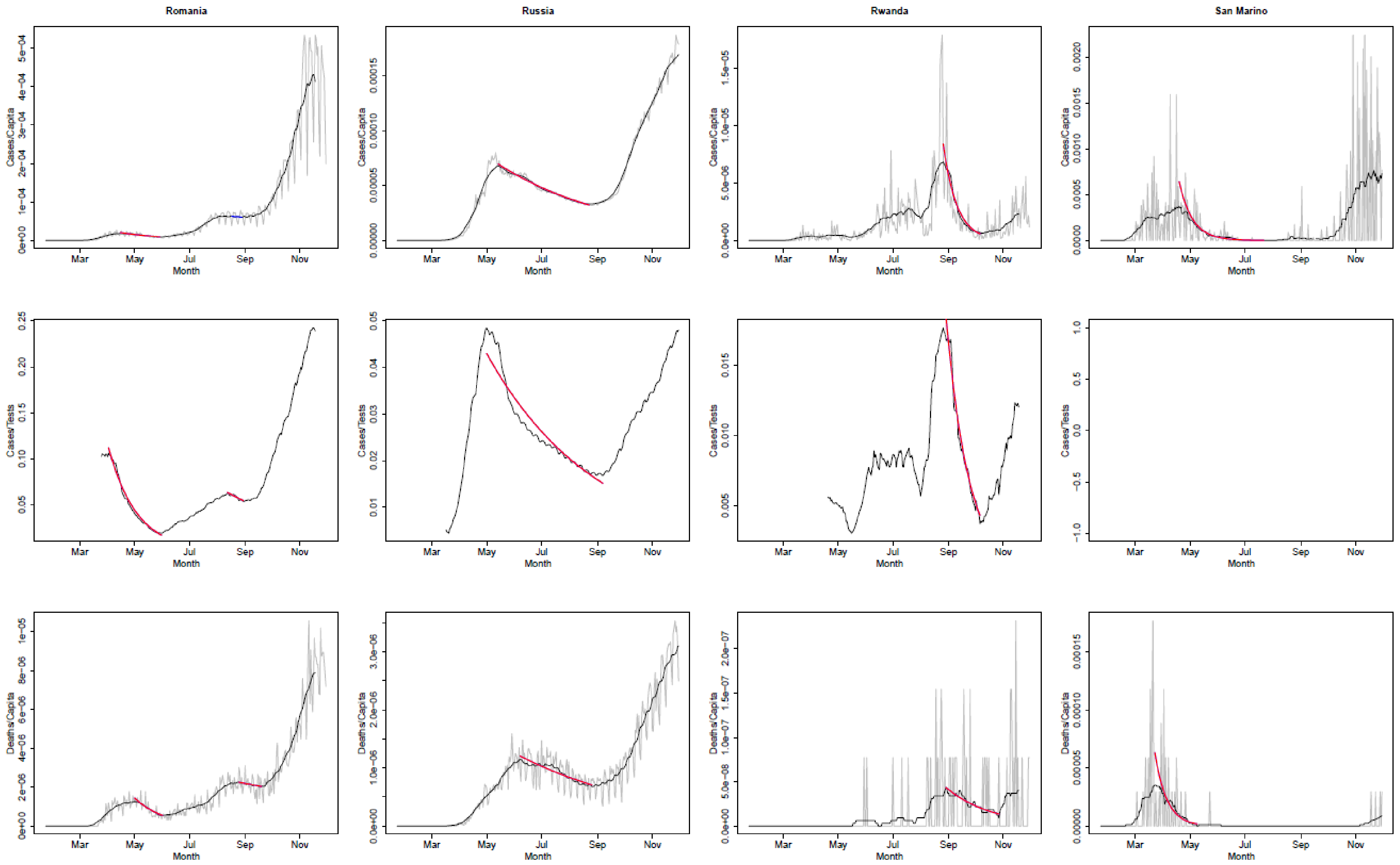

Romania
Russia
Rwanda
San Marino
Cases/Capita
Cases/Tests
Deaths/Capita
Month
Mar
May
Jul
Sep
Nov

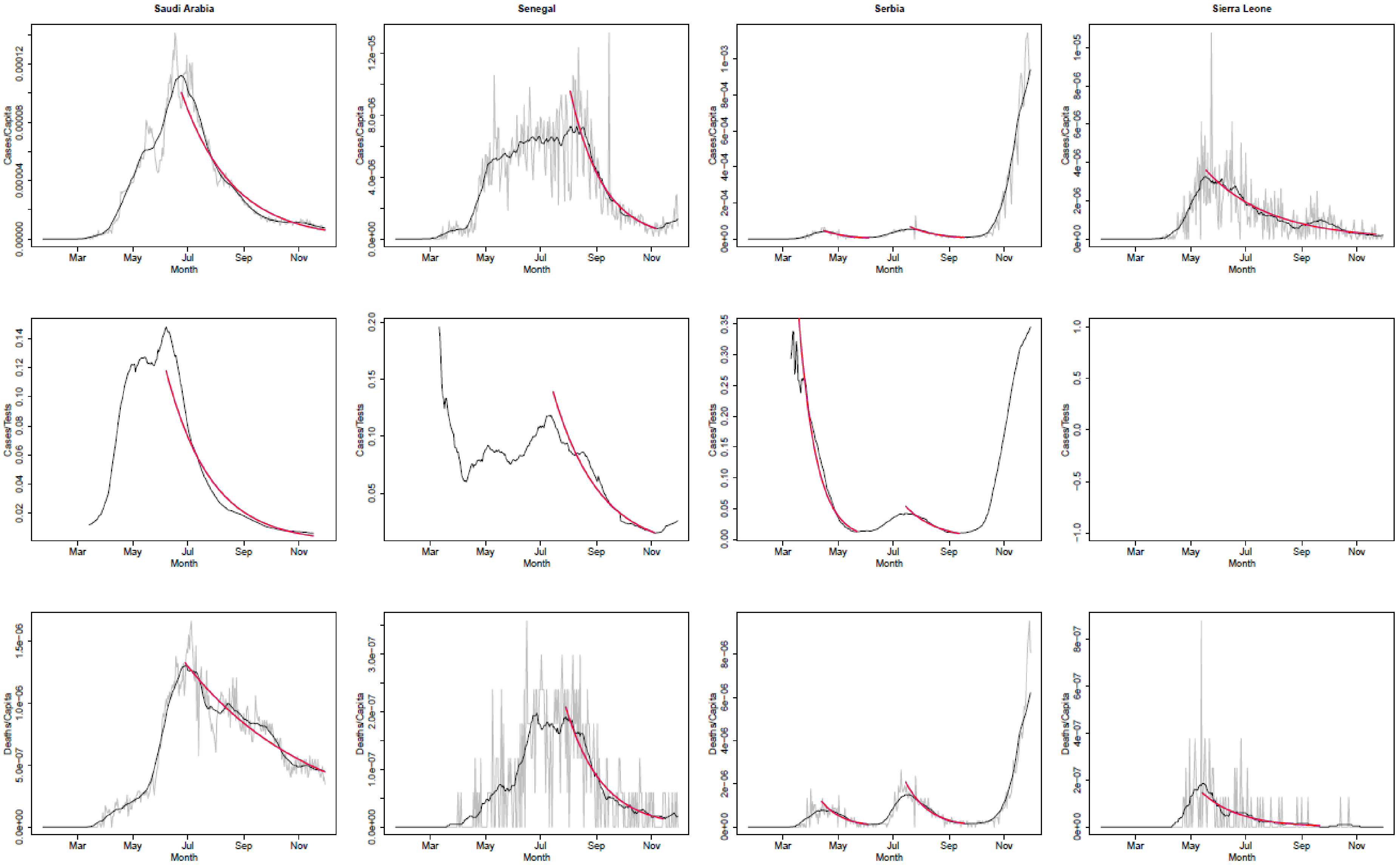

Saudi Arabia
Senegal
Serbia
Sierra Leone
Cases/Capita
Cases/Tests
Deaths/Capita
Mar
May
Jul
Sep
Nov
Month

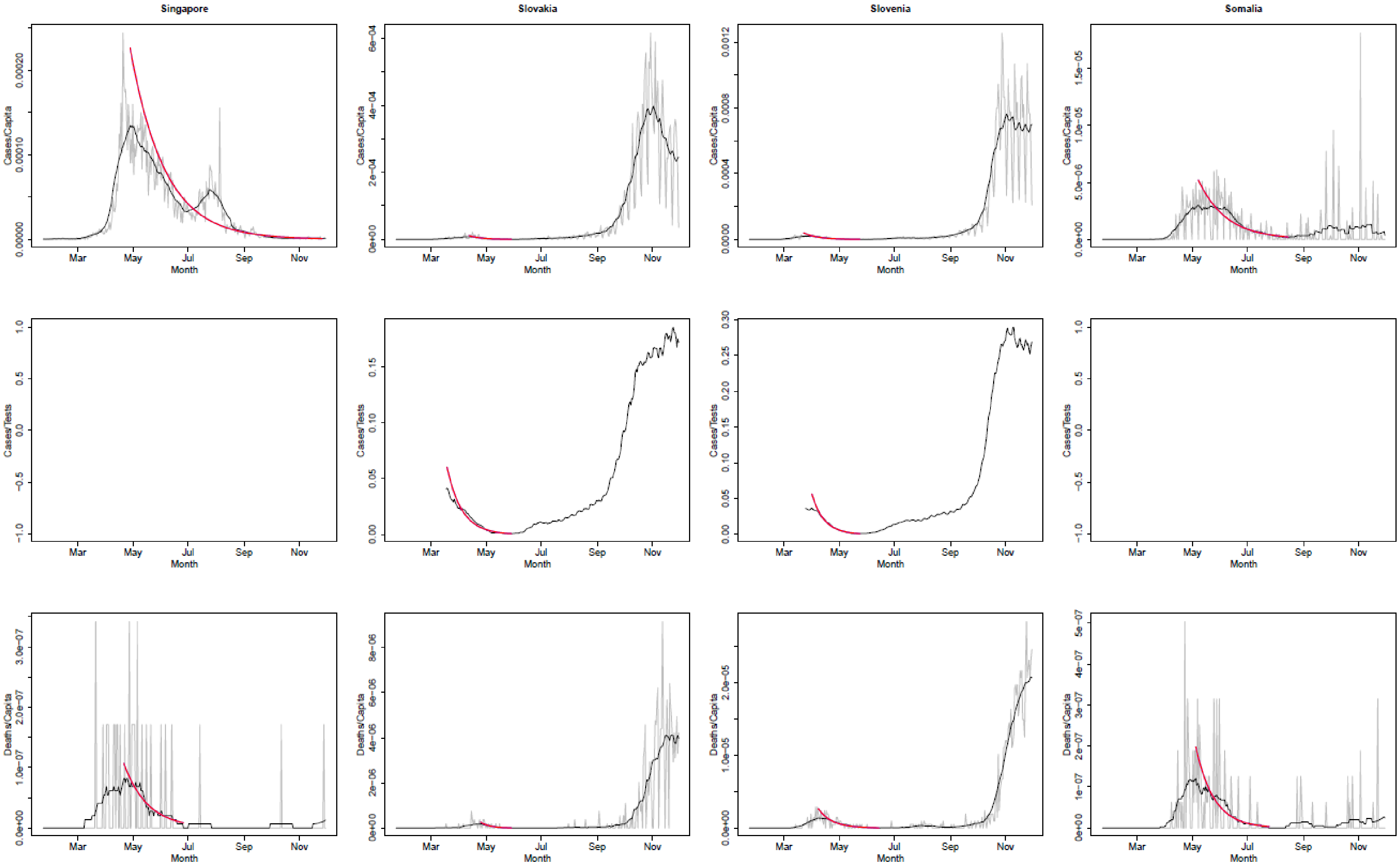
Singapore
Slovakia
Slovenia
Somalia
Cases/Capita
Cases/Tests
Deaths/Capita
Month
Mar
May
Jul
Sep
Nov

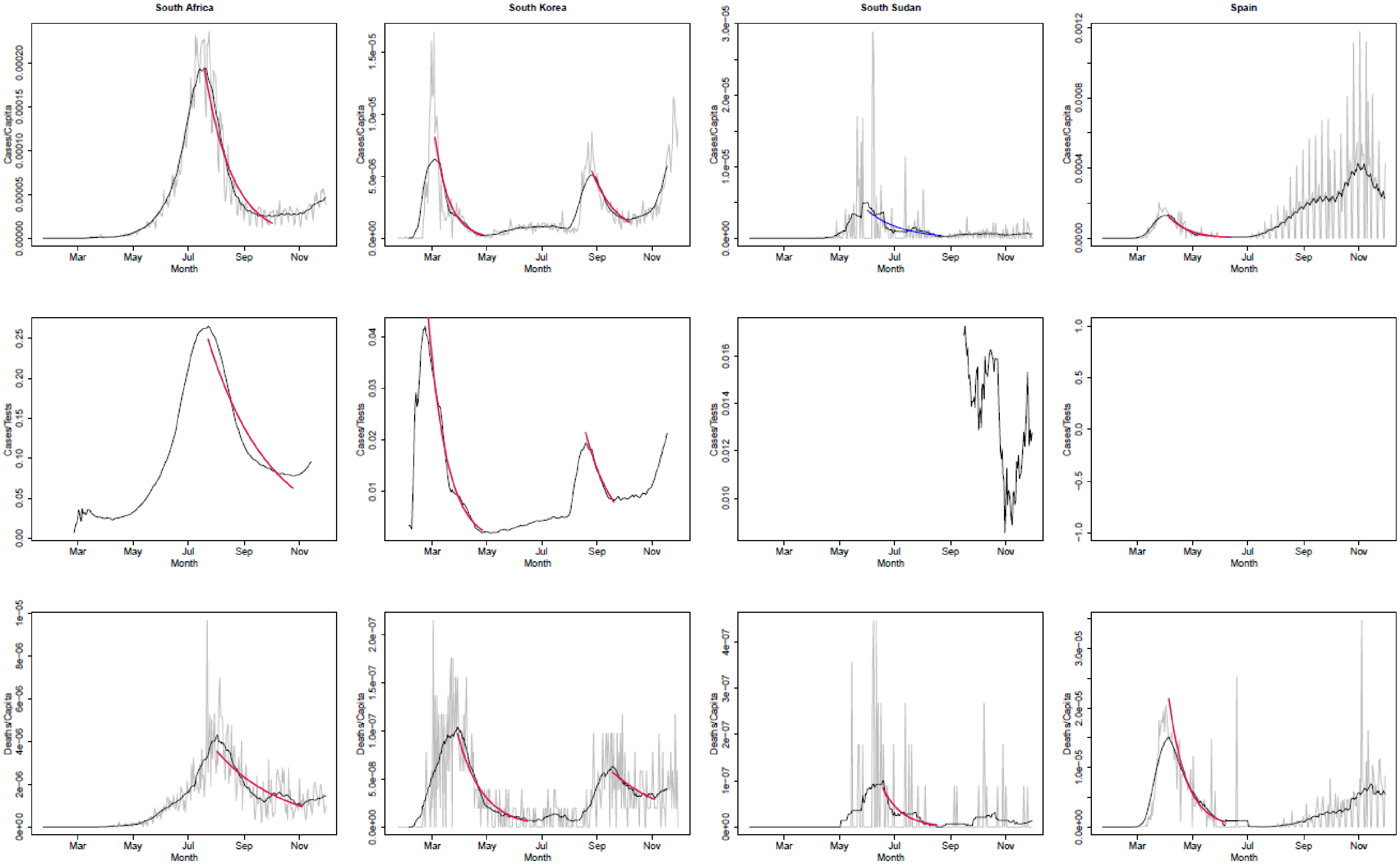

South Africa
South Korea
South Sudan
Spain
Cases/Capita
Cases/Tests
Deaths/Capita
Month
Mar
May
Jul
Sep
Nov

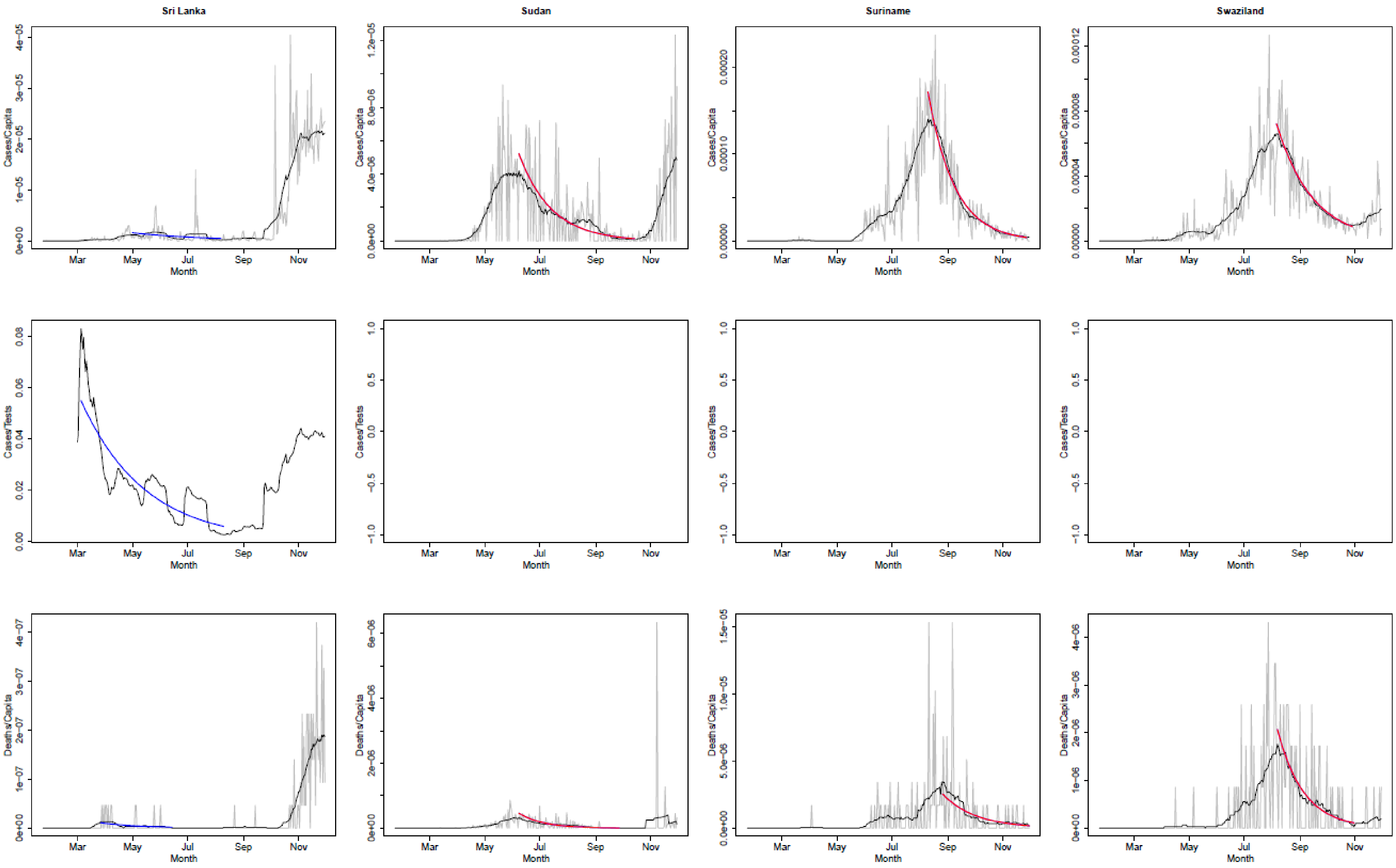

Sri Lanka
Sudan
Suriname
Swaziland
Cases/Capita
Cases/Tests
Deaths/Capita
Month
Mar
May
Jul
Sep
Nov

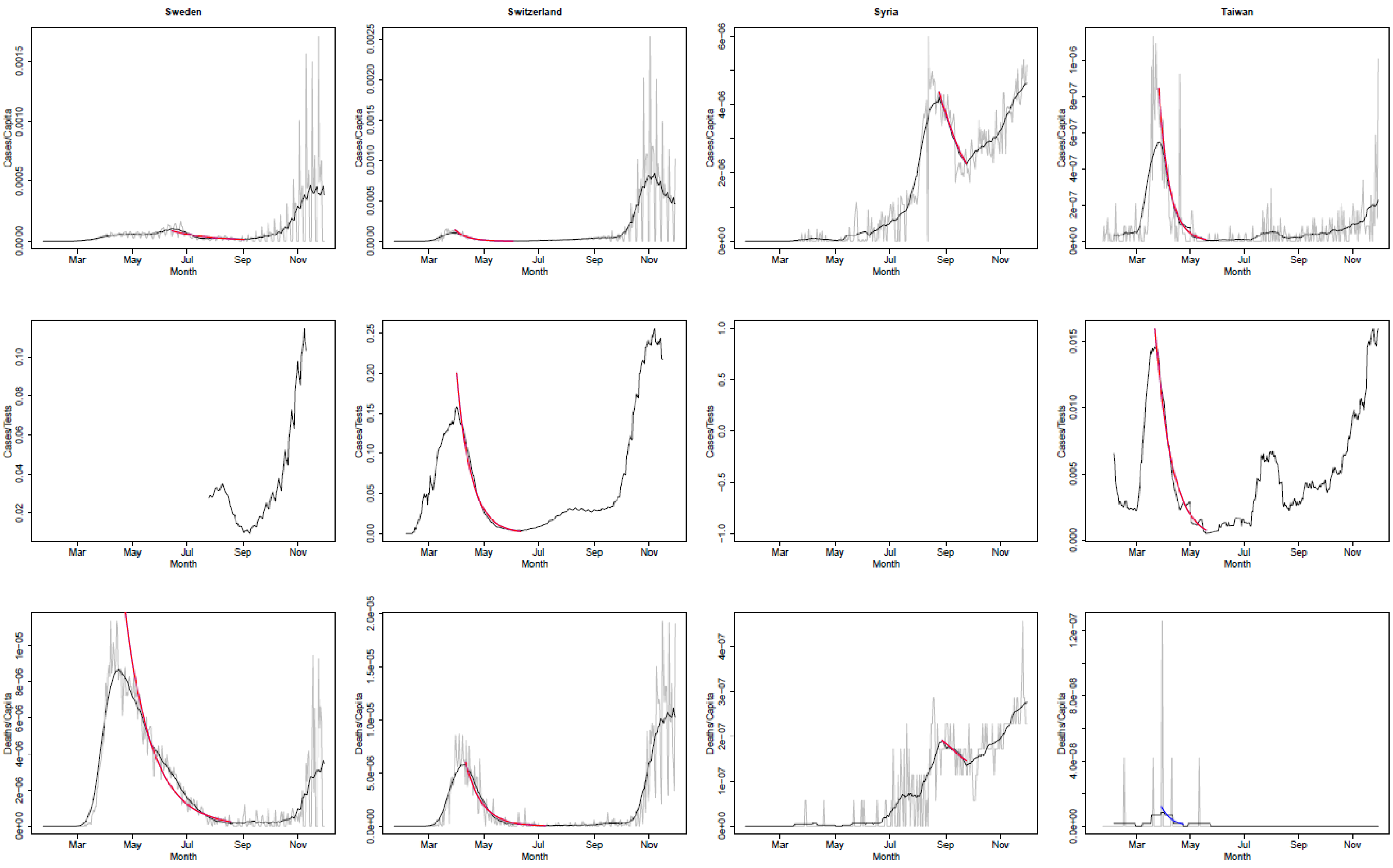
Sweden
Switzerland
Syria
Taiwan
Cases/Capita
Cases/Tests
Deaths/Capita
Mar
May
Jul
Sep
Nov
Month

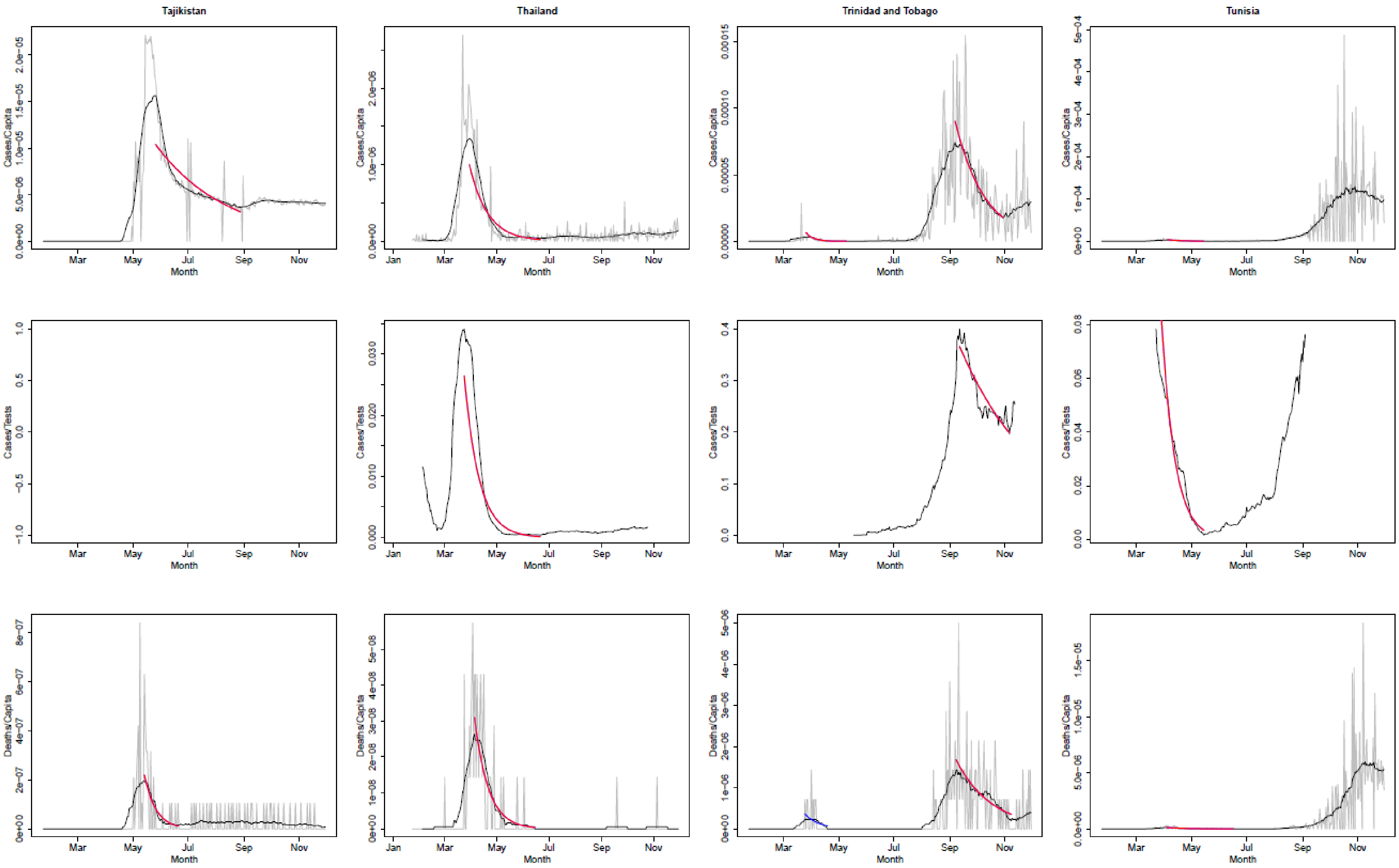
Tajikistan
Thailand
Trinidad and Tobago
Tunisia
Cases/Capita
Cases/Tests
Deaths/Capita
Month
Jan
Mar
May
Jul
Sep
Nov

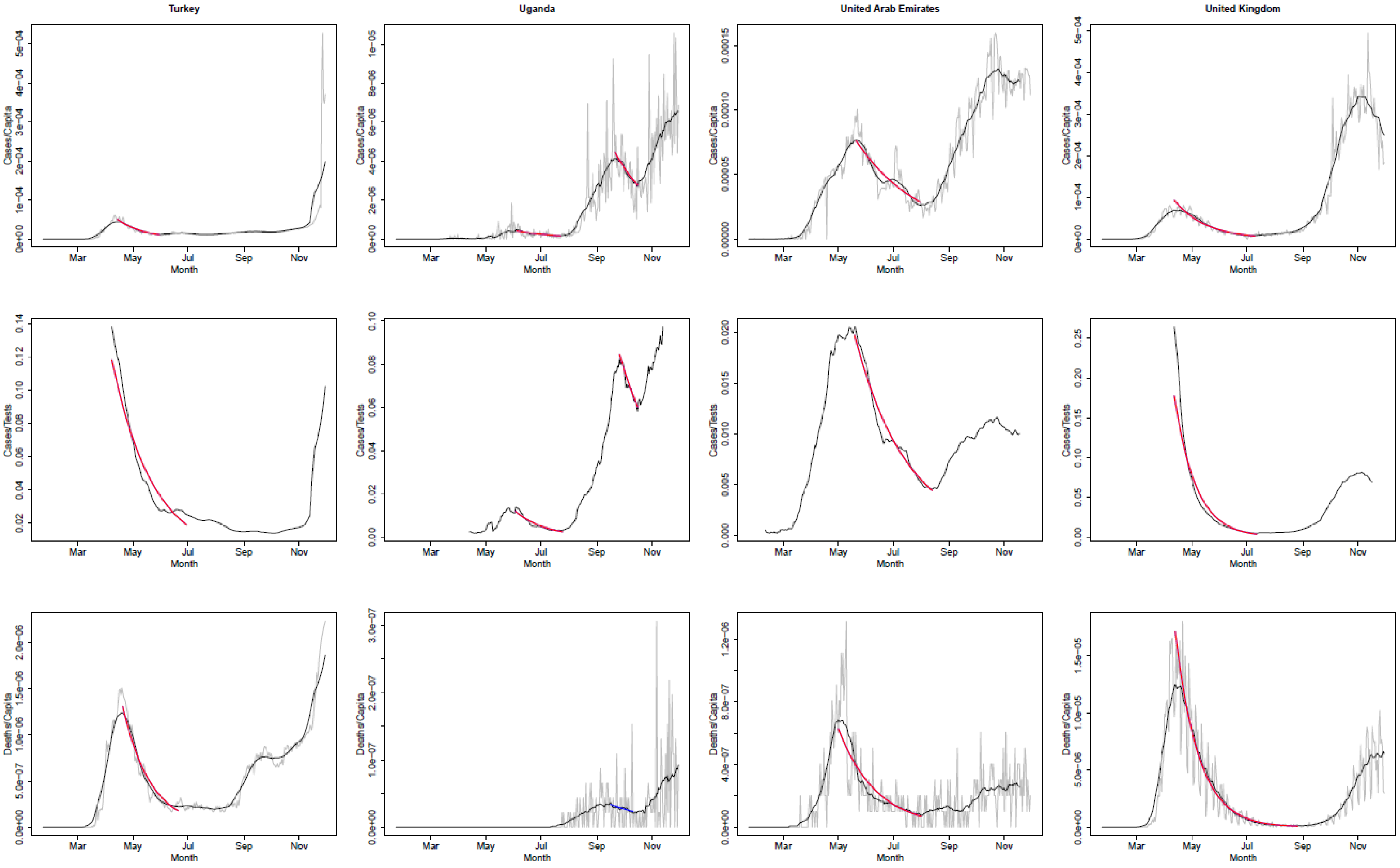
Turkey
Uganda
United Arab Emirates
United Kingdom
Cases/Capita
Cases/Tests
Deaths/Capita
Mar
May
Jul
Sep
Nov
Month

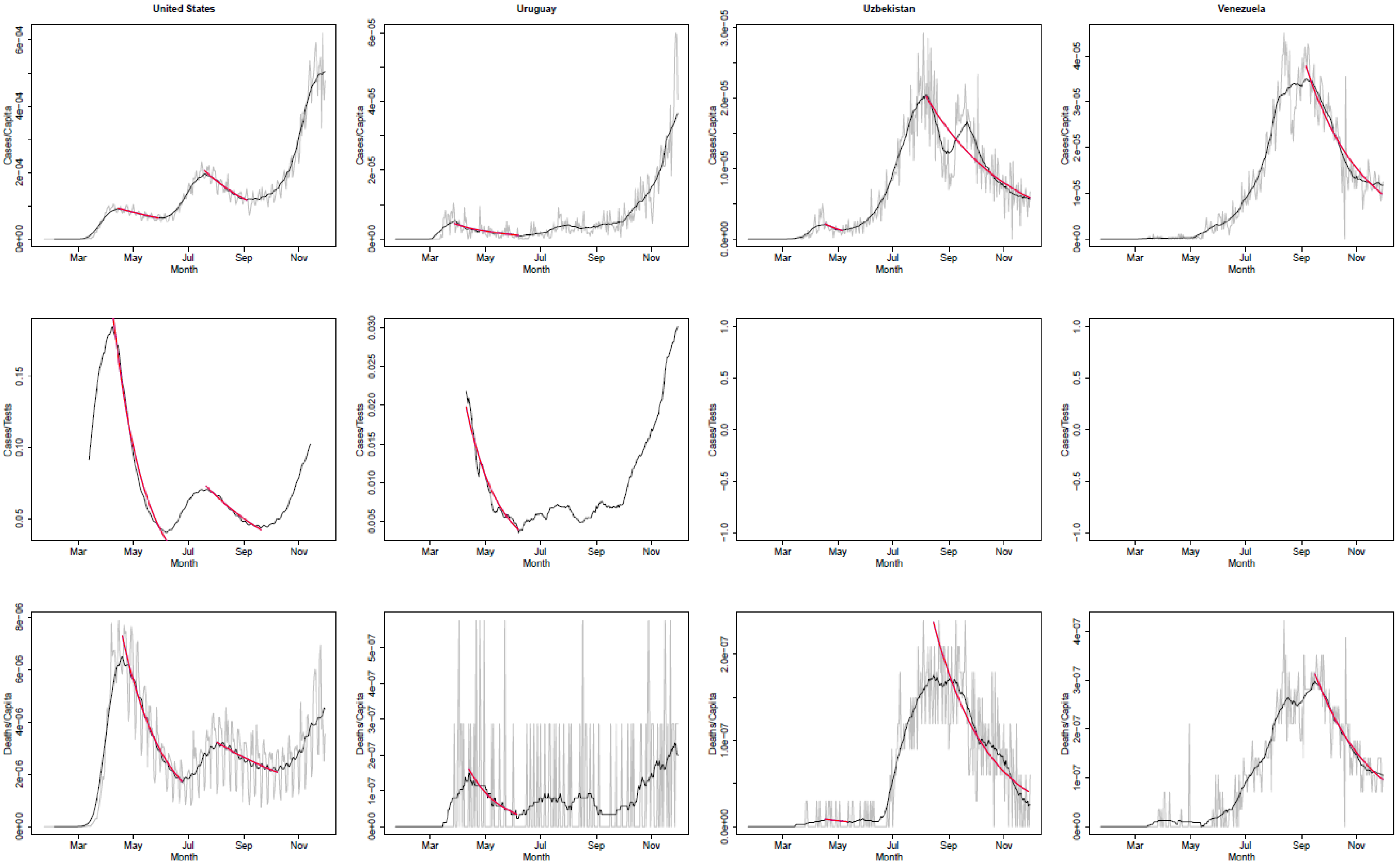
United States
Uruguay
Uzbekistan
Venezuela
Cases/Capita
Cases/Tests
Deaths/Capita
Mar
May
Jul
Sep
Nov
Month

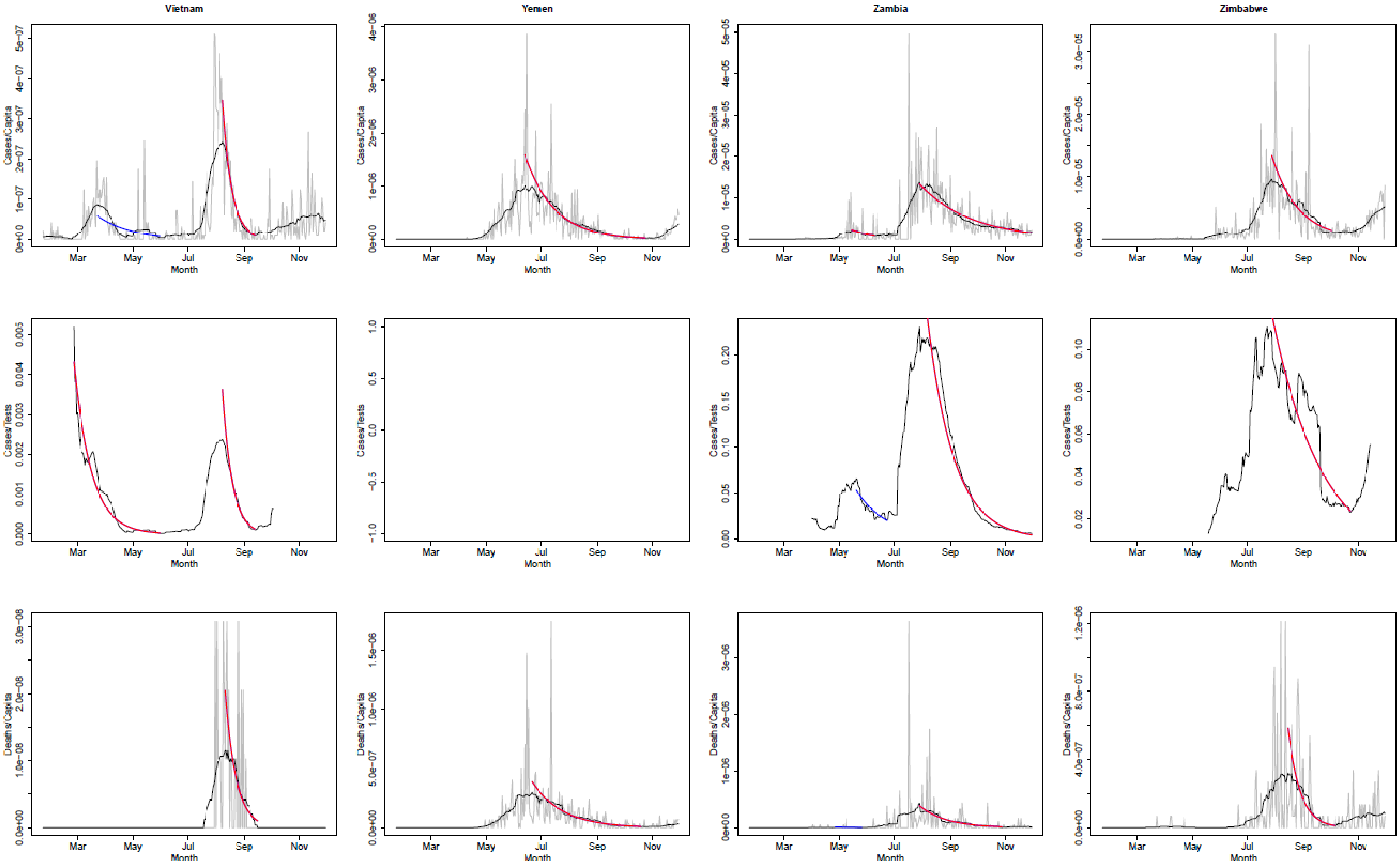
Vietnam
Yemen
Zambia
Zimbabwe
Cases/Capita
Cases/Tests
Deaths/Capita
Mar
May
Jul
Sep
Nov
Month

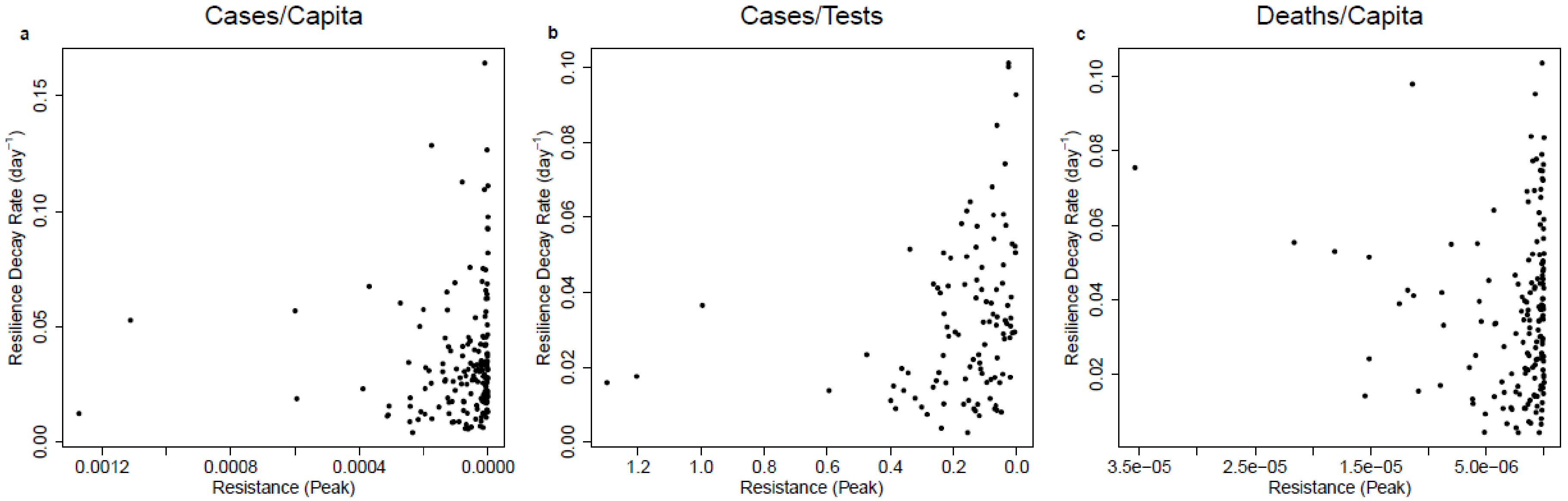

Supplementary Figure 2. Comparing resistance (peak height) and resilience (recovery rate) for: **a.** cases/capita (n=177, ρ=0.22, p<0.01). **b.** cases/tests (n=105, ρ=0.35, p<0.001). **c.** deaths/capita (n=159, ρ=0.18, p<0.05). Note that lower peak height corresponds to higher resistance hence the x-axis scale is inverted. Note also that cases/tests can exceed 1 because of smoothing of the timeseries and differences in the reporting times of cases and of tests.

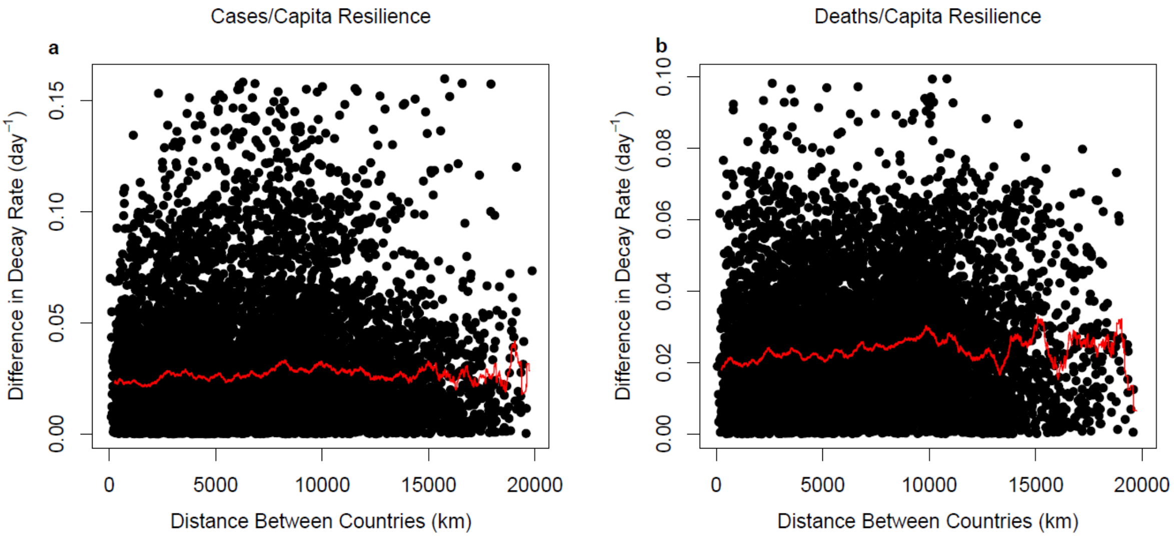

Supplementary Figure 3. Variograms of distance between countries and difference in resilience for: **a.** cases/capita (n=10153, ρ=0.059, p<0.0001). **b.** deaths/capita (n=8385, ρ=0.081, p<0.0001). Red line shows moving average of the data.

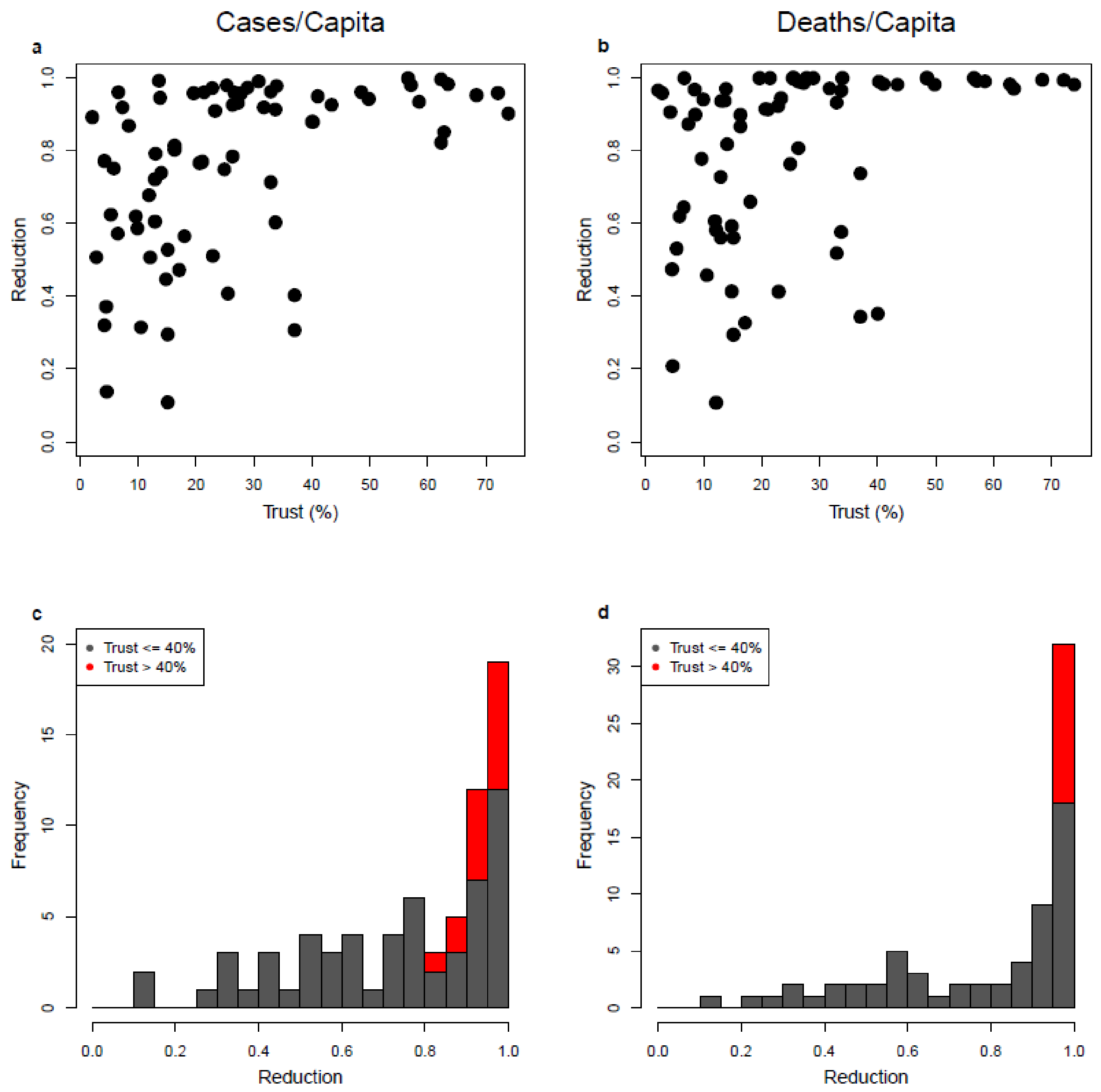

Supplementary Figure 4. Relationships between generalised trust within society and reduction of COVID-19 cases and deaths: **a.** cases/capita (n=72, ρ=0.51, p<0.0001). **b.** deaths/capita (n=72, ρ=0.48, p<0.0001). Stacked frequency distributions partitioning the reduction results into trust ≤40% (grey) and >40% (red): **c.** cases/capita (n=57, n=15; Mann-Whitney U=169, p<0.001). **d.** deaths/capita (n=58, n=14; Mann-Whitney U=132.5, p<0.0001).

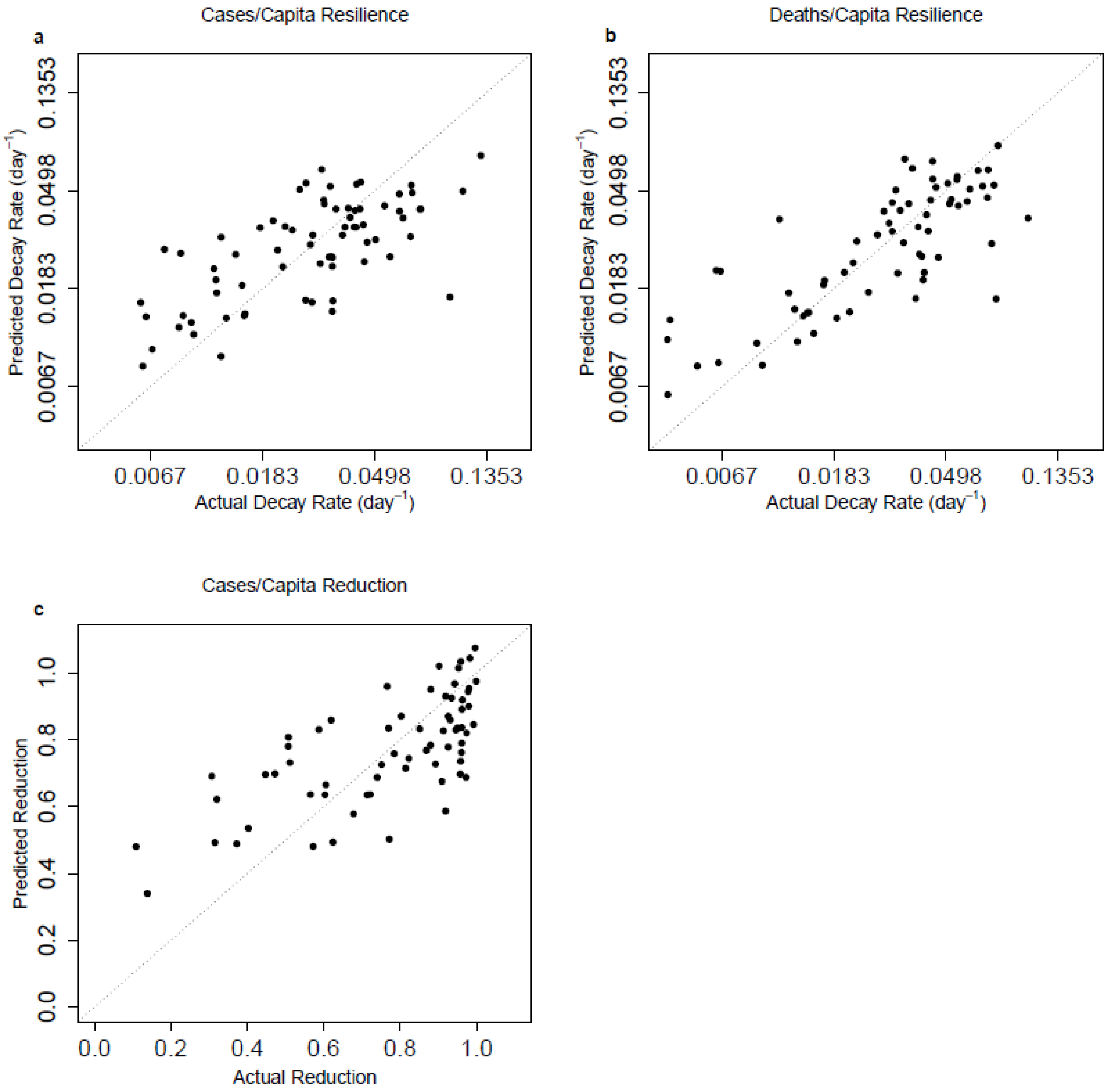

Supplementary Figure 5. Optimised multiple linear regression models including day of year of peak (for comparison to Figure 6 in the main paper). **a.** ln(resilience cases/capita) (n=71, r^2=0.436; Supplementary Table 18). **b.** ln(resilience deaths/capita) (n=67, r^2=0.562; Supplementary Table 19). **c.** cases/capita reduction (n=66, r^2=0.445; Supplementary Table 20). (Note that the deaths/capita reduction model is unaltered from Supplementary Table 7 and Figure 6d.)
